# Exploring Structure–Activity Relationships in Photodynamic Therapy Anticancer Agents Based on Ir(III)-COUPY Conjugates

**DOI:** 10.1021/acs.jmedchem.3c00189

**Published:** 2023-06-02

**Authors:** Anna Rovira, Enrique Ortega-Forte, Cormac Hally, Mireia Jordà-Redondo, Diego Abad-Montero, Gloria Vigueras, Jesús I. Martínez, Manel Bosch, Santi Nonell, José Ruiz, Vicente Marchán

**Affiliations:** † Departament de Química Inorgànica i Orgànica, Secció de Química Orgànica, Universitat de Barcelona (UB), and Institut de Biomedicina de la Universitat de Barcelona (IBUB), Martí i Franquès 1-11, E-08028 Barcelona, Spain; ‡ Departamento de Química Inorgánica, Universidad de Murcia, and Institute for Bio-Health Research of Murcia (IMIB-Arrixaca), E-30100 Murcia, Spain; § Institut Químic de Sarrià, Universitat Ramon Llull, Vía Augusta 390, E-08017 Barcelona, Spain; ∥ Instituto de Nanociencia y Materiales de Aragón (INMA), CSIC-Universidad de Zaragoza, E-50009 Zaragoza, Spain; ⊥ Unitat de Microscòpia Òptica Avançada, Centres Científics i Tecnològics, Universitat de Barcelona, Av. Diagonal 643, E- 08028 Barcelona, Spain

## Abstract

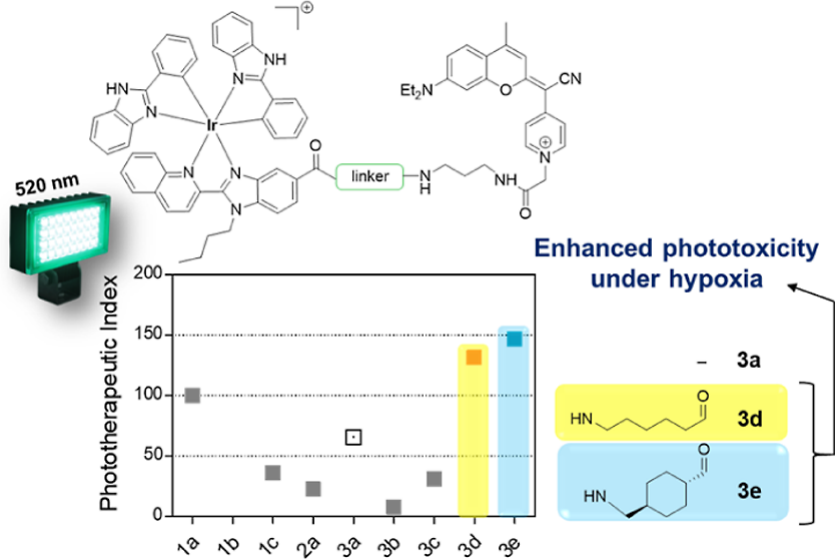

Photodynamic therapy holds great promise as a non-invasive anticancer tool against drug-resistant cancers. However, highly effective, non-toxic, and reliable photosensitizers with operability under hypoxic conditions remain to be developed. Herein, we took the advantageous properties of COUPY fluorophores and cyclometalated Ir(III) complexes to develop novel PDT agents based on Ir(III)-COUPY conjugates with the aim of exploring structure–activity relationships. The structural modifications carried out within the coumarin scaffold had a strong impact on the photophysical properties and cellular uptake of the conjugates. All Ir(III)-COUPY conjugates exhibited high phototoxicity under green light irradiation, which was attributed to the photogeneration of ROS, while remaining non-toxic in the dark. Among them, two hit conjugates showed excellent phototherapeutic indexes in cisplatin-resistant A2780cis cancer cells, both in normoxia and in hypoxia, suggesting that photoactive therapy approaches based on the conjugation of far-red/NIR-emitting COUPY dyes and transition metal complexes could effectively tackle in vitro acquired resistance to cisplatin.

## Introduction

Photodynamic therapy (PDT) has gained great attention in recent years as a promising cancer treatment modality.^1,2^ Unlike conventional approaches based on surgery or chemotherapy, PDT offers several advantages including low invasiveness, high selectivity and efficiency, and reduced toxic side effects that usually compromise patient’s health. Upon light irradiation, the interaction of a non-toxic photosensitizer (PS) and oxygen triggers local cytotoxicity through the generation of reactive oxygen species (ROS), which oxidize biomolecules in cells and lead to irreversible damage on tumor cell structures (e.g., membranes and organelles), as well as on the vasculature that deprives the tumor of oxygen and nutrients.^3^ In addition, increasing evidences show that PDT can trigger the activation of the anticancer immune system throughout the body by immunogenic cell death (ICD).^4,5^ Depending on the reaction mechanism, PDT can be classified into two main types (I and II).^6^ While type II pathway involves the transformation of molecular oxygen (^3^O_2_) into singlet oxygen (^1^O_2_) via an energy transfer process, several other cytotoxic reactive species, mainly superoxide radicals (O_2_^•–^), hydroxyl radicals (^•^OH), and hydrogen peroxide, are generated in type I photochemical pathways via an electron transfer mechanism.^7^ Although type II photochemical processes are generally considered as the main photosensitization mechanism of most of the conventional PSs, the availability of PDT agents operating at low-oxygen concentrations in the phototherapeutic window (e.g., 650–800 nm) is highly desirable to combat deep-seated hypoxic tumors and to avoid toxicity associated to short wavelengths of light.^8−10^

Transition metal complexes have emerged as promising therapeutic tools in photopharmacology due to several unique properties, including a wide range of coordination numbers, oxidation states, and geometries.^11,12^ Among transition metals, Pt(IV), Ru(II), Rh(III), Ir(III), and Os(II) are very attractive candidates for PDT applications since they tend to absorb in the visible region of the electromagnetic spectrum and exhibit relatively high photostability and long luminescence lifetimes (>100 ns), being an interesting alternative to PSs based on organic fluorophores on clinical use such as porphyrins or chlorins.^13−24^ In this context, cyclometalated iridium(III) complexes show excellent anticancer activities and a great potential to overcome some of the main drawbacks of conventional platinum-based chemotherapy (i.e., resistance and toxic side effects).^25,26^ Such metal complexes are likely good candidates for PDT applications as they combine appealing photophysical and photochemical properties within a single compound, including large Stokes’ shifts, high luminescent quantum yield, and high efficient singlet oxygen production upon light irradiation. Photosensitizers based on cyclometalated Ir(III) complexes have been used also as photocatalysts in systems for photocatalytic hydrogen evolution reactions.^27,28^ However, most reported cyclometalated iridium(III) complexes are quite cytotoxic in the dark and only activatable with short wavelengths of light, which compromises further development of efficient PDT agents.

**Figure 1:**
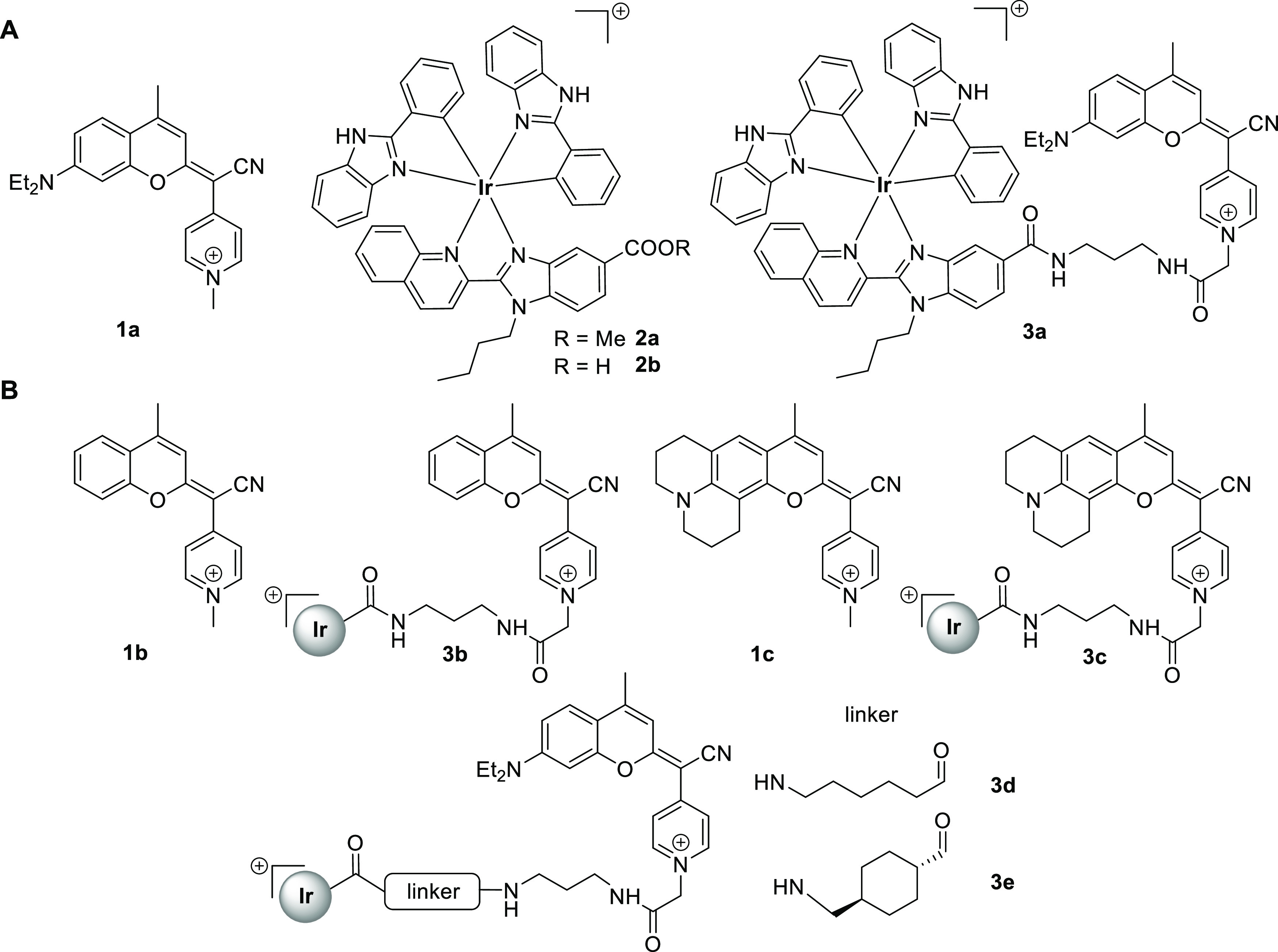
(A) Structure of previously evaluated compounds: COUPY coumarin **1a**, cyclometalated Ir(III) complex **2a,** and the corresponding Ir(III)-COUPY conjugate **3a**. (B) Structure of the new COUPY coumarins (**1b–1c**) and Ir(III)-COUPY conjugates (**3b–3e**) investigated in this work.

By taking advantage of the well-established anticancer properties of transition metal complexes and of the rich and tunable photophysical and physicochemical properties of small organic chromophores, their conjugation can be exploited for developing theranostic agents for imaging-guided PDT. Examples of this strategy include the conjugation and/or integration of cyclometalated Ir(III) complexes with organic fluorophores such as boron-dipyrromethene (BODIPY)^29−32^ porphyrin,^33^ xanthene,^34^ and rhodamine derivatives.^35^ Coumarins, which are also well-known anticancer scaffolds^36−38^ and the basis for the development of novel organic fluorophores,^39^ have also been covalently attached to Ir(III) complexes and used as cyclometalating ligands.^40−43^ In this context, we have been pioneers in describing a novel class of PDT agents based on the conjugation of a far red-emitting COUPY coumarin to a cyclometalated Ir(III) complex (compounds **1a** and **2a**, respectively, in Figure 1).^44,45^ The resulting Ir(III)-COUPY conjugate (compound **3a**) was found to be non-cytotoxic in the dark but highly photocytotoxic after irradiation with visible light, even under hypoxia, the latter being attributable to the selective generation of type I superoxide anion radicals.^44^ Owing to a strong push–pull character due to the replacement of the carbonyl group of the lactone in the conventional coumarin scaffold by N-alkylated cyano(4-pyridine)methylene moieties, COUPY dyes possess several attractive features for bioimaging applications such as absorption and emission within the phototherapeutic window, brightness, high photostability, and large Stokes’ shifts.^46−49^ In addition, we have recently demonstrated that some COUPY dyes exhibit effective in vitro anticancer activities upon visible-light irradiation both under normoxia and hypoxia conditions, while exhibiting minimal toxicity toward normal cells, which position them as promising PS candidates for anticancer PDT.^50^

Herein, we have synthesized a family of new Ir(III)-COUPY conjugates (compounds **3b–3e**) with the aim of exploring structure–activity relationships (SARs), specially to investigate how the structural modifications within the COUPY scaffold influence the photophysical, photochemical, and biological properties of the resulting PSs. As shown in Figure 1, the newly synthesized Ir(III)-COUPY conjugates combine the highly potent cyclometalated Ir(III) complex **2a** and three COUPY derivatives (**1a–1c**), which were connected through flexible or rigid linkers. The absence of the *N*,*N*-diethylamino electron-donating group (EDG) at the 7-position of the coumarin moiety (**1b**) could provide insights on the involvement of this group on the generation of ROS in the resulting Ir(III)-COUPY conjugate **3b**. Moreover, the incorporation of a julolidine-fused analogue (**1c**) in Ir(III)-COUPY conjugate **3c** was expected to prevent the twisted intramolecular charge transfer (TICT) state by incorporating the nitrogen atom into a system of fused rings,^51^ which would also influence electron charge transfer between the metal center and the coumarin moiety. Besides investigating the photophysical and photochemical properties of all of the compounds, the results from cellular uptake and cyto- and phototoxicity studies in several cancer cells allowed us to select two Ir(III)-COUPY conjugates (**3d** and **3e**) as promising PSs owing to their excellent phototoxicities in both normoxic and hypoxic conditions upon green light irradiation and reduced in vitro dark toxicity.

## Results

### Synthesis and Characterization of Ir(III)-COUPY Conjugates

#### Synthesis of Ir(III)-COUPY Conjugate **3b**

**Scheme 1:**
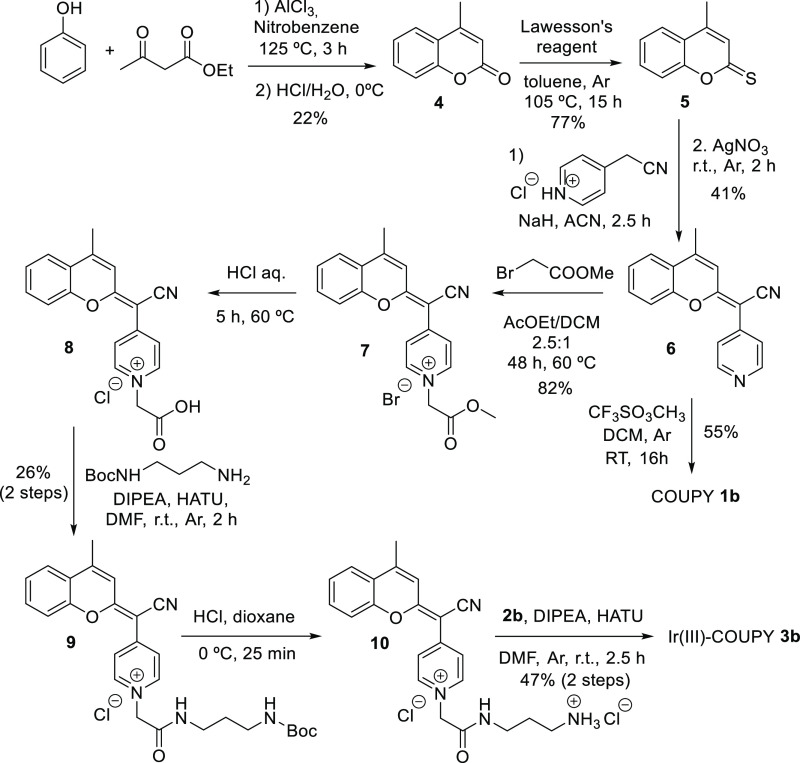
Synthesis of Ir(III)-COUPY Conjugate **3b** and of Control COUPY Coumarin **1b**

As shown in Scheme 1, a convergent approach was used for the synthesis of Ir(III)-COUPY conjugate **3b,** in which the carboxylic group of the Ir(III) complex **2b** (Figure 1) was linked to the amino group of coumarin **10** through the formation of an amide bond. As previously indicated, the synthesis of a COUPY derivative lacking the dialkylamino group at the 7-position of the coumarin skeleton was proposed to explore the contribution of this EDG to ROS generation when conjugated to the metal complex.

The synthesis route for COUPY derivative **10** (Scheme 1) involved seven steps starting with a Pechmann condensation between phenol and ethyl acetoacetate that afforded the desired coumarin skeleton (**4**).^52^ Next, coumarin **4** was reacted with Lawesson’s reagent (LW) to provide thiocoumarin **5**,^53^ which was condensed with 4-pyridylacetonitrile to give compound **6**. Coumarin ester **7** was obtained with good yield by alkylation of **6** with methyl bromoacetate. After acid hydrolysis, the carboxyl group of coumarin **8** was reacted with *N*-Boc-1,3-propanediamine hydrochloride with the assistance of HATU coupling reagent and DIPEA to yield coumarin **9**, whose Boc-protecting group was removed under acidic conditions to give coumarin derivative **10**. Finally, Ir(III)-COUPY conjugate **3b** was obtained as a dark yellow solid after attachment of the Ir(III) complex **2b** to the fluorophore via the formation of an amide bond. All of the compounds depicted in Scheme 1 were purified by silica column chromatography and fully characterized by high-resolution mass spectrometry (HRMS) and ^1^H and ^13^C NMR spectroscopy.

#### Synthesis of Ir(III)-COUPY Conjugate **3c**

**Scheme 2:**
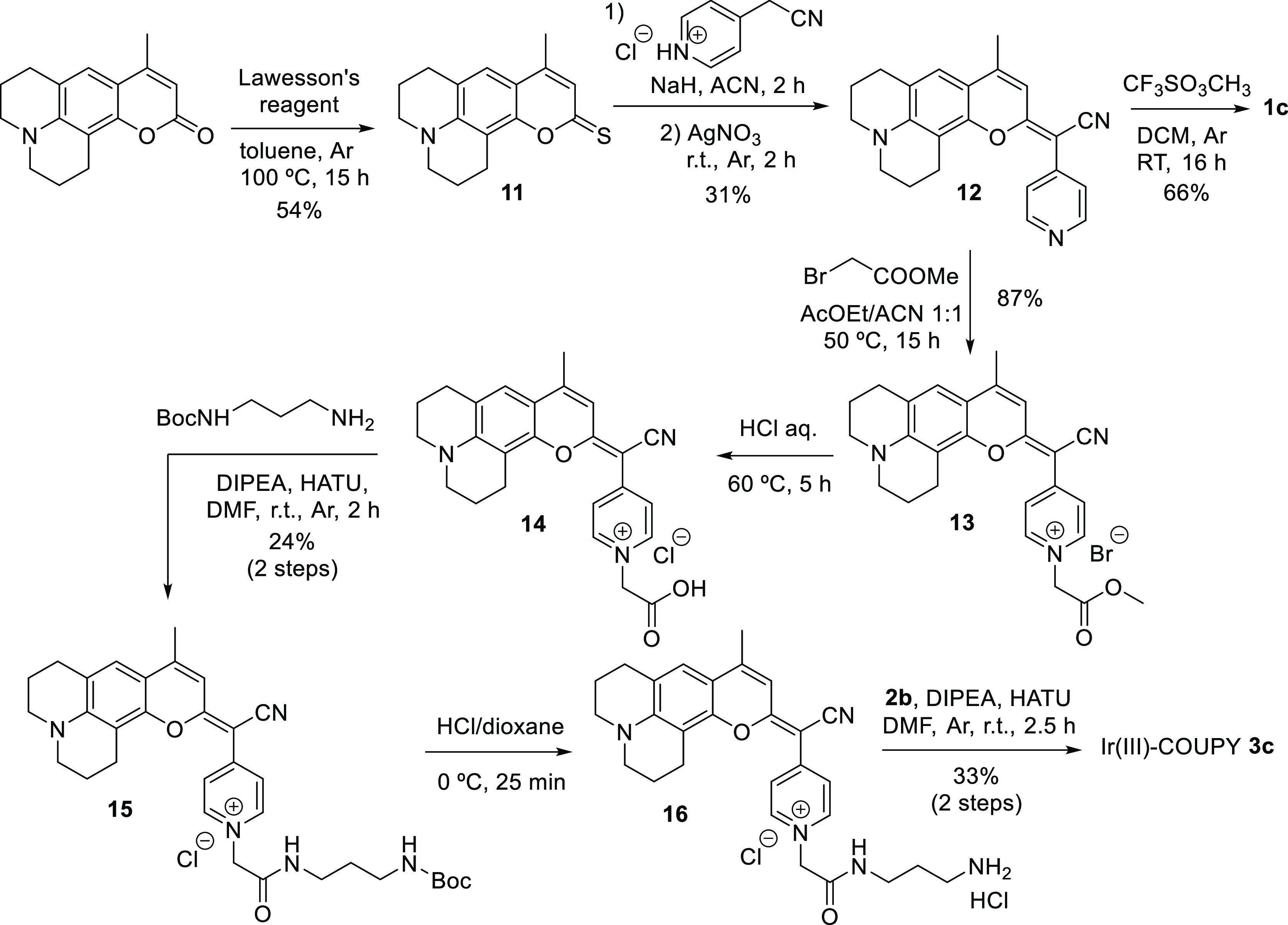
Synthesis of Ir(III)-COUPY Conjugate **3c** and Control COUPY Coumarin **1c**

For the synthesis of Ir(III)-COUPY conjugate **3c** (Scheme 2), we selected a julolidine-fused coumarin analogue to red-shift the absorption maximum with respect to the parent 7-dialkylaminocoumarin. Moreover, as previously stated, rigidification of the amino group was anticipated to have an impact on the photophysical and photochemical properties of the compounds since rotation around the N–C bond is not possible because of the fusion of the six-membered alkyl rings to the aromatic ring. The required amino-containing COUPY derivative **16** was synthesized from a commercially available coumarin precursor following the same procedure as for COUPY **10**. The conjugation between **16** and the Ir(III) complex **2b** afforded Ir(III)-COUPY conjugate **3c** as a dark blue solid after silica column purification. All the compounds were characterized by ^1^H and ^13^C NMR spectroscopy and HRMS.

#### Synthesis of Ir(III)-COUPY Conjugates **3d** and **3e**

**Scheme 3:**
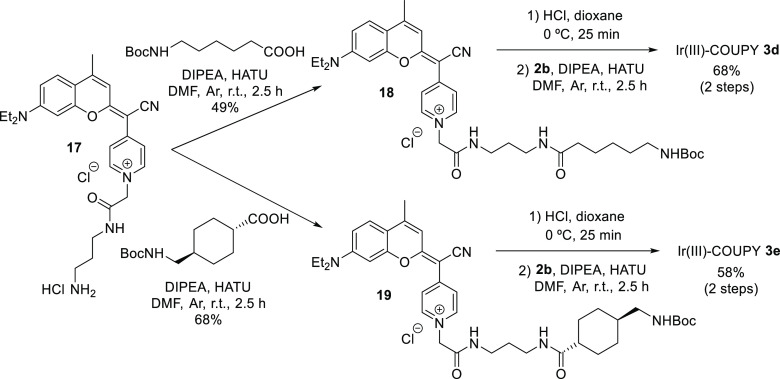
Synthesis of Ir(III)-COUPY Conjugates **3d** and **3e**

With the aim of investigating the effect of the distance between the COUPY fluorophore and the Ir(III) complex in the parent Ir(III)-COUPY conjugate **3a** on the photophysical and photochemical properties of the compounds, we designed two analogues incorporating a longer spacer between both moieties. As shown in Scheme 3, we selected flexible (**3d**) and rigid (**3e**) spacers with the same number of atoms separating the two moieties. The incorporation of both linkers was carried out through the formation of an amide bond between the carboxylic group of each Boc-protected precursor and the free amino group of coumarin **17**,^37^ which afforded COUPY derivatives **18** and **19** containing the flexible and rigid spacers, respectively. Finally, after acidic Boc removal, the conjugation of coumarins **20** and **21** to the Ir(III) complex **2b** provided Ir(III)-COUPY conjugates **3d** and **3e**, respectively, as dark blue solids after silica column purification, which were characterized by ^1^H and ^13^C NMR spectroscopy and HRMS.

#### Synthesis of Control COUPY Coumarin **1b** and **1c**

For the synthesis of the corresponding N-methylated COUPY dyes **1b** and **1c** to be used as control compounds, coumarin **6** (Scheme 1) or coumarin **12** (Scheme 2) were reacted with methyl trifluoromethanesulfonate in DCM at room temperature, yielding the expected compounds as yellow and dark blue solids, respectively.

### Photophysical and Photochemical Characterization of the Compounds

**Figure 2:**
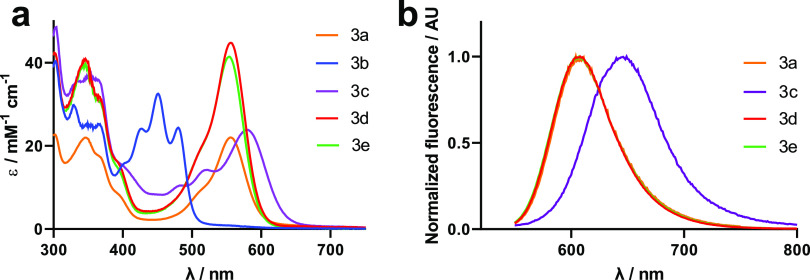
Comparison of the molar absorptivity (a) and emission spectra (b) of Ir(III)-COUPY conjugates in ACN.

The photophysical and photochemical properties [absorption and emission spectra, molar absorption coefficients (ε), fluorescence (Φ_F_) or phosphorescence (Φ_P_) quantum yields, fluorescence (τ_F_) or phosphorescent (τ_P_) lifetimes, and singlet oxygen quantum yield (Φ_Δ_)] of the four new Ir(III)-COUPY conjugates (**3b–3e**) along with the two new coumarins (**1b–1c**) were studied in three solvents of different polarities (phosphate-buffered saline PBS, ACN and DCM), and compared with those of the previously-reported parent compounds (COUPY dye **1a**, Ir(III) complex **2a** and Ir(III)-COUPY conjugate **3a**).^44^ The UV–vis absorption and emission spectra are shown in Figures 2 and S1 and S2, and their photophysical properties are summarized in Tables S1 and S2.

As shown in Table S1, the structural modifications carried out within the coumarin scaffold had a strong impact on the photophysical properties of the compounds. Indeed, the newly synthesized COUPY dyes showed either a bathochromic shift (about 20–30 nm for coumarin **1c**) or a hypsochromic shift (about 100–120 nm for coumarin **1b**) in their absorption and emission maxima compared with those of the parent coumarin **1a** (e.g. in ACN, λ_abs_ = 548 nm and λ_em_ = 609 nm for **1a** vs λ_abs_ = 446 nm and λ_em_ = 492 nm for **1b**, and λ_abs_ = 572 nm and λ_em_ = 635 nm for **1c**). The replacement of the *N*,*N*-dialkylamino group at position 7 of the coumarin skeleton by the strong electron-donating julolidine-fused analog in coumarin **1c** caused the observed red-shifting in the wavelength at the absorption and emission maxima. On the contrary, the absence of an EDG at position 7 in coumarin **1b** partnering with the electron-withdrawing cyanomethylenepyridinium moiety had a negative effect on the spectroscopic properties of the fluorophore since both absorption and emission maxima were strongly blue-shifted due to the decreased push–pull character of the chromophore. On the other hand, the fluorescent quantum yields were slightly lower for coumarin **1c** in DCM and PBS, compared with the original COUPY **1a**, whereas coumarin **1b** was found to be very weakly fluorescent. For coumarins **1a** and **1c**, a strong decrease in the fluorescence quantum yield and lifetime was observed in the more polar solvents, indicating the onset of efficient intramolecular electron-transfer excited-state deactivation pathways. Consistent with this, coumarin **1b**, lacking the *N*,*N*-dialkylamino EDG, showed a less pronounced solvent dependence.

As we had already reported,^44^ the Ir(III) complex **2a** showed a strong and long-lived phosphorescence band around 660 nm, whose intensity and lifetime decreased in the Ir(III)-COUPY conjugate **3a**, suggesting the existence of competitive excited-state processes. As shown in Table S2, a similar behavior was observed in the three new conjugates incorporating a far-red emitting coumarin (**3c–3e**). Among them, conjugate **3c** containing the julolidine-fused coumarin showed the largest red-shift in the absorption and emission maxima (∼25–30 nm depending on the solvent), which parallels the spectroscopic properties of COUPY **1c** (**3c**, λ_abs_ = 580 nm and λ_em_ = 647 nm in ACN). Interestingly, the luminescent quantum yield of **3c** was higher than that of the parent conjugate **3a** in DCM, but similar values were obtained in ACN and PBS. As expected, conjugates **3d** and **3e** containing the flexible and the rigid spacer, respectively, showed similar absorption and emission maxima in all investigated solvents compared to the original conjugate **3a** since they contain the same coumarin (about λ_abs_ = 555 nm and λ_em_ = 610 nm in ACN). The luminescence quantum yield values were also similar for the three conjugates, about 0.1 in DCM and ACN, and below 0.02 in PBS. The strong decrease in the luminescence quantum yields and lifetimes in PBS is consistent with the onset of efficient competing intra- and inter-chromophore electron-transfer processes. In good agreement with the behavior of COUPY dye **1b**, the Ir(III)-COUPY conjugate **3b** practically did not show any fluorescence in all of the solvents investigated and, as shown in Table S2, only phosphorescence lifetime values associated with the Ir(III) complex could be determined and these were shorter than for **3a**. Finally, it is worth noting that the maximum absorption wavelengths of all the compounds (COUPY dyes and Ir(III)-COUPY conjugates) in different solvents gradually decrease according to the following order DCM > ACN > PBS, which agrees with a negative solvatochromism phenomenon (Tables S1 and S2).

Next, we focused on investigating the impact of the structural modifications on the generation of ROS by Ir(III)-COUPY conjugates. As we previously reported,^44^ the Ir(III) complex **2a** produced singlet oxygen in all of the organic solvents evaluated but not in PBS as a result of a very efficient formation of the triplet state due to the heavy-atom effect, while COUPY **1a** did not show significant ^1^O_2_ quantum yields in any solvent (Table S1). However, the conjugation of Ir(III) complex **2a** to the COUPY fluorophore **1a** led to an increase of ^1^O_2_ quantum yield by one order of magnitude, suggesting a higher population of COUPY triplet excited states (T_1_) due to either an enhanced intersystem crossing (ISC) process in the COUPY moiety of conjugate **3a** when directly excited at 532 nm (as demonstrated by the shorter fluorescence lifetime), or an efficient triplet–triplet energy transfer process from the Ir(III) complex to COUPY when the complex is excited at 355 nm (as demonstrated by its shorter phosphorescence lifetime). In either case, since the triplet lifetime of COUPY is longer than that of the Ir(III) complex, localization of the triplet excited-state energy in the COUPY moiety provides more time for energy transfer to ^3^O_2_, thereby favoring the production of ^1^O_2_ (Table S2). The ability of all newly synthesized Ir(III)-COUPY conjugates (**3b–3e**) and control COUPY fluorophores (**1b–1c**) to produce ^1^O_2_ was evaluated spectroscopically by the observation of ^1^O_2_ emission at 1275 nm upon excitation at two wavelengths (355 and 532 nm). In the same way as for the original COUPY derivative **1a**, neither **1b** nor **1c** generated significant amounts of ^1^O_2_ at either excitation wavelength, indicating that radiative decay competes efficiently with intersystem crossing to the triplet excited state, the precursor of singlet oxygen. Interestingly, ^1^O_2_ emission was observed in all new Ir(III)-COUPY conjugates in DCM and ACN irrespective of which chromophore was initially excited, but not in PBS, consistent with the observed decrease in luminescence quantum yields and lifetimes as a result of efficient electron-transfer competing processes. Conjugates **3d** and **3e** containing the same coumarin derivative as the parent compound (**3a**) showed similar singlet oxygen quantum yields (ca. 0.30–0.40 in DCM upon excitation at both wavelengths) regardless of the spacer linking both moieties, while the conjugates with new coumarin derivatives, **3b** and **3c**, exhibited lower values (ca. 0.20 in DCM).

**Figure 3:**
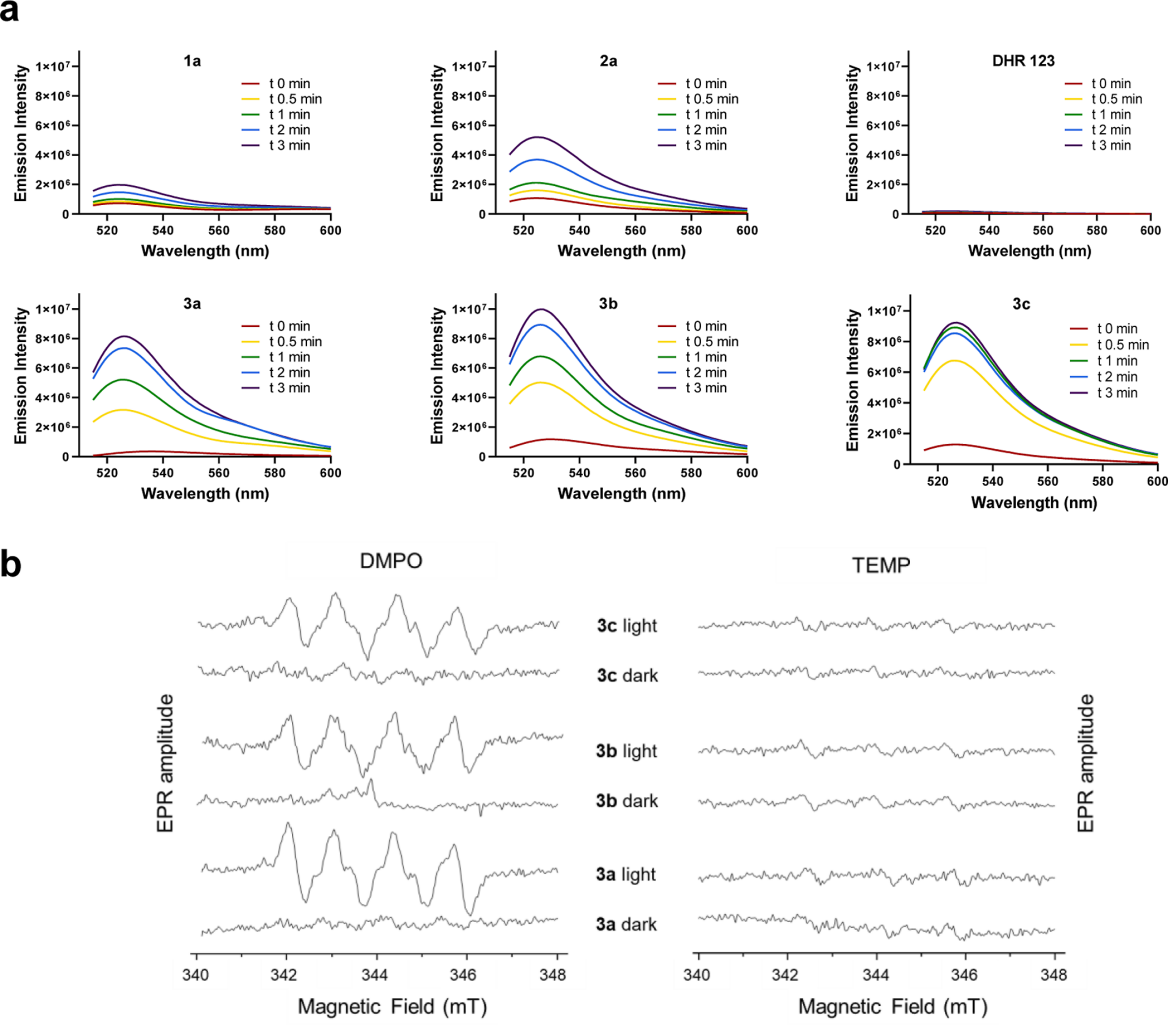
Detection of ROS in solution. (a) Increase of the fluorescence spectra emission of DHR123 upon photoirradiation of COUPY coumarin **1a**, Ir(III) complex **2a**, Ir(III)-COUPY conjugates **3a**, **3b,** and **3c** or without any compound (DHR 123 alone) at 505 nm in PBS (0.2% DMSO). DHR123 fluorescence was excited at 500 nm. (b) EPR spectra of Ir(III)-COUPY conjugates **3a–3c** trapped by DMPO or TEMP in MeOH in the dark and after 5 min of irradiation with green light (530 nm and 450 mW cm^–2^).

Considering that one of the main features of the parent Ir(III)-COUPY conjugate **3a** is the generation of superoxide anion radical (O_2_^•–^) in living cells upon irradiation with visible light,^44^ we next investigated the ability of the new conjugates to produce this specific type-I ROS in PBS by using a spectroscopic method based on the oxidation of the non-fluorescent dihydrorhodamine 123 (DHR123) probe by O_2_^•–^ to the corresponding fluorescent rhodamine 123 derivative. As shown in Figures 3 and S3, Ir(III)-COUPY conjugates did not produce any measurable quantity of superoxide anion radical before irradiation; this result is comparable to that obtained with DHR123 alone after irradiation, which was used as a negative control. Surprisingly, under green light irradiation (505 nm), all of the new conjugates, including the one containing the coumarin fluorophore lacking the amino group at the 7-position, clearly increased the fluorescence intensity of DHR123 to a greater extent than the Ir(III) complex **2a** and COUPY derivatives **1a–1c**, indicating the generation of superoxide anion radical, consistent with the observed excited-state electron-transfer processes. Remarkably, all the new conjugates led to a faster O_2_^•–^ generation rate in comparison to the parent compound **3a**. The Ir(III)-COUPY conjugate containing the julolidine-fused system (**3c**) showed the fastest rates in superoxide generation, thereby demonstrating the importance of incorporating a strong EDG in the coumarin scaffold, which not only red-shifts absorption and emission maxima but also produces more O_2_^•–^. Interestingly, the Ir(III)-COUPY conjugate **3b** reached the highest maximum emission intensity of superoxide generation after 3 min of irradiation, indicating that the amino group at the 7-position of the coumarin moiety is not strictly necessary to trigger the generation of O_2_^•–^. Overall, the suppression of singlet oxygen generation in PBS, and the reduction in fluorescence and phosphorescence lifetimes and quantum yields of Ir(III)-COUPY conjugates, are consistent with the onset of intra- and inter-chromophore electron-transfer processes, resulting in the generation of superoxide radical anion.

Further evidence of the generation of O_2_^•–^ was obtained by electron paramagnetic resonance (EPR) using the DMPO spin trap. As shown in Figure 3b, the characteristic paramagnetic signal for the DMPO-O_2_^•–^ adduct (peak integral ratio 1:1:1:1) was observed upon irradiation with 530 nm LED light, which confirmed that Ir(III)-COUPY conjugates **3a–3c** generate O_2_^•–^ regardless of the modification introduced in the coumarin scaffold. By contrast, no signal was observed in the absence of light, thereby indicating again that O_2_^•–^ generation is a light-promoted process. Moreover, EPR studies with TEMP spin trap demonstrated that none of the Ir(III)-COUPY conjugates was able to photosensitize singlet oxygen since no signal for the expected TEMP-^1^O_2_ adduct was observed when the mixture was irradiated (Figure 3c), which agrees with the results of spectroscopic studies in polar protic solvents (Table S2).

### Cellular Uptake and Localization of Ir(III)-COUPY Conjugates

**Figure 4:**
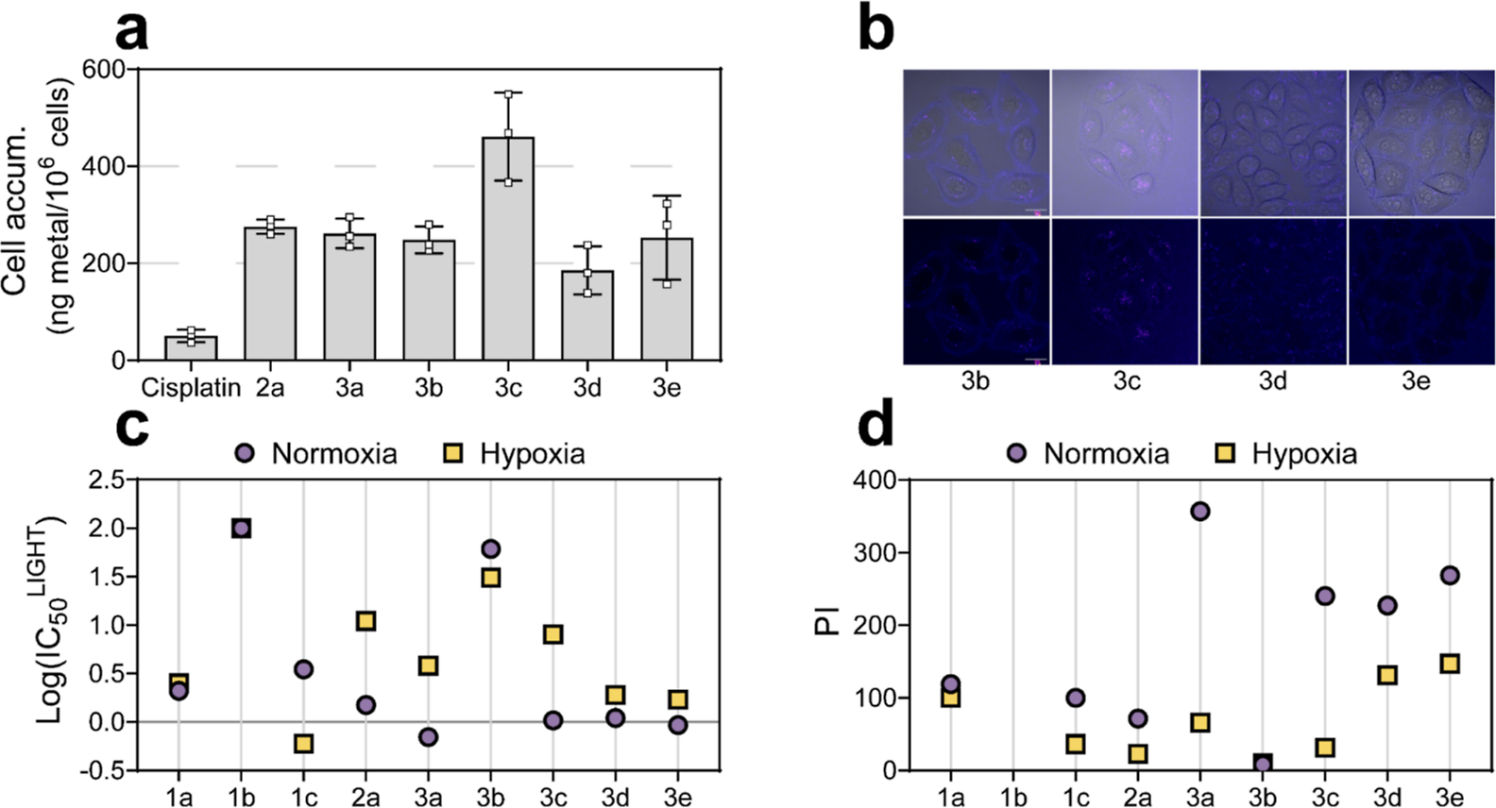
Cellular uptake and photocytotoxicity of Ir-COUPY conjugates in cancer cells. (a) Intracellular accumulation of Ir(III) compounds and cisplatin in A2780cis cells after 2 h treatment at 10 μM. Data expressed as mean ± SD from three independent measurements. (b) Single confocal planes of HeLa cells incubated with the compounds for 30 min at 37 °C. Upper row: Merge of bright field and fluorescence images. Lower row: Fluorescence images of the compounds. Scale bar: 20 μm. (c–d) Summary of in vitro photocytotoxicity for compounds **1a–1c**, **2a,** and **3a–3e** in A2780cis cells after light irradiation (520 nm, 1.5 mW cm^–2^, 1 h) under normoxia (21% O_2_) or hypoxia (2% O_2_) represented as log(IC_50_^LIGHT^) and phototherapeutic indexes (PI), defined as the ratio of dark to light IC_50_ values. IC_50_, PI values, and their corresponding SD errors are listed in the Supporting Information (Table S3).

The cellular uptake of the conjugates was first investigated using inductively coupled-plasma mass spectrometry (ICP–MS). After 2 h incubation, the iridium content inside cancer cells treated with Ir(III)-COUPY conjugates at 10 μM yielded similar results, with metal accumulations 4- to 9-fold higher than those found in cisplatin-treated cells (Figure 4a). Intracellular Ir levels varied from 185 ± 40 to 261 ± 25 ng/10^6^ cells for Ir(III)-COUPY-treated cells and were comparable to those incubated with Ir(III) complex **2a** (276 ± 12). Strikingly, the amount of metal in cells treated with conjugate **3c** doubled that (461 ± 74 ng Ir per million cells), indicating that the julolidine-fused system of **3c** greatly improved cellular internalization.

To gain more insights on cellular uptake, the compounds were incubated with living cancer cells and examined under a confocal microscopy using yellow light laser (λ_ex_ = 561 nm) excitation. As shown in Figure 4b, fluorescence signals from all of the new conjugates containing a far-red emitting coumarin (e.g. **1a** or **1c**), compounds **3c–3e**, were appreciated inside cells, thereby confirming an excellent cellular uptake. By contrast, the lack of fluorescent emission prevented the cellular uptake of conjugate **3b** to be studied by confocal microscopy. In agreement with ICP–MS results, intracellular fluorescence was slightly higher in **3c**-stained cells than with other conjugates (Figure 4a). Overall, the Ir(III)-COUPY conjugates **3c–3e** showed the same internalization pattern as the parent conjugate **3a**,^44^ characterized by fluorescent vesicles in the cytoplasm, which contrasts with that of the unconjugated COUPY coumarins that typically accumulate in mitochondria (e.g., compound **1a**).^47^

### Photobiological Studies

Having shown that the Ir(III)-COUPY conjugates can sensitize both type I and II ROS through spectroscopic techniques and readily internalize into living cells, their photocytotoxicity was screened in various cancer cell lines. This screening included two representative melanoma cell lines (female human A375 and male human SK-MEL-28) and cancer cells with resistance to the clinical drug cisplatin. For the latter, both sensitive and cisplatin-resistant ovarian cancer cells (A2780 and A2780cis) as well as HeLa cells, which also show a degree of chemoresistance to the platinum anticancer drug in vitro,^54^ were used. For the determination of dark and light cytotoxicity, dose–response curves were assayed from both conditions to provide the correspondent IC_50_ values, which correspond to the concentration needed for inhibition of cell growth by 50%. Phototherapeutic index (PI) was calculated as the ratio of dark to light IC_50_ value for each compound.

#### Dark cytotoxicity

Except for compound **1c**, all the compounds were found non-toxic in the absence of the light trigger (IC_50_ > 100 μM) regardless of the cell line (Tables 1, S3 and S4). From this, it was clear that the julolidine-fused system of **1c** impacted on dark cytotoxicity, rendering dark IC_50_ values that oscillated between 5 and 26 μM in the studied cancer cell lines. Noteworthy, conjugate **3c**, which also contains **1c** as a coumarin moiety showed no dark cytotoxicity up to 100 μM.

**Table 1:** Photocytotoxicity of the Compounds toward Cancer Cells in Normoxia^a^

	A375		SK-MEL-28		HeLa		A2780		A2780cis	
	dark	light	dark	light	dark	light	dark	light	dark	light
**1a**	>100	1.1 ± 0.1 [>89]	>100	1.2 ± 0.1 [>86]	>100	5.8 ± 0.4 [>17]	>100	5.2 ± 0.5 [>19]	>100	2.1 ± 0.2 [>48]
**1b**	>100	10 ± 2 [>10]	>100	>100 [n.d.]	>100	>100 [n.d.]	>100	>100 [n.d]	>100	>100 [n.d.]
**1c**	9.9 ± 0.5	0.31 ± 0.02 [32]	12 ± 1	0.2 ± 0.01 [60]	26 ± 4	1.1 ± 0.8 [24]	5.1 ± 0.9	0.09 ± 0.01 [57]	15 ± 2	0.15 ± 0.04 [100]
**2a**	>100	2.1 ± 0.3 [>48]	>100	2.8 ± 0.3 [>36]	>100	75 ± 6 [>1.3]	>100	4 ± 1 [>25]	>100	3.5 ± 0.4 [>29]
**3a**	>100	18 ± 3 [>6]	>100	1.5 ± 0.1 [>67]	>100	8.6 ± 0.7 [>12]	>100	1.07 ± 0.07 [>94]	>100	0.70 ± 0.06 [>143]
**3b**	>100	78 ± 6 [>1.3]	>100	2.1 ± 0.2 [>48]	>100	18 ± 2 [>6]	>100	7.1 ± 0.1 [>14]	>100	61 ± 8 [>1.6]
**3c**	>100	38 ± 5 [>3]	>100	>100 [n.d.]	>100	45 ± 4 [>2.2]	>100	2.1 ± 0.2 [>48]	>100	1.04 ± 0.02 [>96]
**3d**	>100	46 ± 4 [>2.2]	>100	2.5 ± 0.2 [>40]	>100	2.0 ± 0.4 [>50]	>100	1.78 ± 0.07 [>56]	>100	1.1 ± 0.2 [>91]
**3e**	>100	19 ± 3 [>5]	>100	7.6 ± 0.9 [>13]	>100	9.3 ± 0.8 [>11]	>100	1.9 ± 0.2 [>53]	>100	0.93 ± 0.04 [>108]

a Cells were treated for 2 h (1 h of incubation, 1 h of irradiation with green light at 520 nm, 1.5 mW cm^–2^) followed by 48 h incubation in drug-free medium. Dark analogues were kept in the dark. Data expressed as mean ± SD from three independent experiments. *n.d. = not determined. PI (phototherapeutic index) is given in brackets; PI defined as IC_50_ (dark-non-irradiated cells)/IC_50_ (irradiated cells).

#### Photocytotoxicity in Normoxia

First, photoactivation of conjugates **3a–3e** was evaluated in cancer cells, along with their coumarin precursors **1a–1c** and the parent Ir(III) complex **2a**. Light treatments were applied at doses of 1.5 mW cm^–2^ using single green wavelength (520 nm) LED irradiation. Overall, the compounds triggered cell death upon light exposure at 520 nm in all cancer cell lines studied, with IC_50_ values in the low micromolar range (Table 1). COUPY **1b**, which lacks the *N*,*N*-dialkylamino group at position 7, did not display antiproliferative activity after light irradiation toward the investigated cancer cells, probably due to its blue-shifted absorption (Figure S2). In contrast, the original COUPY derivative **1a** exerted higher phototoxic action, particularly against melanoma cells, resulting in more than 86-fold differences in bioactivity upon irradiation (Table 1). The correspondent conjugate (**3b**) showed a slight increase in phototoxic activity compared to **1b**. This might be ascribable to the Ir(III) core of the PS because the Ir(III) complex **2a** also presented reasonable inhibitory activity after irradiation. Nonetheless, the green-light photocytotoxicity of **3b** was lower than that of **3a** in all cancer cell lines (light IC_50_ values between 3 and 78 μM compared to 0.7–18 μM). To test whether the poor phototoxicity of **3b** was due to a blue-shifted absorption, photoactivation upon blue light (465 nm) was also assayed. As shown in Table S4, A2780cis cells dosed with **3b** rendered light IC_50_ values that were very similar to those obtained for **3a** after blue light irradiation (2.4 and 1.9 μM, respectively). On the other hand, replacement of the *N*,*N*-dialkylamino group of the coumarin by a julolidine-fused system decreased the phototoxicity of conjugate **3c** in A375, SK-MEL-28 and HeLa cells compared to **3a**. Nevertheless, the behavior of **3c** after green light irradiation was similar to that of **3a** in A2780 and A2780cis cancer cells, with light IC_50_ values between 0.7 and 2.1 μM.

#### Phototherapeutic Index

To explore the phototherapeutic potential of the Ir(III)-COUPY conjugates, we performed a SAR analysis derived from their phototoxic activity in cancer cells. For the identification of the best performing anticancer PDT agents, PI determination was used. Noticeably, PIs differed from one cancer cell line to another. For instance, A375 melanoma cells, which exhibit the most aggressive melanoma phenotype,^55^ were less sensitive to Ir(III)-COUPY phototoxicities (PI values not exceeding >6) than SK-MEL-28 melanoma cells, where PIs reached up to >67 (Table 1). The conjugates also exerted mild phototoxic activity in HeLa cancer cells (PIs ranging from 2.2 to >50). The order of potency toward cancer cells generally was **3a** > **3c** ≈ **3d** ≈ **3e** > **3b**, with small discrepancies in this trend depending on the cell line. Nevertheless, the most potent green-light photoactivation was found in ovarian cancer cells, particularly in resistant A2780cis cells, where PI values were markedly higher both for the conjugates containing the parent COUPY dye **1a** regardless of the type and length of the spacer, and the one incorporating the julolidine-fused system (>143 for **3a**, >96 for **3c**, >91 for **3d,** and >108 for **3e**). Overall, these results led us to initially select **3a** and **3c–3e** due to their good photocytotoxic profiles, especially in A2780cis cancer cells. However, since **3c** accumulated in cancer cells to a greater extent than **3a** or **3d–3e** (Figure 4a), we hypothesized that **3c** was less efficient as a PS, as it would require a more intracellular amount of compound to induce similar phototoxic outcomes. Therefore, **3a** was considered as the best hit Ir(III)-COUPY candidate for green-light PDT in vitro, followed by **3d** and **3e**, which contain the same coumarin and Ir(III) complex moieties.

#### Photocytotoxicity in Hypoxia

Since A2780cis cells were strongly inhibited by Ir(III)-COUPY photosensitizers upon green light irradiation, this cell line was used for further SARS evaluation. The phototoxic action of the compounds was assessed toward A2780cis cells under hypoxic conditions (2% O_2_) and compared to the clinically approved PS 5-aminolevulinic acid (5-ALA). In this second photocytotoxicity testing, dark cytotoxicity was recalculated using higher concentrations, given that the compounds were deemed as inactive at 100 μM. Except for **1c**, no dark IC_50_ values could be determined either in normoxia or hypoxia up to 250 μM (Table S3), which is highly desirable for PDT agents. As shown in Table S5, although none of them inhibited cell growth completely at 250 μM, some conjugates were relatively toxic at this dose. For instance, conjugates **3a**, **3b,** and **3c** provided 45–76% of cell killing, while linker-containing conjugates **3d** and **3e** barely exhibited dark cytotoxicity at 250 μM (cell growth inhibition between 12 and 27%).

Under hypoxia, poorer PI values were found compared to those under normoxia, which can be explained in terms of photodynamic effect restriction by the lack of oxygen (Figure 4d and Table S3). All the compounds exhibited higher phototherapeutic effects against A2780cis cells than the protoporphyrin IX (PpIX) precursor 5-ALA (Table S3). The highest PI values in normoxia were achieved by **3a** (>357), followed by the conjugates containing the longer linkers **3d** and **3e** (>227 and >269, respectively). However, under hypoxia, the photodynamic effect of **3a** significantly decreased (PI > 66), whereas **3d** and **3e** yielded comparably higher photocytotoxicity, with PI values of >131 and >147, respectively. As depicted in Figure 4d, julolidine-containing conjugate **3c** displayed a similar PI value under normoxia (>240), but was much less photoactive under hypoxia (PI > 31). On the other hand, no obvious photocytotoxicity was observed for **3b** upon green light irradiation neither in normoxia nor in hypoxia (light IC_50_ values of 61 and 31 μM, respectively).

#### Photogeneration of ROS

**Figure 5:**
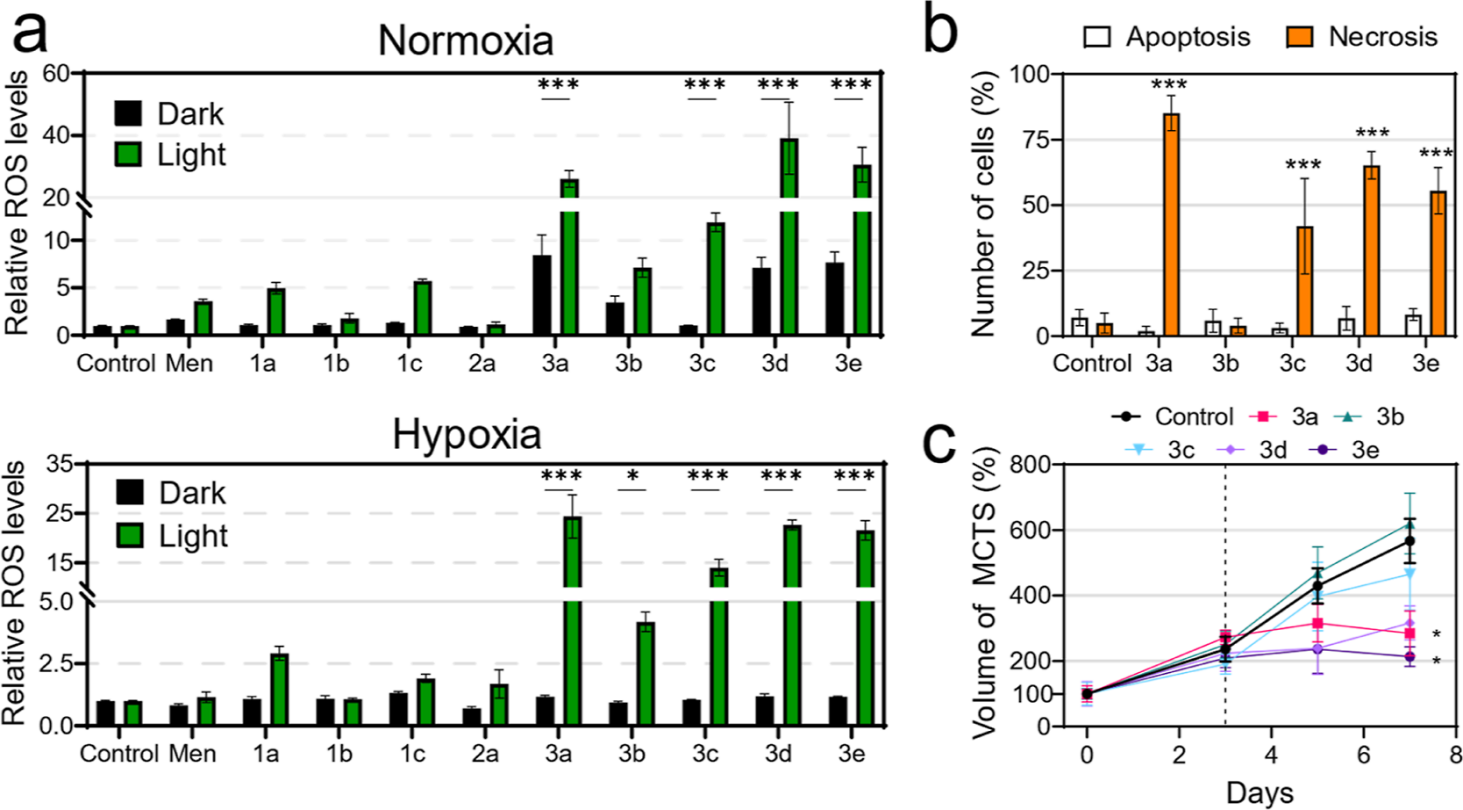
Photoinduced cell death mechanism of Ir-COUPY conjugates. (a) Relative ROS levels in A2780cis cells under normoxia (21% O_2_) and hypoxia (2% O_2_) after treatment with the compounds at 10 μM in dark or upon light irradiation (520 nm, 1.5 mW cm^–2^, 1 h) as measured with the DCFH-DA probe. Menadione (Men, 50 μM) was used as a positive control for ROS induction. Data expressed as mean ± SD from three independent measurements. (b) Cell death induction in A2780cis after PDT treatment with Ir(III)-COUPY agents. Data from three independent flow cytometry experiments using Annexin V/PI dual staining. (c) Change in the relative volume of A2780cis MCTS over 7 days. PDT treatments were applied on day 3 (dashed line). Error bars correspond to SD from three replicates. Statistical significance *p < 0.05, **p < 0.01, ***p < 0.001 from two-way ANOVA test (a,b) or one-way ANOVA test (c).

Once the photocytotoxicity of the compounds against cancer cells was demonstrated, the induction of cellular oxidative stress after irradiation was explored under both normoxia and hypoxia using the ROS probe 2,7-dichlorodihydrofluorescein diacetate (DCFH-DA) (Figure 5). In dark conditions, ROS levels from untreated, control cells remained similar to those treated with COUPY compounds **1a–1c** or with iridium(III) complex **2a** regardless of the oxygen tension. Noticeably, conjugates **3a** and its linker-containing derivatives **3d** and **3e** slightly increased ROS levels under normoxia in the absence of light (Figure 5a). Upon 520 nm light application, a strong fluorescence signal from the DCFH-DA probe was observed in normoxic cells treated with **3a**, **3c**, **3d,** and **3e**, indicating efficient photogeneration of ROS. Although this photogeneration was comparably lower under hypoxia, differences between dark and light conditions were still markedly significant under low oxygen environment. Strikingly, although poor phototoxicity was achieved for **3b** in cellular assays under hypoxia (Figure 4d), a slight increase in ROS levels was found after green-light irradiation in A2780cis cells, these differences being significantly much larger for the other conjugates (Figure 5b).

#### Photoinduced Cell Death Mechanism

The photogeneration of ROS by the Ir(III)-COUPY conjugates prompted us to study the cell death mechanism of these PSs. After 520 nm light irradiation, the compounds increased the number of detached, non-viable cells as well as rounded, floating cells. To characterize these morphological changes, flow cytometry analysis of cell size and cell complexity was performed using forward scatter and side scatter (FSC vs SSC) plots. Irradiated, non-treated A2780cis cells grouped into a defined morphological cell population. However, PDT treatment with Ir(III)-COUPY conjugates induced a second large population of cell particles with low FSC/SCC ratios (Figure S4). Although **3b** did not produce such changes, **3a**, **3c**, **3d,** and **3e** vastly induced these secondary cell populations that correspond to dead cells with non-viable morphology (Figure S4). To discern the mode of cell death induced, the Annexin V/Propidium Iodide (AV/PI) labeling method was used. These assays can detect the translocation of specific phospholipids during apoptosis in AV^+^/PI^–^ and AV^+^/PI^+^ regions and loss of cell membrane integrity from necrosis in AV^–^/PI^+^ cells. As depicted in Figures 5b and S5, **3a**, **3c**, **3d,** and **3e** photoinduced necrotic cell death, as revealed by the increase of cells AV^–^/PI^+^ due to nuclear staining of PI.

#### 3D Tumor Growth Inhibition Effects

To better characterize the photoinduced anticancer activity of the Ir-COUPY conjugates under hypoxic conditions, their photocytotoxicity was assayed against 3D multicellular tumor spheroid (MCTS) culture systems. Cancer cells growing in MCTS cultures display heterogeneous regions, nutrient and oxygen gradients, as well as intercellular and cell-extracellular matrix interactions that mimic the tumor microenvironment.^56,57^ In addition, 3D MCTS represents a more stringent model for drug screening since they allow for assay drug penetration, resistance, and importantly drug response to hypoxic gradients toward the center of the sphere, which are particularly interesting aspects in the development of novel PDT agents.^58,59^

Defined A2780cis MCTS were uniformly generated with an average radius of ∼350 μm and an average volume of ∼0.181 mm^3^ (Figure S6). These volume values were used to normalize MCTS growth. After 3 days, MCTS were incubated with Ir(III)-COUPY conjugates for 4 h and exposed to 1 h of 520 nm light irradiation. In coherence with what we observed in 2D cellular models under hypoxia, PDT treatments with conjugates **3a**, **3d,** and **3e** effectively blocked tumoral growth of the A2780cis spheres, with **3a** and **3e** producing statistically significant shrank compared to irradiated controls (Figures 5c and S7). In contrast, conjugates **3b** and **3c** did not show significant tumor inhibition effects under the same experimental conditions.

## Discussion and Conclusions

PDT holds great promise as a non-invasive anticancer tool against drug-resistant cancers.^60^ However, highly effective, non-toxic, and reliable PSs remain to be developed. Herein, we took the advantageous properties of both organic fluorophores and transition metal-based complexes to develop a novel family of PDT agents. Inspired by our previous encouraging results with this type of compounds,^44^ we synthesized four new PSs based on the conjugation of COUPY coumarins with far-red/NIR emission to a cyclometalated Ir(III) complex.

The photophysical properties of the COUPY fluorophores were highly influenced by the structural modifications carried out within the coumarin scaffold. In particular, the incorporation of a strong EDG at position 7 of the coumarin skeleton in the newly synthesized COUPY dye **1c** through fusion of the julolidine system was found to induce a bathochromic shift in the absorption and emission maxima compared to the 7-*N*,*N*-dialkylamino-containing parent coumarin **1a**. On the other hand, the decreased push–pull character of the π-delocalized system in COUPY **1b** as a consequence of removing the EDG at the 7-position led to a clear blue-shift in the absorption and emission maxima and reduced significantly its fluorescence emission. In parallel, the spectroscopic properties of the conjugates could be also tuned depending on the COUPY fluorophore attached to the Ir(III) core. For instance, attachment of julolidine-fused COUPY coumarin **1c** to the Ir(III) complex red-shifted the absorption maximum of the resultant conjugate **3c** compared to **3a**, which contains the parent COUPY dye **1a**. In contrast, **3b** lost its fluorescence due to the absence of EDG in the coumarin moiety **1b**. This reveals the feasibility of these molecules as theragnostic agents, which can be tuned to act in different regions of the visible spectra depending on minimal structural modifications that do not considerably alter the overall molecular size. In fact, owing to a strong luminescence, Ir(III)-COUPY conjugates could be easily tracked inside cancer cells by confocal microscopy. These experiments revealed that the compounds are highly biocompatible, showing excellent cellular uptake and forming fluorescent vesicles in the cytosol (Figure 4b). It is worth noting that the julolidine-fused system also increased cell accumulation of the resulting conjugate **3c** (Figure 4a), which adds emphasis on how the COUPY scaffold can act as a handle to modulate not only key photophysical properties (e.g., absorption and emission within the phototherapeutic window) but also key biological parameters such as cell entry and accumulation. Another relevant aspect was that COUPY fluorophores and their Ir(III) conjugates meet an important requirement for being considered as promising PDT agents: their minimal toxicity in the dark (Tables S3 and S5). Only coumarin **1c** exhibited dark cytotoxicity in cancer cells, but its correspondent conjugate (**3c**) was found to be inactive, thus revealing that conjugation can be used to diminish undesired dark toxicity of PSs based on cyclometalated Ir(III) complexes or their derivatives (Tables 1 and S3).

The prospect of using Ir(III)-COUPY photosensitizers was very attractive since conjugation of COUPY fluorophore to the metal complex increased by a factor of 10 the ^1^O_2_ quantum yield of all the resulting conjugates compared with the corresponding free coumarins upon excitation at 532 nm (Tables S1 and S2). Compared to the other conjugates, **3a** and its spacer-containing derivatives **3d** and **3e** had the largest ^1^O_2_ quantum yields, thereby confirming their capacity to sensitize highly toxic singlet oxygen after visible light irradiation in non-aqueous environments, independent of the length and rigidity of the spacer linking both moieties. In addition, all Ir(III)-COUPY conjugates were able to promote superoxide (O_2_^•–^) generation in PBS through type-I PDT reactions (Figures 3 and S3). Particularly, the julolidine-fused system-containing conjugate (**3c**) photogenerated O_2_^•–^ with the fastest rates, thereby demonstrating the importance of incorporating a strong EDG in the coumarin scaffold, which not only red-shifts absorption and emission maxima but also enhances the production of superoxide anion radicals. Surprisingly, we also observed that **3b** rapidly generated O_2_^•–^ after visible light irradiation, indicating that the amino group at the 7-position of the coumarin moiety is not strictly necessary to trigger superoxide generation. The generation of O_2_^•–^ was also corroborated by EPR measurements using the DMPO spin trap.

As anticipated, Ir(III)-COUPY conjugates with higher quantum yields for ^1^O_2_ sensitization gave the largest PI values in the cancer cell lines studied herein. This preliminary SARS rationale enabled us to identify **3a**, **3d,** and **3e** as best hit candidates for green light-mediated anticancer therapy. Interestingly, we detected that A2780cis cells were strongly inhibited by light-driven treatments, suggesting that in vitro acquired resistance mechanisms to cisplatin could be tackled by Ir(III)-COUPY-based photoactive therapy.

Local hypoxia within deep-seated tumors is a serious impediment for anticancer PDT.^61^ Given that Ir(III)-COUPY PSs possess the ability to generate both O_2_^–•^ and ^1^O_2_ depending on their microenvironment, which would overcome the limitation of traditional Type II PDT agents under low oxygen environments, we tested their photoactivity under hypoxic conditions. In general, hypoxia decreased the anticancer activity of the conjugates, which confirmed ROS-generating PDT reactions as a source of photocytotoxicity (Figure 4d). In particular, **3a** photocytotoxicity was greatly reduced under a low oxygen environment. However, the incorporation of a spacer between the coumarin moiety and the Ir(III) complex enhanced the phototoxic activity under hypoxia, rendering higher PI values for **3d** and **3e** compared to the parent Ir(III)-COUPY conjugate **3a**. These findings suggest that the addition of rigid or flexible linkers between the metal- and the organic-based scaffold can be used to improve the phototherapeutic profile of these PSs, not only increasing the PI under challenging hypoxia condition but also reducing undesired in vitro dark toxicity (Figure 4d and Table S5).

Besides, we further verified the potential of these PSs by measuring intracellular ROS levels in A2780cis cells. In agreement with ^1^O_2_ and PI values, conjugate **3a** along with **3d** and **3e** produced higher ROS photogeneration than other compounds even under low oxygen conditions (Figure 5). It is worth noting that **3c** also raised ROS levels that were comparable to those produced by **3a, 3d,** and **3e**. However, given that the intracellular amount of **3c** was considerably higher than that found for the other conjugates, it could be reasoned that **3c** is less efficient as PS because a higher amount of compound is required to catalytically photogenerate similar levels of ROS within cancer cells. Overall, the massive build-up of ROS after light irradiation would induce irreversible oxidative damage, triggering necrotic cell death as a result (Figure 5b). Although this cell death mode might be dependent on the PDT dose applied, i.e., PS concentration and light fluence, the fact that necrosis was observed following PDT treatments with these Ir(III)-COUPY conjugates is interesting for two reasons. First, during necrosis, cytosolic components are released into the extracellular space, which may elicit acute inflammation and potentiate an antitumor immune response.^62^ Second, PDT-induced necrosis could be of particular interest for the treatment of apoptosis-refractory cancers such as cisplatin-resistant ovarian cancers. Furthermore, the obtained results using 3D cell culture models confirmed that this type of PSs exhibits potent anticancer PDT activity under hypoxic conditions, as conjugates **3a**, **3d,** and **3e** inhibit tumor growth of MCTS (Figure 5c), which contain hypoxic cellular population in the core of the tumorspheres.

In conclusion, we have synthesized four new PDT agents based on the conjugation between far-red/NIR emitting COUPY fluorophores and a highly potent cyclometalated Ir(III) complex, with the aim of finding rational relationships between chemical structure and biological activity. The structural modifications introduced within the coumarin skeleton clearly influenced the photophysical and biological properties of the resulting Ir(III)-COUPY PSs. On the one hand, the conjugates exhibited appealing features for PDT therapy, including emission in the phototherapeutic window, excellent cellular uptake, no dark cytotoxicity, and high photoinduced toxicity under green-light irradiation, which lead to excellent PI values in several cancer cell lines. On the other hand, the photoinduced generation of different types of ROS (Type II ^1^O_2_ and Type I O_2_^•–^) for all of the conjugates was demonstrated through spectroscopic methods, which would facilitate overcoming the limitations of conventional PSs under low oxygen environments. Remarkably, ROS generation was also confirmed in cisplatin-resistant A2780cis cancer cells for all the Ir(III)-COUPY conjugates, in both normoxia and hypoxia. Overall, three hit conjugates (**3a**, **3d,** and **3e**) containing the same COUPY fluorophore were identified in this SAR study as promising PSs owing to their excellent phototoxicity in A2780cis cells in normoxia upon green light irradiation (PI = 357.1 for **3a**, 227.3 for **3d**, and 268.8 for **3e**). Among them, the linker-containing derivatives **3d** and **3e** are particularly interesting since they exhibited an enhanced phototoxic activity under hypoxic conditions compared with the parent Ir(III)-COUPY conjugate **3a** (PI = 65.8 for **3a**, 131.6 for **3d**, and 147.1 for **3e**), as well as reduced undesired in vitro dark toxicity. Importantly, the massive ROS overproduction during PDT treatments induced necrotic cell death and effectively blocked tumor growth in clinically relevant 3D tumoral models. The high anticancer activity under normoxia and hypoxia conditions and the non-cytotoxicity in the dark, together with the excellent and tunable photophysical and photochemical properties of the Ir(III)-COUPY conjugates, make them useful tools in PDT to deliver efficient ROS-mediated anticancer activity toward chemo-resistant cancer cells using low doses of clinically-relevant green light activation.

## Experimental Section

### Materials and Methods

Unless otherwise stated, common chemicals and solvents (HPLC grade or reagent grade quality) were purchased from commercial sources and used without further purification. Aluminium plates coated with a 0.2 mm thick layer of silica gel 60 F_254_ were used for thin-layer chromatography analyses (TLC), whereas flash column chromatography purification was carried out using silica gel 60 (230–400 mesh). NMR spectra were recorded at 25 °C in a 400 MHz spectrometer using the deuterated solvent as an internal deuterium lock. The residual protic signal of CHCl_3_ and DMSO was used as a reference in ^1^H and ^13^C NMR spectra recorded in CDCl_3_ and DMSO-*d*_6_, respectively. Chemical shifts are reported in part per million (ppm) in the δ scale, coupling constants in Hz, and multiplicity as follows: s (singlet), d (doublet), t (triplet), q (quartet), qt (quintuplet), m (multiplet), dd (doublet of doublets), dt (doublet of triplets), td (triplet of doublets), br (broad signal), and so forth. Electrospray ionization mass spectra (ESI-MS) were recorded on an instrument equipped with single quadrupole detector coupled to an HPLC and high-resolution (HR) ESI-MS on an LC/MS-TOF instrument. The purity of final compounds was determined by reversed-phase high performance liquid chromatography (HPLC) analyses on a Jupiter Proteo C12 column (250 × 4.6 mm, 90 Å, 4 μm, flow rate: 1 mL/min) using linear gradients of 0.1% formic acid in Milli-Q H_2_O (A) and 0.1% formic acid in ACN (B). The HPLC column was maintained at 25 °C. All final compounds were >95% pure by this method.

### Synthesis of COUPY **1b**

#### Synthesis of Compound **4**

Phenol (10 g, 106 mmol) and ethyl acetoacetate (13.6 mL, 106 mmol) were mixed and heated to 100 °C in nitrobenzene (10 mL). In a separate flask, AlCl_3_ (28.1 g, 213 mmol) was dissolved in nitrobenzene (100 mL) at 0 °C—the AlCl_3_ was added in six portions and left to stir at room temperature for 15–30 min. Then, the AlCl_3_ solution was decanted from excess and added dropwise to the phenol and ethyl acetoacetate solution over 45 min. Once the addition was completed, the temperature was increased to 125 °C and the reaction mixture was stirred under reflux for 3 h. The reaction was then cooled to 0 °C and a 1:1 (v/v) mixture of HCl/H_2_O (100 mL) was added. The mixture was then filtrated, and the solid was washed with ethyl acetate. The filtrate was transferred to a separating flask where the aqueous and organic layers were separated: the organic layer was dried over anhydrous Na_2_SO_4_ and filtered. The ethyl acetate was removed by evaporation under reduced pressure and nitrobenzene by distillation in vacuo. The crude product was then purified by column chromatography (silica gel, 0–100% DCM in hexanes) to give 3.79 g (22%) of a brown solid. TLC: *R*_f_ (DCM) 0.34. ^1^H NMR (400 MHz, CDCl_3_): δ (ppm) 7.61 (1H, dd, *J* = 8.0, 1.6 Hz), 7.54 (1H, m), 7.32 (2H, m), 6.30 (1H, s), 2.45 (3H, s). ^13^C{^1^H} NMR (101 Hz, CDCl_3_): δ (ppm) 160.9, 153.6, 152.5, 131.9, 124.7, 124.3, 120.1, 117.2, 115.2, 18.8. LRMS (ESI-TOF) *m*/*z*: [M + H]^+^ calcd for C_10_H_9_O_2_, 161.06; found, 160.83.

#### Synthesis of Compound **5**

Coumarin **4** (4.48 g, 28.0 mmol) was mixed with Lawesson’s Reagent (5.66 g, 14.0 mmol) in toluene (150 mL) and refluxed for 19 h at 105 °C. The crude was then evaporated under reduced pressure and purified by column chromatography (silica gel, 0–50% DCM in hexanes) to obtain 3.80 g of a yellow solid (77% yield). TLC: *R*_f_ (75:25, DCM/hexanes) 0.88. ^1^H NMR (400 MHz, CDCl_3_): δ (ppm) 7.65 (1H, dd, *J* = 8.0, 1.6 Hz), 7.59 (1H, m), 7.49 (1H, m), 7.36 (1H, m), 7.18 (1H, m), 2.38 (3H, s). ^13^C{^1^H} NMR (101 Hz, CDCl_3_): δ (ppm) 197.6, 156.2, 144.3, 132.2, 129.1, 125.5, 124.6, 121.6, 117.1, 18.1. HRMS (ESI-TOF) *m*/*z*: [M + H]^+^ calcd for C_10_H_9_OS, 177.0369: found, 177.0369.

#### Synthesis of Compound **6**

4-Pyridylacetonitrile hydrochloride (1.71 g, 11.08 mmol) and NaH (3.41 g of a 60% dispersion in mineral oil, 85.2 mmol) were dried together in a desiccator alongside compound **5** (1.50 g, 8.52 mmol) in a separate flask. Both were then dissolved in anhydrous ACN (90 and 30 mL, respectively) and the first solution passed to the second and the resulting mixture stirred for 2.5 h at room temperature and under an argon atmosphere. Afterward, AgNO_3_ (3.18 g, 18.74 mmol) was added and the mixture stirred for 2 h under Ar atmosphere and protected from light. The crude product was then evaporated under reduced pressure and purified by column chromatography (silica gel, 50–100% DCM in hexanes first, then 0–0.65% MeOH in DCM) to give 913 mg (41% yield) of a brown solid. TLC: *R*_f_ (DCM/AcOEt 1:1) 0.48. ^1^H NMR (400 MHz, CDCl_3_): δ (ppm) 8.61 (2H, d, *J* = 5.8 Hz), 7.75 (2H, d, *J* = 5.8 Hz), 7.51 (2H, m), 7.32 (2H, m), 7.04 (1H, s), 2.42 (3H, s). ^13^C{^1^H} NMR (101 Hz, CDCl_3_): δ (ppm) 161.9, 152.1, 150.1, 142.8, 140.1, 131.9, 125.3, 124.6, 121.3, 121.2, 118.9, 118.0, 116.4, 84.8, 18.7. HRMS (ESI-TOF) *m*/*z*: [M + H]^+^ calcd for C_17_H_13_N_2_O, 261.1022, found, 261.1026.

#### Synthesis of Compound **1b**

Methyl trifluoromethanesulfonate (16 μL, 0.14 mmol) was added to a solution of coumarin **6** (18.1 mg, 0.070 mmol) in DCM (10 mL) under an argon atmosphere. The mixture was stirred overnight at room temperature and protected from light. The reaction mixture was evaporated under reduced pressure and purified by column chromatography (silica gel, 0–10% MeOH in DCM) to give 16.1 mg of a yellow solid (yield 55%). TLC: *R*_f_ (10% MeOH in DCM) 0.34. ^1^H NMR (400 MHz, DMSO-*d*_6_): δ (ppm) 8.73 (2H, d, *J* = 8.0 Hz), 8.32 (2H, d, *J* = 7.6 Hz), 7.92 (2H, m), 7.81 (1H, m), 7.56 (1H, m), 7.30 (1H, d, *J* = 1.6 Hz), 4.27 (3H, s), 2.62 (3H, d, *J* = 1.2 Hz). ^13^C{^1^H} NMR (101 Hz, DMSO-*d*_6_): δ (ppm) 165.9, 151.6, 150.3, 147.9, 144.5, 133.3, 126.5, 125.6, 122.3, 120.8, 117.4, 117.1, 116.8, 81.3, 46.6, 18.5. HRMS (ESI-TOF) *m*/*z*: [M]^+^ calcd for C_18_H_15_N_2_O, 275.1179; found, 275.1179.

### Synthesis of Ir(III)-COUPY Conjugate **3b**

#### Synthesis of Compound **7**

A solution of methyl bromoacetate (0.9 mL, 9.25 mmol) and coumarin **6** (481 mg, 1.85 mmol) in a 2.5:1 (v/v) mixture of AcOEt and DCM (35 mL) was stirred under reflux at 60 °C for 48 h. The reaction mixture was then evaporated under reduced pressure and purified by column chromatography (silica gel, 0–6.5% MeOH in DCM) to give 623 mg (82% yield) of an orange solid. TLC: *R*_f_ (10% MeOH in DCM) 0.37. ^1^H NMR (400 MHz, DMSO-*d*_6_): δ (ppm) 8.76 (2H, d, *J* = 7.4 Hz), 8.41 (2H, d, *J* = 7.4 Hz), 8.04 (1H, d, *J* = 7.6 Hz), 7.95 (1H, dd, *J* = 8.0, 1.6 Hz), 7.83 (1H, td, *J* = 8.0, 1.6 Hz), 7.58 (1H, m), 7.35 (1H, s), 5.60 (2H, s), 3.79 (3H, s), 2.65 (3H, s). ^13^C{^1^H} NMR (101 Hz, DMSO-*d*_6_): δ (ppm) 167.3, 166.5, 151.7, 151.3, 149.3, 144.9, 133.5, 126.6, 125.6, 122.0, 120.8, 117.4, 117.3, 116.9, 81.6, 58.9, 53.0, 18.6. HRMS (ESI-TOF) *m*/*z*: [M]^+^ calcd for C_20_H_17_N_2_O_3_, 333.1234; found, 333.1243.

#### Synthesis of Compound **8**

Compound **7** (214 mg, 0.52 mmol) was refluxed in a solution containing concentrated HCl (43 mL, 520 mmol) and H_2_O (86 mL, Milli-Q quality) at 60 °C for 5.5 h. The crude mixture was then evaporated under reduced pressure to give a red solid, which was used without further purification in the next step. Analytical HPLC (10 to 100% B in 30 min, formic acid additive): Rt = 12.5 min. LRMS (ESI-TOF) *m*/*z*: [M]^+^ calcd for C_19_H_15_N_2_O_3_, 319.11; found, 319.53.

#### Synthesis of Compound **9**

Coumarin **8** (80.4 mg, 0.23 mmol) and HATU (88.8 mg, 0.23 mmol) were dissolved in anhydrous DMF (8 mL) under an Ar atmosphere. After addition of DIPEA (40 μL, 0.23 mmol), the reaction mixture was stirred for 5 min under Ar at room temperature and protected from light. On the other hand, DIPEA (40 μL, 0.23 mmol) was added to a solution of *N*-Boc-1,3-propanediamine hydrochloride (57.3 mg, 0.27 mmol) in anhydrous DMF (5 mL), and the resulting mixture was combined with the coumarin solution. After addition of DIPEA (40 μL, 0.23 mmol), the reaction mixture was stirred for 2 h at room temperature under Ar and protected from light. The crude product was evaporated under reduced pressure and purified by column chromatography (silica gel, 0–12% MeOH in DCM) to give 30.5 mg of a purple solid (yield: 26%). TLC: *R*_f_ (10% MeOH in DCM) 0.26. ^1^H NMR (400 MHz, DMSO-*d*_6_): δ (ppm) 8.69 (2H, d, *J* = 7.4 Hz), 8.52 (1H, t, *J* = 5.6 Hz), 8.36 (2H, d, *J* = 7.4 Hz), 7.98 (1H, dd, *J* = 8.4, 1.2 Hz), 7.94 (1H, dd, *J* = 8.0, 1.2 Hz), 7.82 (1H, td, *J* = 7.2 Hz, *J* = 1.6 Hz), 7.57 (1H, td, *J* = 8.3, 0.8 Hz), 7.33 (1H, d, *J* = 1.2 Hz), 6.82 (1H, t, *J* = 5.6 Hz), 5.33 (2H, s), 3.15 (2H, q, *J* = 6.4 Hz), 2.97 (2H, q, *J* = 6.4 Hz), 2.64 (3H, d, *J* = 1.2 Hz), 1.58 (2H, qt, *J* = 7.0 Hz), 1.37 (9H, s). ^13^C{^1^H} NMR (101 Hz, DMSO-*d*_6_): δ (ppm) 166.3, 164.6, 155.6, 151.7, 150.8, 148.7, 145.0, 133.4, 126.6, 125.7, 121.8, 120.8, 117.4, 116.9, 81.5, 77.5, 60.1, 37.5, 36.9, 29.3, 28.2, 18.6. HRMS (ESI-TOF) *m*/*z*: [M]^+^ calcd for C_27_H_31_N_4_O_4_, 475.2340; found, 475.2336.

#### Synthesis of Compound **10**

A cooled down solution of hydrochloric acid in dioxane (4 M, 3 mL) was added to coumarin **9** (10 mg, 0.021 mmol). The reaction mixture was stirred for 25 min in an ice bath under an Ar atmosphere and protected from light. After removal of the solvent under reduced pressure, several co-evaporations from acetonitrile were carried out. The crude product was used without further purification in the next step since HPLC-MS analysis revealed that the removal of the Boc group was quantitative. Analytical HPLC (10 to 100% B in 30 min, formic acid additive): Rt = 5.5 min. LRMS (ESI-TOF) *m*/*z*: [M]^+^ calcd for C_22_H_23_N_4_O_2_, 375.18; found, 375.19.

#### Synthesis of Ir(III)-COUPY Conjugate **3b**

To a solution of Ir complex **2b** (15.6 mg, 14.6 μmol) and HATU (5.7 mg, 14.6 μmol) in anhydrous DMF (2.5 mL) under an Ar atmosphere, DIPEA (3 μL, 14.6 μmol) was added and the mixture stirred for 10 min under Ar at room temperature and protected from light. After addition of a solution of coumarin **10** (9.8 mg, 21.9 μmol) and DIPEA (15 μL, 73 μmol) in anhydrous DMF (3 mL), the reaction mixture was stirred for 2.5 h at room temperature under Ar and protected from light. After evaporation under reduced pressure, the crude was purified by column chromatography (silica gel, 0–12.5% MeOH in DCM) to give 10.1 mg of a yellow solid (yield: 47%). TLC: *R*_f_ (15% MeOH in DCM) 0.61. ^1^H NMR (400 MHz, DMSO-*d*_6_): δ (ppm) 14.19 (1H, br s), 13.97 (1H, br s), 8.89 (1H, d, *J* = 8.7 Hz), 8.72 (3H, m), 8.35 (2H, d, *J* = 7.4 Hz), 8.28 (1H, d, *J* = 8.8 Hz), 8.15 (1H, d, *J* = 8.4 Hz), 8.09 (1H, d, *J* = 8.8 Hz), 7.95 (2H, m), 7.82 (3H, m), 7.64 (1H, t, *J* = 7.8 Hz), 7.55 (2H, d, m), 7.47 (1H, d, *J* = 8.1 Hz), 7.32 (1H, d, *J* = 1.2 Hz), 7.19 (1H, t, *J* = 8.5 Hz), 7.10 (2H, m), 7.01 (1H, t, *J* = 7.5 Hz), 6.89 (1H, t, *J* = 7.5 Hz), 6.83 (1H, t, *J* = 7.7 Hz), 6.76 (2H, t, *J* = 7.3 Hz), 6.68 (1H, s), 6.31 (1H, d, *J* = 7.3 Hz), 6.19 (1H, d, *J* = 7.3 Hz), 5.97 (1H, d, *J* = 8.3 Hz), 5.71 (1H, d, *J* = 8.3 Hz), 5.40 (2H, s), 5.10 (2H, m), 3.20 (4H, m), 2.63 (3H, d, *J* = 1.1 Hz), 1.69 (4H, m), 1.23 (4H, m), 0.91 (2H, m), 0.65 (3H, t, *J* = 7.2 Hz). ^13^C NMR (101 MHz, DMSO-*d*_6_): δ (ppm) 166.3, 165.8, 164.7, 164.4, 163.8, 155.6, 152.4, 151.7, 150.8, 149.8, 148.7, 148.4, 145.0, 143.3, 141.3, 139.5, 139.5, 139.2, 137.9, 134.2, 134.1, 133.4, 133.0, 132.9, 132.3, 132.1, 132.0, 131.3, 130.4, 130.0, 129.6, 129.4, 128.9, 128.9, 128.8, 126.5, 125.7, 124.7, 124.6, 124.4, 123.2, 123.0, 122.4, 121.8, 121.6, 120.8, 120.6, 117.8, 117.4, 117.3, 116.9, 113.4, 112.9, 112.6, 81.5, 60.2, 56.0, 45.9, 37.1, 37.0, 31.9, 29.0, 19.0, 18.6, 13.4. Analytical HPLC (10 to 100% B in 30 min, formic acid additive): Rt = 12.6 min. HRMS (ESI-TOF) *m*/*z*: [M]^2+^ calcd for C_69_H_58_IrN_11_O_3_, 640.7171; found, 640.7169.

### Synthesis of COUPY **1c**

#### Synthesis of Compound **11**

Coumarin 102 (1.17 g, 4.58 mmol) and Lawesson’s reagent (1.11 g, 2.75 mmol) were dissolved in toluene (30 mL) and heated at 100 °C for 15 h. After evaporation under reduced pressure, the residue was purified by column chromatography (silica gel, 50–70% DCM in hexanes) to give an orange solid (666 mg, 54%): TLC *R*_f_ (DCM) 0.53. ^1^H NMR (400 MHz, CDCl_3_): δ (ppm) 7.03 (1H, s), 6.91 (1H, br q, *J* = 0.8 Hz), 3.29 (4H, q, *J* = 6.8 Hz), 3.00 (2H, t, *J* = 6.4 Hz), 2.79 (2H, t, *J* = 6.4 Hz), 2.27 (3H, d, *J* = 0.8 Hz), 1.98 (4H, m). ^13^C{^1^H} NMR (101 Hz, CDCl_3_): δ (ppm) 196.4, 154.5, 146.5, 146.4, 123.4, 121.8, 120.2, 111.2, 106.1, 50.1, 49.8, 28.0, 21.5, 20.7, 20.5, 18.1; HRMS (ESI-TOF) *m*/*z*: [M + H]^+^ calcd for C_16_H_18_NOS, 272.1104; found, 272.1108.

#### Synthesis of Compound **12**

To a solution of 4-pyridylacetonitrile hydrochloride (256.4 mg, 1.66 mmol) and NaH (60% dispersion in mineral oil, 442.2 mg, 11.06 mmol) in dry ACN (50 mL) under an Ar atmosphere and protected from light, a solution of coumarin **11** (300 mg, 1.11 mmol) in dry ACN (10 mL) was added. After the mixture was stirred for 2 h at room temperature, silver nitrate (413.2 mg, 2.43 mmol) was added, and the reaction mixture was stirred at room temperature for 2 h under an Ar atmosphere and protected from light. The crude product was evaporated under reduced pressure and purified by column chromatography (silica gel, 0–3.5% MeOH in DCM) to give 120.6 mg of an orange solid (yield 31%). TLC: *R*_f_ (10% MeOH in DCM) 0.67. ^1^H NMR (400 MHz, CDCl_3_): δ (ppm) 8.54 (2H, m), 7.69 (2H, m), 6.94 (1H, s), 6.72 (1H, br q, *J* = 0.8 Hz), 3.27 (4H, m), 2.90 (2H, t, *J* = 6.5 Hz), 2.78 (2H, t, *J* = 6.3 Hz), 2.31 (3H, d, *J* = 0.8 Hz), 2.00 (4H, m). ^13^C{^1^H} NMR (101 Hz, CDCl_3_): δ (ppm) 163.2, 149.2, 145.4, 144.0, 140.9, 121.4, 120.5, 119.9, 118.4, 111.5, 109.5, 106.0, 80.3, 49.6, 48.9, 27.3, 21.1, 21.0, 20.5, 18.1. HRMS (ESI-TOF) *m*/*z*: [M + H]^+^ calcd for C_23_H_22_N_3_O, 356.1757; found, 356.1761.

#### Synthesis of Compound **1c**

Methyl trifluoromethanesulfonate (32 μL, 0.28 mmol) was added to a solution of coumarin **12** (49 mg, 0.14 mmol) in DCM (25 mL) under an argon atmosphere. The mixture was stirred overnight at room temperature and protected from light. The reaction mixture was evaporated under reduced pressure and purified by column chromatography (silica gel, 0–6% MeOH in DCM) to give 47 mg of a red solid (yield 66%). TLC: *R*_f_ (10% MeOH in DCM) 0.33. ^1^H NMR (400 MHz, DMSO-*d*_6_): δ (ppm) 8.60 (2H, d, *J* = 7.2 Hz), 7.99 (2H, d, *J* = 7.2 Hz), 7.37 (1H, s), 6.87 (1H, s), 4.13 (3H, s), 3.37 (4H, m), 2.93 (2H, t, *J* = 6.4 Hz), 2.81 (2H, t, *J* = 6.2 Hz), 2.50 (3H, s), 1.96 (2H, m), 1.90 (2H, m). ^13^C{^1^H} NMR (101 Hz, DMSO-*d*_6_): δ (ppm) 166.2, 152.4, 150.4, 148.8, 147.4, 143.5, 122.7, 121.4, 120.1, 118.6, 110.3, 105.0, 77.1, 49.5, 48.7, 45.9, 27.1, 20.8, 20.4, 19.8, 18.5. HRMS (ESI-TOF) *m*/*z*: [M]^+^ calcd for C_24_H_24_N_3_O, 370.1912; found, 370.1914.

### Synthesis of Ir(III)-COUPY Conjugate **3c**

#### Synthesis of Compound **13**

Methyl bromoacetate (52 μL, 0.56 mmol) was added to a solution of coumarin **12** (100 mg, 0.28 mmol) in AcOEt/ACN 1:1 (20 mL). The mixture was stirred overnight at 50 °C under an Ar atmosphere and protected from light. The crude product was evaporated under reduced pressure and purified by column chromatography (silica gel, 0–8% MeOH in DCM) to give 124 mg of a blue solid (yield, 87%). TLC: *R*_f_ (10% MeOH in DCM) 0.40. ^1^H NMR (400 MHz, CDCl_3_): δ (ppm) 8.81 (2H, d, *J* = 6.8 Hz), 8.01 (2H, d, *J* = 6.8 Hz), 7.15 (1H, s), 6.90 (1H, s), 5.80 (2H, s), 3.85 (3H, s), 3.39 (4H, m), 3.05 (2H, t, *J* = 6.4 Hz), 2.84 (2H, t, *J* = 6.4 Hz), 2.49 (3H, s), 2.16 (2H, m), 2.01 (2H, m). ^13^C{^1^H} NMR (101 Hz, CDCl_3_): δ (ppm) 167.3, 167.2, 152.7, 151.5, 150.8, 148.4, 143.5, 122.5, 122.4, 119.8, 118.5, 111.4, 111.0, 106.0, 79.2, 58.7, 53.6, 50.4, 49.8, 28.1, 21.9, 21.0, 20.3, 19.2. HRMS (ESI-TOF) *m*/*z*: [M]^+^ calcd for C_26_H_26_N_3_O_3_, 428.1969; found, 428.1974.

#### Synthesis of Compound **14**

A 1:2 (v/v) mixture of HCl (37%) and Milli-Q water (51 mL) was added to coumarin **13** (98.9 mg, 0.19 mmol). The reaction mixture was stirred for 5 h at 60 °C under an Ar atmosphere and protected from light. After removal of the major part of the solvent, several coevaporations from acetonitrile were carried out. The crude product was used without further purification in the next step. Analytical HPLC (10 to 100% B in 30 min, formic acid additive): Rt = 16.6 min. LRMS (ESI-TOF) *m*/*z*: [M]^+^ calcd for C_25_H_24_N_3_O_3_, 414.18; found, 413.81.

#### Synthesis of Compound **15**

Coumarin **14** (87.5 mg, 0.19 mmol) and HATU (76 mg, 0.19 mmol) were dissolved in anhydrous DMF (8 mL) under an Ar atmosphere. After addition of DIPEA (70 μL, 0.39 mmol), the reaction mixture was stirred for 5 min under Ar at room temperature and protected from light. On the other hand, DIPEA (35 μL, 0.19 mmol) was added to a solution of *N*-Boc-1,3-propanediamine hydrochloride (62 mg, 0.29 mmol) in anhydrous DMF (5 mL), and the resulting mixture was combined with the coumarin solution. After addition of DIPEA (70 μL, 0.39 mmol), the reaction mixture was stirred for 2 h at room temperature under Ar and protected from light. The crude product was evaporated under reduced pressure and purified by column chromatography (silica gel, 0–12.0% MeOH in DCM) to give 29 mg of a purple solid (yield: 24%). TLC: *R*_f_ (10% MeOH in DCM) 0.59. ^1^H NMR (400 MHz, DMSO-*d*_6_): δ (ppm) 8.69 (1H, br s), 8.55 (2H, d, *J* = 6.8 Hz), 7.96 (2H, d, *J* = 6.6 Hz), 7.35 (1H, s), 6.84 (2H, m), 5.19 (2H, s), 3.37 (4H, m), 3.13 (2H, q, *J* = 6.6 Hz), 2.95 (4H, m), 2.80 (2H, m), 2.49 (3H, s), 1.92 (4H, m), 1.57 (2H, quint, *J* = 6.9 Hz), 1.37 (9H, s). ^13^C NMR (101 MHz, DMSO-*d*_6_): δ (ppm) 166.2, 164.8, 155.6, 152.8, 150.5, 149.3, 147.6, 143.9, 122.7, 121.7, 119.3, 118.5, 110.5, 109.8, 104.9, 77.5, 77.5, 59.5, 49.6, 48.7, 40.2, 37.5, 36.8, 31.3, 29.3, 29.0, 28.2, 27.1, 22.1, 20.8, 20.4, 19.8, 18.5, 14.0. HRMS (ESI-TOF) *m*/*z*: [M]^+^ calcd for C_33_H_40_N_5_O_4_, 570.3075; found, 570.3079.

#### Synthesis of Compound **16**

A cooled down solution of hydrochloric acid in dioxane (4 M, 8 mL) was added to coumarin **15** (19 mg, 0.031 mmol). The reaction mixture was stirred for 25 min in an ice bath under an Ar atmosphere and protected from light. After removal of the solvent, several co-evaporations from acetonitrile were carried out. The crude product was used without further purification since HPLC-MS analysis revealed that the removal of the Boc group was quantitative. Analytical HPLC (10 to 100% B in 30 min, formic acid additive): Rt = 10.6 min. LRMS (ESI-TOF) *m*/*z*: [M]^+^ calcd for C_28_H_32_N_5_O_2_, 470.26; found, 470.11.

#### Synthesis of Ir(III)-COUPY Conjugate **3c**

To a solution of Ir complex **2b** (30 mg, 28.1 μmol) and HATU (11.0 mg, 28.1 μmol) in anhydrous DMF (5 mL) under an Ar atmosphere, DIPEA (10 μL, 56.1 μmol) was added and the mixture stirred for 10 min under Ar at room temperature and protected from light. After addition of a solution of coumarin **16** (16.9 mg, 31.2 μmol) and DIPEA (25 μL, 140.3 μmol) in anhydrous DMF (6 mL), the reaction mixture was stirred for 2.5 h at room temperature under Ar and protected from light. After evaporation under reduced pressure, the crude was purified by column chromatography (silica gel, 0–12% MeOH in DCM) to give 14.5 mg of a dark blue solid (yield: 33%). TLC: *R*_f_ (10% MeOH in DCM) 0.47. ^1^H NMR (400 MHz, DMSO-*d*_6_): δ (ppm) 13.99 (2H, br s), 8.88 (1H, d, *J* = 8.8 Hz), 8.69 (1H, d, *J* = 9.0 Hz), 8.66 (1H, m), 8.55 (2H, d, *J* = 7.0 Hz), 8.30 (1H, d, *J* = 9.0 Hz), 8.15 (1H, d, *J* = 8.3 Hz), 8.08 (1H, d, *J* = 8.9 Hz), 7.99 (2H, d, *J* = 7.2 Hz), 7.93 (1H, d, *J* = 7.7 Hz), 7.83 (2H, m), 7.72 (1H, m), 7.63 (1H, t, *J* = 7.8 Hz), 7.51 (1H, d, *J* = 8.1 Hz), 7.45 (1H, d, *J* = 8.1 Hz), 7.40 (1H, s), 7.19 (1H, t, *J* = 8.7 Hz), 7.09 (3H, m), 7.00 (1H, t, *J* = 7.4 Hz), 6.90 (2H, m), 6.81 (1H, t, *J* = 7.5 Hz), 6.73 (2H, m), 6.68 (1H, s), 6.30 (1H, d, *J* = 7.6 Hz), 6.19 (1H, d, *J* = 7.6 Hz), 5.95 (1H, d, *J* = 8.2 Hz), 5.70 (1H, d, *J* = 8.2 Hz), 5.21 (2H, s), 5.10 (2H, m), 3.19 (4H, sext, *J* = 7.2 Hz), 2.92 (2H, t, *J* = 6.1 Hz), 2.81 (2H, t, *J* = 6.0 Hz), 1.91 (4H, m), 1.68 (5H, m), 1.23 (4H, m), 0.94 (4H, m), 0.66 (3H, t, *J* = 7.2 Hz). ^13^C NMR (101 MHz, DMSO-*d*_6_): δ (ppm) 165.8, 164.9, 164.0, 155.6, 152.9, 152.4, 150.6, 149.8, 149.4, 148.5, 147.6, 143.9, 141.2, 139.5, 137.9, 132.4, 132.1, 132.0, 131.2, 130.2, 129.9, 129.3, 129.0, 128.9, 128.7, 124.7, 124.5, 124.2, 124.2, 123.0, 122.9, 122.8, 122.7, 122.3, 121.7, 121.6, 120.5, 119.4, 118.5, 117.7, 113.4, 113.3, 113.1, 113.0, 112.8, 112.6, 110.6, 105.0, 77.4, 67.5, 59.5, 49.6, 48.7, 45.9, 37.0, 31.9, 29.0, 27.1, 20.8, 20.4, 19.8, 19.0, 18.8, 18.5, 13.4. HRMS (ESI-TOF) *m*/*z*: [M]^2+^ calcd for C_75_H_67_IrN_12_O_3_, 688.2539; found, 688.2545. Analytical HPLC (10 to 100% B in 30 min, formic acid additive): Rt = 15.5 min.

### Synthesis of Ir(III)-COUPY Conjugate **3d**

#### Synthesis of Compound **18**

To a solution of Boc-6-aminohexanoic acid (42.8 mg, 0.19 mmol) and HATU (72.5 mg, 0.19 mmol) in anhydrous DMF (6 mL) under an Ar atmosphere, DIPEA (65 μL, 0.37 mmol) was added and the mixture stirred for 10 min under Ar at room temperature and protected from light. After addition of a solution of coumarin **17** (32 mg, 0.062 mmol) and DIPEA (54 μL, 0.31 mmol) in anhydrous DMF (6 mL), the reaction mixture was stirred for 2.5 h at room temperature under Ar and protected from light. After evaporation under reduced pressure, the crude was purified by column chromatography (silica gel, 0–12% MeOH in DCM) to give 19.7 mg of a pink solid (yield: 49%). TLC: *R*_f_ (10% MeOH in DCM) 0.43. ^1^H NMR (400 MHz, DMSO-*d*_6_): δ (ppm) 8.59 (1H, br s), 8.53 (2H, d, *J* = 6.2 Hz), 8.16 (2H, d, *J* = 6.4 Hz), 7.81 (1H, br s), 7.72 (1H, d, *J* = 9.0 Hz), 6.99 (3H, m), 6.74 (1H, br s), 5.24 (2H, s), 3.55 (4H, m), 3.10 (4H, m), 2.86 (2H, m), 2.55 (3H, s), 2.03 (2H, m), 1.58 (2H, m), 1.46 (2H, m), 1.36 (11H, m), 1.18 (8H, m). ^13^C NMR (101 MHz, DMSO-*d*_6_): δ (ppm) 172.1, 166.8, 164.8, 155.6, 154.9, 152.8, 152.0, 149.2, 144.2, 127.0, 120.1, 118.2, 111.9, 110.5, 110.4, 96.4, 78.1, 77.3, 59.6, 44.2, 36.9, 36.1, 35.4, 29.3, 29.0, 28.3, 26.0, 25.1, 18.4, 12.4. HRMS (ESI-TOF) *m*/*z*: [M]^+^ calcd for C_37_H_51_N_6_O_5_, 659.3915; found, 659.3928.

#### Synthesis of Compound **20**

A cooled down solution of hydrochloric acid in dioxane (4 M, 6 mL) was added to coumarin **18** (16.1 mg, 0.023 mmol). The reaction mixture was stirred for 20 min in an ice bath under an Ar atmosphere and protected from light. After removal of the solvent, several co-evaporations from acetonitrile were carried out. The crude product was used without further purification since HPLC-MS analysis revealed that the removal of the Boc group was quantitative. Analytical HPLC (10 to 100% B in 30 min, formic acid additive): Rt = 8.5 min. LRMS (ESI-TOF) *m*/*z*: [M]^+^ calcd for C_32_H_43_N_6_O_3_, 559.34; found, 559.49.

#### Synthesis of Ir(III)-COUPY Conjugate **3d**

To a solution of Ir complex **2b** (20.6 mg, 19.3 μmol) and HATU (7.4 mg, 19.3 μmol) in anhydrous DMF (4 mL) under an Ar atmosphere, DIPEA (7 μL, 38.6 μmol) was added and the mixture stirred for 10 min under Ar at room temperature and protected from light. After addition of a solution of coumarin **20** (14.6 mg, 23.2 μmol) and DIPEA (17 μL, 96.4 μmol) in anhydrous DMF (5 mL), the reaction mixture was stirred for 2.5 h at room temperature under Ar and protected from light. After evaporation under reduced pressure, the crude was purified by column chromatography (silica gel, 0–12% MeOH in DCM) to give 27.9 mg of a purple solid (yield: 68%). ^1^H NMR (400 MHz, DMSO-*d*_6_): δ (ppm) 14.16 (2H, br s), 8.88 (1H, d, *J* = 8.7 Hz), 8.69 (1H, d, *J* = 8.9 Hz), 8.65 (1H, t, *J* = 5.5 Hz), 8.53 (2H, d, *J* = 7.5 Hz), 8.29 (1H, d, *J* = 9.0 Hz), 8.15 (3H, m), 8.07 (1H, d, *J* = 8.9 Hz), 7.96 (1H, d, *J* = 7.5 Hz), 7.92 (1H, t, *J* = 5.6 Hz), 7.87 (1H, d, *J* = 7.6 Hz), 7.83 (1H, dd, *J* = 8.8 Hz, *J* = 1.5 Hz), 7.70 (1H, d, *J* = 9.2 Hz), 7.63 (1H, t, *J* = 8.0 Hz), 7.53 (2H, m), 7.46 (1H, d, *J* = 8.1 Hz), 7.19 (1H, t, *J* = 8.8 Hz), 7.00 (8H, m), 6.81 (1H, td, *J* = 7.6 Hz, *J* = 1.3 Hz), 6.74 (2H, m), 6.64 (1H, d, *J* = 1.1 Hz), 6.30 (1H, d, *J* = 7.3 Hz), 6.19 (1H, d, *J* = 7.4 Hz), 5.95 (1H, d, *J* = 8.3 Hz), 5.70 (1H, d, *J* = 8.2 Hz), 5.25 (2H, m), 5.09 (2H, m), 3.53 (4H, q, *J* = 6.9 Hz), 3.11 (6H, m), 2.53 (2H, d, *J* = 0.8 Hz), 2.09 (2H, t, *J* = 7.4 Hz), 1.71 (2H, m), 1.51 (6H, m), 1.25 (3H, m), 1.15 (6H, t, *J* = 7.0 Hz), 0.99 (2H, m), 0.65 (3H, t, *J* = 7.2 Hz). ^13^C NMR (101 MHz, DMSO-*d*_6_): δ (ppm) 172.1, 166.8, 165.5, 164.8, 164.6, 164.0, 155.6, 154.9, 152.8, 152.4, 152.0, 149.8, 149.2, 148.5, 144.2, 143.4, 141.2, 139.6, 139.5, 139.3, 137.8, 134.5, 134.3, 132.4, 132.1, 132.0, 131.2, 130.2, 129.8, 129.0, 128.8, 128.8, 127.0, 124.8, 124.5, 124.3, 123.0, 123.0, 122.2, 121.6, 120.5, 120.1, 118.2, 117.5, 113.3, 113.1, 112.8, 112.6, 111.9, 110.5, 110.4, 96.4, 78.1, 59.6, 45.9, 44.2, 36.9, 36.2, 35.4, 31.9, 29.0, 26.2, 25.1, 19.0, 18.4, 13.4, 12.4. HRMS (ESI-TOF) *m*/*z*: [M]^2+^ calcd for C_79_H_78_IrN_13_O_4_, 732.7959; found, 732.7973. Analytical HPLC (10 to 100% B in 30 min, formic acid additive): Rt = 13.7 min.

### Synthesis of Ir(III)-COUPY Conjugate **3e**

#### Synthesis of Compound **19**

To a solution of *trans*-4-*N*-Boc-aminomethyl-cyclohexanecarboxylic acid (56 mg, 0.22 mmol) and HATU (85.3 mg, 0.22 mmol) in anhydrous DMF (6 mL) under an Ar atmosphere, DIPEA (77 μL, 0.44 mmol) was added and the mixture stirred for 10 min under Ar at room temperature and protected from light. After addition of a solution of coumarin **17** (37.6 mg, 0.073 mmol) and DIPEA (64 μL, 0.36 mmol) in anhydrous DMF (6 mL), the reaction mixture was stirred for 2.5 h at room temperature under Ar and protected from light. After evaporation under reduced pressure, the crude was purified by column chromatography (silica gel, 0–11% MeOH in DCM) to give 35.4 mg of a pink solid (yield: 68%). TLC: *R*_f_ (10% MeOH in DCM) 0.38. ^1^H NMR (400 MHz, DMSO-*d*_6_): δ (ppm) 8.60 (1H, m), 8.53 (2H, d, *J* = 7.2 Hz), 8.16 (2H, d, *J* = 7.2 Hz), 7.73 (1H, m), 7.72 (1H, d, *J* = 9.2 Hz), 7.02 (1H, m), 6.98 (1H, dd, *J* = 9.2, 2.4 Hz), 6.93 (1H, s), 6.78 (1H, t, *J* = 6.0 Hz), 5.24 (2H, s), 3.56 (4H, q, *J* = 7.2 Hz), 3.13 (2H, q, *J* = 6.4 Hz), 3.07 (2H, q, *J* = 6.0 Hz), 2.75 (2H, t, *J* = 6.4 Hz), 2.55 (3H, s), 2.00 (1H, m), 1.68 (4H, m), 1.57 (2H, qt, *J* = 6.8 Hz), 1.36 (9H, s), 1.26 (4H, m), 1.18 (6H, t, *J* = 7.2 Hz), 0.84 (2H, m). ^13^C{^1^H} NMR (101 Hz, DMSO-*d*_6_): δ (ppm) 175.2, 166.8, 164.8, 155.7, 154.9, 152.8, 152.0, 149.2, 144.2, 127.0, 120.1, 118.2, 111.9, 110.5, 110.4, 96.4, 78.1, 77.3, 59.6, 46.1, 44.2, 44.1, 37.4, 36.8, 36.0, 29.5, 29.0, 28.8, 28.3, 18.4, 12.4. HRMS (ESI-TOF) *m*/*z*: [M]^+^ calcd for C_39_H_53_N_6_O_5_, 685.4072; found, 685.4072.

#### Synthesis of Compound **21**

A cooled down solution of hydrochloric acid in dioxane (4 M, 11 mL) was added to coumarin **19** (28 mg, 0.039 mmol). The reaction mixture was stirred for 20 min in an ice bath under an Ar atmosphere and protected from light. After removal of the solvent, several co-evaporations from acetonitrile were carried out. The crude product was used without further purification since HPLC-MS analysis revealed that the removal of the Boc group was quantitative. Analytical HPLC (10 to 100% B in 30 min, formic acid additive): Rt = 8.6 min. LRMS (ESI-TOF) *m*/*z*: [M]^+^ calcd for C_34_H_45_N_6_O_3_, 585.35; found, 585.31.

#### Synthesis of Ir(III)-COUPY Conjugate **3e**

To a solution of Ir complex **2b** (34.6 mg, 32.3 μmol) and HATU (12.7 mg, 32.3 μmol) in anhydrous DMF (5 mL) under an Ar atmosphere, DIPEA (12 μL, 64.7 μmol) was added and the mixture stirred for 10 min under Ar at room temperature and protected from light. After addition of a solution of coumarin **21** (25.5 mg, 38.8 μmol) and DIPEA (29 μL, 161.7 μmol) in anhydrous DMF (6 mL), the reaction mixture was stirred for 2.5 h at room temperature under Ar and protected from light. After evaporation under reduced pressure, the crude was purified by column chromatography (silica gel, 0–12% MeOH in DCM) to give 34.7 mg of a purple solid (yield: 58%). ^1^H NMR (400 MHz, DMSO-*d*_6_): δ (ppm) 14.40 (2H, br s), 8.89 (1H, d, *J* = 8.8 Hz), 8.70 (2H, m), 8.54 (2H, d, *J* = 7.5 Hz), 8.28 (1H, d, *J* = 9.0 Hz), 8.15 (3H, m), 8.07 (1H, d, *J* = 8.9 Hz), 7.97 (1H, d, *J* = 6.9 Hz), 7.91 (1H, d, *J* = 7.6 Hz), 7.82 (2H, m), 7.70 (1H, d, *J* = 9.2 Hz), 7.62 (2H, m), 7.52 (1H, d, *J* = 8.1 Hz), 7.46 (1H, d, *J* = 8.1 Hz), 7.19 (1H, t, *J* = 8.8 Hz), 7.12 (2H, q, *J* = 7.9 Hz), 6.95 (6H, m), 6.80 (1H, t, *J* = 7.6 Hz), 6.75 (2H, t, *J* = 7.4 Hz), 6.63 (1H, s), 6.30 (1H, d, *J* = 7.2 Hz), 6.19 (1H, d, *J* = 7.4 Hz), 5.96 (1H, d, *J* = 8.3 Hz), 5.73 (1H, d, *J* = 8.2 Hz), 5.26 (2H, s), 5.10 (2H, m), 3.54 (4H, q, *J* = 6.9 Hz), 3.07 (7H, m), 2.53 (2H, s), 2.07 (1H, m), 1.73 (6H, m), 1.58 (2H, qt, *J* = 6.9 Hz), 1.35 (3H, m), 1.16 (6H, t, *J* = 7.0 Hz), 0.92 (4H, m), 0.63 (3H, t, *J* = 7.2 Hz). ^13^C NMR (101 MHz, DMSO-*d*_6_): δ (ppm) 175.2, 166.8, 165.8, 164.8, 164.5, 163.8, 155.6, 154.9, 152.8, 152.3, 152.0, 149.8, 149.2, 148.5, 144.2, 143.4, 141.3, 139.5, 139.2, 137.8, 134.5, 134.3, 134.2, 133.2, 133.1, 132.3, 132.3, 132.0, 131.2, 130.3, 129.9, 129.4, 128.9, 128.9, 128.8, 127.6, 127.0, 124.8, 124.6, 124.3, 123.1, 123.0, 122.9, 122.2, 121.6, 120.6, 120.1, 119.3, 118.2, 117.5, 113.3, 113.0, 112.9, 112.5, 111.9, 110.5, 110.4, 96.5, 78.1, 59.6, 45.9, 45.3, 44.2, 44.1, 37.1, 36.9, 36.0, 31.9, 29.7, 29.0, 28.9, 19.0, 18.4, 13.4, 12.4. HRMS (ESI-TOF) *m*/*z*: [M]^2+^ calcd for C_81_H_80_IrN_13_O_4_, 745.8037; found, 745.8047. Analytical HPLC (10 to 100% B in 30 min, formic acid additive): Rt = 15.8 min.

### Photophysical and Photochemical Characterization

For photophysical measurements, all solvents used were spectroscopic grade. Absorption spectra were recorded in a Varian Cary 500 UV/vis/NIR or Varian Cary 6000i spectrophotometer at room temperature. Molar absorption coefficients (ε) were determined by direct application of the Beer–Lambert law, using solutions of the compounds in each solvent with concentrations ranging from 10^–6^ to 10^–5^ M. Emission spectra were registered in a Photon Technology International (PTI) fluorimeter or in a Horiba Fluoromax-4 spectrofluorometer. Fluorescence quantum yields (Φ_F_) were measured by the comparative method using cresyl violet in ethanol (CV; Φ_F;ref_ = 0.54 ± 0.03) as reference.^63^ Then, optically matched solutions of the compounds and CV were excited and the fluorescence spectra were recorded. The absorbance of sample and reference solutions was set below 0.1 at the excitation wavelength, and Φ_F_ was calculated using the following eq 1
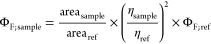
1where area_sample_ and area_ref_ are the integrated fluorescence for the sample and the reference and η_sample_ and η_ref_ are the refractive index of sample and reference solutions respectively. The uncertainty in the experimental value of Φ_F_ has been estimated to be approximately 10%.

Phosphorescence quantum yields (Φ_P_) of the iridium complex were determined analogously to Φ_F_ using argon-saturated meso-tetra-5,10,15,20-phenylporphine as reference (Φ_F_ = 0.11) in toluene.^64^

Singlet oxygen generation was studied by time-resolved near-infrared phosphorescence by means of a customized setup. Briefly, a pulsed Nd:YAG laser (FTSS355-Q, Crystal Laser, Berlin, Germany) working at a 1 or 10 kHz repetition rate at 355 nm (0.5 μJ per pulse) or 532 nm (1.2 μJ per pulse) was used to excite the sample. A 1064 nm rugate notch filter (Edmund Optics) and an uncoated SKG-5 filter (CVI Laser Corporation) were placed in the laser path to remove any NIR emission. The light emitted by the sample was filtered with a 1000 nm long-pass filter (Edmund Optics) and later by a narrow bandpass filter at 1275 nm (BK-1270-70-B, bk Interferenzoptik). A thermoelectric-cooled NIR-sensitive photomultiplier tube assembly (H9170-45, Hamamatsu Photonics, Hamamatsu, Japan) was used as a detector. Photon counting was achieved with a multichannel scaler (NanoHarp 250, PicoQuant). The time dependence of the ^1^O_2_ phosphorescence with the signal intensity *S*(*t*) is described by eq 2, in which τ_T_ and τ_Δ_ are the lifetimes of the photosensitizer triplet state and of ^1^O_2_ respectively, and *S*_0_ is a preexponential parameter proportional to Φ_Δ_.
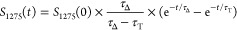
2

The Φ_Δ_ values of the different samples were obtained by comparing *S*_0_ values of optically matched samples and using an appropriate reference by means of eq 3.
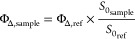
3

The same setup was used to monitor the phosphorescence of the complex and the conjugate, except that the red-sensitive Hamamatsu H5783 photosensor module was used for detection.

### Superoxide Anion Radical Generation and Characterization Using DHR123

All compounds (10 μM) were prepared in PBS (0.2% DMSO). To this solution, DHR123 was added so that its final concentration was 10 μM. Then, the samples were irradiated in 1.0 × 0.5 cm cuvette by green light (505 nm centered LED) for indicated time intervals. Immediately, the fluorescence spectra were collected by using a Photon Technology International (PTI) fluorimeter. The excitation wavelength was set to 500 nm, the excitation and emission slit widths were 2 nm, and the integration time was set to 1 s.

### EPR Studies

EPR measurements were carried out with a Bruker Elexys 580E spectrometer working in X-band at room temperature. Microwave frequency was 9.68 GHz. The modulation amplitude was 0.12 mT. Both the modulation and the microwave power were selected in such a way that no distortion or saturation of the signal was produced. A LED source was used for the irradiation of the sample (530 nm, 450 mW cm^–2^). The spin traps 2,2,6,6-tetramethylpiperidine (TEMP, 60 mM) and 5,5-dimethyl-1-pyrroline-*N*-oxide (DMPO, 360 mM) were dissolved in MeOH. In a typical experiment, 20 μL of TEMP or DMPO solutions was added to an Eppendorf tube containing the compounds (**3a–3c**) in solid form, and the resulting solution was diluted with 280 μL of MeOH (final concentration of conjugates 250 μM). The samples were loaded into capillary tubes, which were placed in the EPR to register the spectrum in dark conditions. Then, the same samples were irradiated for 5 min and the EPR spectrum was registered again.

### Cell Culture and Cell Lines

A375, HeLa, and SK-MEL-28 cells were cultured in Dulbecco’s modified Eagle medium (DMEM) supplemented with 10% FBS, l-glutamine, and 1% penicillin–streptomycin. A2780 and A2780cis cells were grown in RPMI-1640 cell medium supplemented with 10% fetal bovine serum (FBS) and 2 mM l-glutamine. Acquired resistance to cisplatin in the A2780cis cell line was maintained by adding cisplatin 1 μM to culture medium every second passage. Cells were cultured in a humidified incubator at 310 K with 5% CO_2_ atmosphere and subcultured two or three times a week with appropriate densities and were confirmed to be mycoplasma-free using a standard Hoechst DNA staining method.

### Fluorescence Imaging

HeLa Cells were maintained in DMEM containing low glucose (1 g/L) and supplemented with 10% fetal bovine serum (FBS), 50 U/mL penicillin–streptomycin, and 2 mM l-glutamine. For cellular uptake experiments and posterior observation under the microscope, cells were seeded on glass bottom dishes (P35G-1.5-14-C, Mattek). 24 h after cell seeding, cells were incubated at 37 °C for 30 min with Ir(III)-COUPY conjugates **3a–3e** (5 μM) in supplemented DMEM. Then, cells were washed three times with Dulbecco’s phosphate-buffered saline to remove the excess of the compounds and kept in low glucose DMEM without phenol red supplemented with 10 mM Hepes for fluorescence imaging.

All microscopy observations were performed using a Zeiss LSM 880 confocal microscope equipped with a 561 nm laser. The microscope was also equipped with a heating insert (P Lab-Tek S, Pecon) to keep cells at 37 °C. Cells were observed using a 63× 1.4 oil immersion objective. The compounds were excited using the 561 nm laser and emission detected from 570 to 670 nm. Image analysis was performed using Fiji.^65^ All images are colorized using the Fire lookup table from Fiji.

### Cellular Accumulation by ICP–MS

Briefly, A2780cis cells were seeded onto a 12-well plate (3 × 10^5^ cells/well). Treatments with tested agents for 2 h were applied at 10 μM. Cisplatin was included for comparative purposes. Cells were then trypsinized, and pellets were counted. Samples were then digested with 30% HNO_3_ suprapur acid (Sigma-Aldrich) and subjected to Inductively Coupled Plasma Mass Spectrometry analysis in Agilent 7900 ICP–MS. ^99^Ru, ^101^Ru, ^194^Pt, and ^195^Pt isotopes were measured. Three independent experiments were performed (*n* = 2 replicates).

### Photo- and Cytotoxic Activity Determination

A2780, A2780cis, HeLa, A375, and SK-MEL-28 cells were maintained at logarithmic growth-phase and cultured in 96-well plates at a density of 5000 cells/well in complete medium for 24 h at 310 K, 5% CO_2_ in a humidified incubator. For hypoxia experiments, a hypoxia condition was set up by Tissue Culture Service at University of Murcia using nitrogen (N_2_) to displace O_2_ down to a minimum of 2% in a Forma Steri-Cycle i160 incubator (Thermo Fisher Scientific). Serial dilutions of the compounds were added at the final concentrations in the range of 0–100 μM in a final volume of 100 μL per well. A light-based treatment schedule was performed as follows: 1 h incubation with the compounds in the dark, followed by 1 h incubation under irradiation conditions by placing the Photoreactor EXPO-LED from Luzchem (Canada) fitted with green lamps (final light intensity applied of 1.5 mW/cm^2^ at λ_max_ = 520 nm) inside the CO_2_ incubator. All the cell culture plates subjected to light irradiation included untreated controls to verify that cell viability was not affected by light. Then, treatment-containing media was removed, and fresh media was added for a 48 h cell recovery period; the temperature throughout the experiment remained at 310 K. Dark control samples were placed in the same dark conditions and then kept incubated for 1 h in the dark in the humidified CO_2_ incubator. Medium was then aspirated by suction, cells were loaded with 50 μL of MTT solution (1 mg/mL) for additional 4 h and then removed, and 50 μL of DMSO was added to solubilize the purple formazan crystals formed in active cells. The absorbance was measured at 570 nm using a microplate reader (FLUOstar Omega), and the IC_50_ values were calculated based on the inhibitory rate curves using the following equation
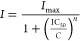
4where *I* represents the percentage inhibition of viability observed, *I*_max_ is the maximal inhibitory effect, IC_50_ is the concentration that inhibits 50% of maximal growth, *C* is the concentration of the treatment, and *n* is the slope of the semi-logarithmic dose–response sigmoidal curves. The non-linear fitting was performed using SigmaPlot 14.0 software. All experiments were performed in three independent studies with *n* = 3 replicates per concentration level. For a detailed phototoxicity procedure, ref (43) includes technical explanations.

### ROS Photogeneration in A2780cis Cells

ROS levels were determined using the 2′-7′dichlorofluorescein diacetate (DCFH-DA) probe. A2780cis cells were seeded onto 96-well black plates at 2 × 10^4^cells/well for 24 h in a humidified CO_2_ incubator either in normoxia (21% O_2_) or hypoxia (2% O_2_). Tested compounds were then administered in cell media for the allowed time. Treatments were then removed, and cells were stained with 10 μM of DCFH-DA for 0.5 h. After staining, cells were washed with PBS twice and irradiated for 1 h with LED source light from Luzchem photoreactor (Canada) fitted with green lamps (final light intensity applied of 1.5 mW/cm^2^ at λ_max_ = 520 nm). Fluorescence readings were performed in FLUOstar Omega (λ_exc_ = 488 nm and λ_em_ = 530 ± 30 nm). Non-irradiated plates were used for dark conditions, whereas treated, unstained cells were used to subtract basal fluorescence of compounds and correct fluorescence readings. Unstained cells served as blank. Three independent experiments were performed with *n* = 3 replicates.

### Cell Death Induction Assays

Cell death induction was evaluated using the Annexin V-FITC/Propidium iodide (AV/PI) dual staining method. Briefly, A2780cis cells were seeded in 12-well plates at a density of 2 × 10^5^ cells/well and incubated overnight at 310 K. Tested compounds (10 μM) were added, following the described treatment schedule (1 h incubation + 1 h irradiation with 520 nm light). Irradiated, non-treated cells served as the control group. After 24 h of the drug-free recovery period, cells were harvested by trypsinization, washed with PBS and centrifuged, and the pellets were resuspended in 200 μL of a binding buffer as instructed by the manufacturer (Cayman). The resuspended cell solution was left at room temperature in the dark for 15 min before analysis by flow cytometry (FACSCalibur BecktonDickinson; 10^4^ events acquired per sample). Cells were visualized using λ_exc_ = 488 channels and registered at 525 nm and 620 nm for Annexin V and propidium iodide in FL1 and FL2 channels, respectively. Cell populations were classified as follows: AV^-^/PI^-^ (viable cells); AV^-^/PI^+^ (necrotic cells); AV^+^/PI^-^ and AV^+^/PI^+^ (apoptotic cells). Alternatively, A2780cis cells were subjected to flow cytometry after phototreatments and morphological changes were analyzed using FSC and SSC contour plots. Data were analyzed using FlowJo software. Three independent experiments were performed.

### 3D Multicellular Tumor Spheroid Growth Inhibition Assays

Briefly, a single suspension of A2780cis cells at a density of 5 × 10^3^ cells/well in complete RPMI medium was dispatched onto 96-well Corning microplates with ultralow attachment surface coating. The plates were covered and transferred to an incubator at 310 K with a 5% CO_2_ atmosphere. After 3 days post-seeding, uniform MCTSs were formed, which was confirmed using an inverted Zeiss AXIO observer 7 microscope. Cell media of MCTSs was changed by replacing 50% with fresh cell media and allowed to grow for 3 days. On day 3, MTCS was incubated with tested agents (10 μM) for 4 h and then irradiated with 520 nm light for 1 h. Treatments were then carefully removed, and fresh media was added. The integrity, radius, size, and volume of the MCTSs were monitored using a DMi1 inverted phase contrast microscope (Leica Microsystems) over 7 days. The radius of the tumorspheres was measured using Fiji software, and the volume was calculated using the following equation: *V* = 4/3π*r*^3^.

## Supporting Information Available

Photophysical and photochemical characterization of the compounds and copies of NMR and MS spectra (PDF)
Molecular formula strings (CSV)

## Abbreviations

ACN: acetonitrile
A2780: cisplatin-sensitive ovarian carcinoma
A2780cis: cisplatin-resistant ovarian carcinoma
A-375: female human malignant melanoma cell line
DCM: dichloromethane
DCFH-DA: 2′-7′dichlorofluorescein diacetate
DHE: dihydroethidium
DHR123: dihydrorhodamine 123
DIPEA: *N*,*N*-diisopropylethylamine
DMEM: Dulbecco modified Eagle medium
DMSO: dimethylsulfoxide
EDG: electron-donating group
EPR: electron paramagnetic resonance
ESI: electrospray ionization
FBS: fetal bovine serum
HATU: 1-[bis(dimethylamino)methylene]-1*H*-1,2,3-triazolo[4,5-*b*]pyridinium 3-oxid hexafluorophosphate
HeLa: cervical carcinoma cell line
HRMS: high-resolution mass spectrometry
ICD: immunogenic cell death
ICP-MS: inductively coupled-plasma mass spectrometry
ISC: intersystem crossing
LW: Lawesson’s reagent
NIR: near-infrared
NMR: nuclear magnetic resonance
PBS: phosphate-buffered saline
PDT: photodynamic therapy
PI: phototherapeutic index
PpIX: protoporphyrin IX
PS: photosensitizer
ROS: reactive oxygen species
SK-MEL-28: male human malignant melanoma cell line
TLC: thin layer chromatography
