# Supplementary material for: Exploring Structure–Activity Relationships in Photodynamic Therapy Anticancer Agents Based on Ir(III)-COUPY Conjugates
Source: J Med Chem. 2023 Jun 2;66(12):7849–67. doi: 10.1021/acs.jmedchem.3c00189 (PMC10291553; doi:10.1021/acs.jmedchem.3c00189)
Supplement: Supplementary file 1 — jm3c00189_si_001.pdf [file jm3c00189_si_001.pdf]

SUPPORTING INFORMATION

Exploring structure–activity relationships in photodynamic therapy anticancer agents based on Ir(III)-COUPY conjugates

Anna Rovira,[1,#] Enrique Ortega-Forte,[2,#] Cormac Hally,[3] Mireia Jordà-Redondo,[3] Diego Abad-Montero,[3] Gloria Vigueras,[2] Jesús I. Martínez,[4] Manel Bosch,[5] Santi Nonell,[3,*] José Ruiz,[2,*] Vicente Marchán[1,*]

[1] Departament de Química Inorgànica i Orgànica, Secció de Química Orgànica, Universitat de Barcelona (UB), and Institut de Biomedicina de la Universitat de Barcelona (IBUB), Martí i Franquès 1-11, E-08028 Barcelona, Spain. Email: vmarchan@ub.edu

[2] Departamento de Química Inorgánica, Universidad de Murcia, and Institute for Bio-Health Research of Murcia (IMIB-Arrixaca), E-30071 Murcia, Spain. Email: jruiz@um.es

[3] Institut Químic de Sarrià, Universitat Ramon Llull, Vía Augusta 390, E-08017 Barcelona (Spain). Email: santi.nonell@iqs.url.edu

[4] Instituto de Nanociencia y Materiales de Aragón (INMA), CSIC-Universidad de Zaragoza, E-50009 Zaragoza (Spain)

[5] Unitat de Microscòpia Òptica Avançada, Centres Científics i Tecnològics, Universitat de Barcelona, Av. Diagonal 643, E- 08028 Barcelona (Spain)

# These authors contributed equally

Table of contents

1.- Photophysical and photochemical characterization of the compounds S3

2.- *In vitro* photobiological studies S8

3.- ^{1}H and ^{13}C NMR spectra and HR ESI-MS of the compounds S12

4.- HPLC analysis of the compounds S45

1. Photophysical and photochemical characterization of the compounds

Table S1. Photophysical and photochemical properties of the control compounds (coumarins **1a-1c** and complex **2a**) in different solvents at room temperature.

Comp.	Solvent	λ_{abs} [nm]	ε [mM·cm^{-1}]	λ_{em} [nm]	Stokes' shift [nm]	ϕ_f or ϕ_p	τ_F [ns]	τ_P [ns]	Φ_Δ at 355 nm	Φ_Δ at 532 nm
**1a**	DCM	569	67	607	38	0.70	5.4	-	<0.01	0.03
	ACN	548	75	609	61	0.18	1.4	-	<0.01	<0.01
	PBS	545	34	604	59	0.14	0.9	-	≈ 0	<0.01
**1b**	DCM	456	23	506	50	0.007	3.52	-	0.01	-
	ACN	446	24	492	46	0.002	4	-	0.01	<0.01
	PBS	443	23	486	43	0.002	3.37	-	≈ 0	<0.01
**1c**	DCM	597	61	631	34	0.53	5.46	-	0.03	0.03
	ACN	572	60	635	63	0.22	2.03	-	≈ 0	0.01
	PBS	557	38	629	72	0.031	0.031	-	≈ 0	<0.01
**2a**	DCM	303	42	665	362	0.07	-	315	0.23	-
	ACN	302	41	660	358	0.03	-	187	0.42	-
	PBS	305	40	656	351	>0.01	-	55 (93%)<br>281 (7%)	<0.01	-

Table S2. Photophysical and photochemical properties of the Ir(III)-COUPY conjugates **3a-3e** in different solvents at room temperature.

Comp.	Solvent	λ_{abs} [nm]	ε [mM·cm^{-1}]	λ_{em} [nm]	Stokes' shift [nm]	ϕ_f or ϕ_p	τ_F [ns]	τ_P [ns]	Φ_Δ at 355 nm	Φ_Δ at 532 nm
**3a**	DCM	566	44	602	36	0.07	0.25	121 (70%)<br>392 (30)	0.37	0.34
	ACN	555	22	615	60	0.08	0.51	45 (86%)<br>269 (14%)	0.23	0.24
	PBS	550	17	615	65	0.004	0.37 (73%)<br>3.3 (27%)	-	<0.01	<0.01
**3b**	DCM	457	26	-	-	0.01	-	329	0.21	-
	ACN	451	33	-	-	0.002	-	173	0.30	0.32
	PBS	436	17	-	-	0.00049		39 (52%)<br>211 (48%)	<0.01	<0.01
**3c**	DCM	592	26	629	37	0.17	2.66	69 (80%)<br>298 (20%)	0.20	0.16
	ACN	580	24	647	67	0.08	0.9 (74%)<br>3.534 (26%)	264	0.14	0.12
	PBS	575	13	610	35	0.003	1.4	317 (78%)<br>43 (22%)	<0.01	<0.01
**3d**	DCM	567	45	599	32	0.09	2.94	333 (89%)<br>59 (11%)	0.39	0.35
	ACN	556	45	608	52	0.07	2.94 (70%)<br>0.79 (30%)	263	0.32	0.30
	PBS	550	21	612	62	0.017	1.11	307 (72%)<br>1900 (28%)	<0.01	<0.01
**3e**	DCM	566	40	600	34	0.08	0.55 (62%)<br>3.34 (38%)	377 (64%)<br>109 (36%)	0.34	0.28
	ACN	554	41	607	53	0.16	1	159 (56%)<br>40 (44%)	0.31	0.22
	PBS	541	20	618	77	0.006	1.03	315 (68%)<br>1913 (32%)	<0.01	<0.01

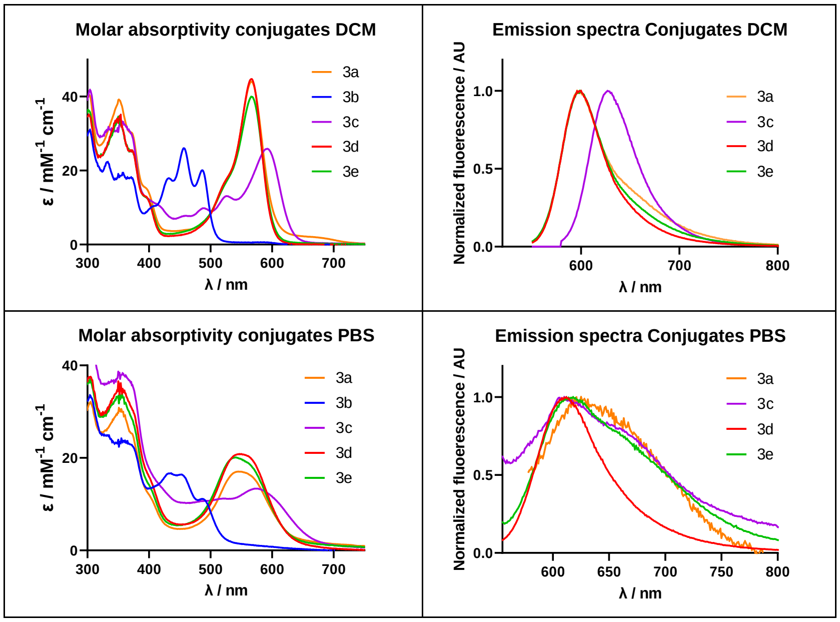

Figure S1. Comparison of the absorption and emission spectra of Ir(III)-COUPY conjugates **3a-3e** in DCM and PBS.

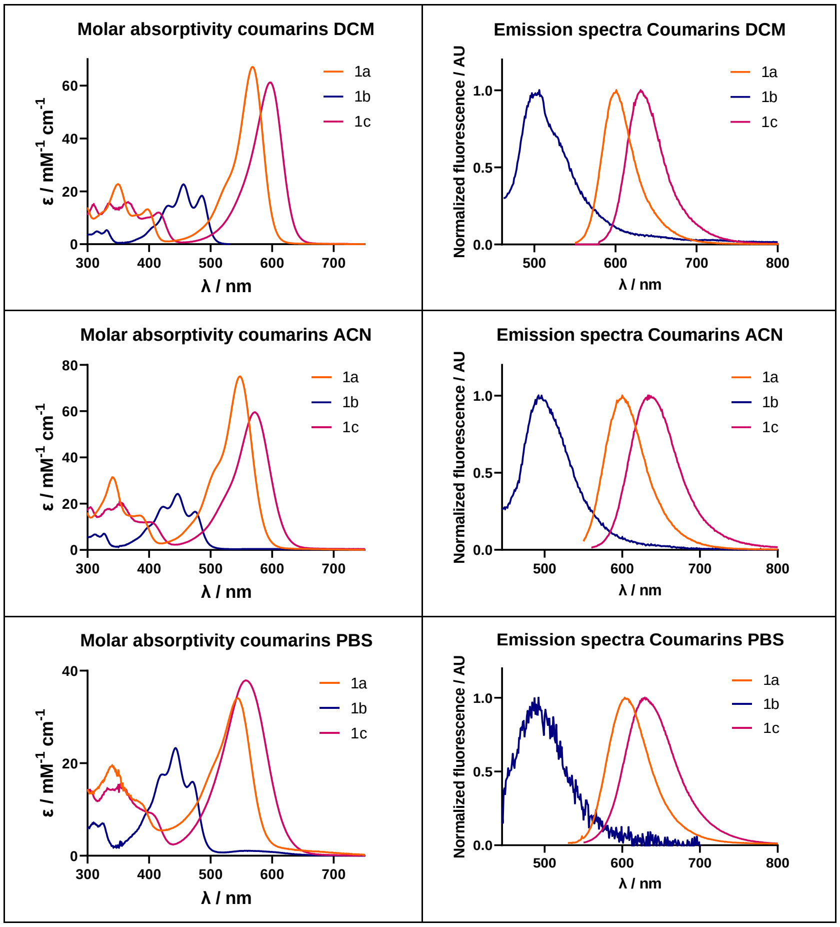

Figure S2. Comparison of the absorption and emission spectra of COUPY dyes **1a-1c** in DCM, ACN and PBS.

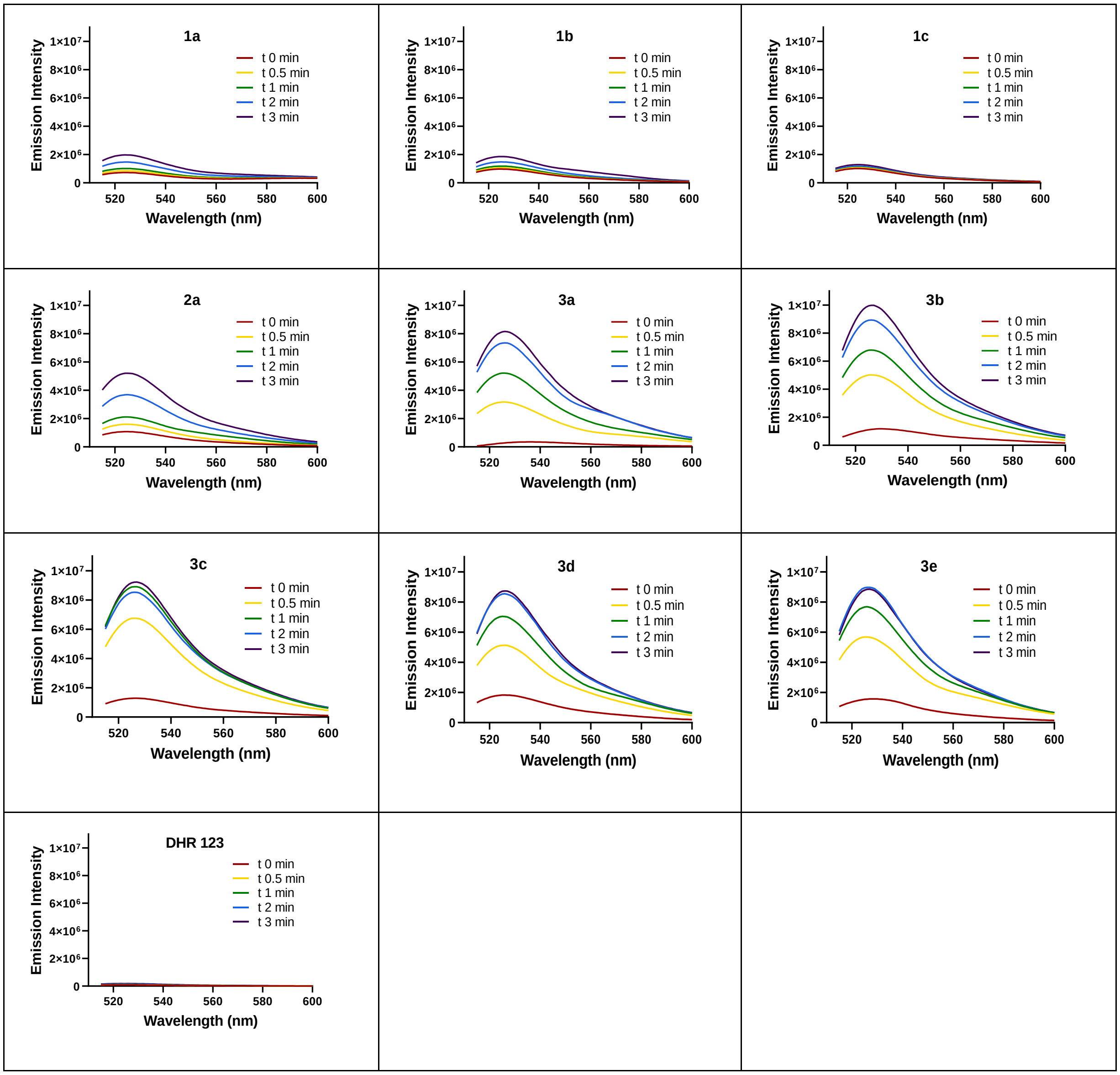

Figure S3. Increase of the fluorescence spectra emission of DHR123 upon photoirradiation of COUPY coumarins **1a-1c**, Ir(III) complex **2a**, Ir(III)-COUPY conjugates **3a-3e** or without any compound (DHR 123 alone) at 505 nm in PBS (0.2 % DMSO). DHR123 fluorescence was excited at 500 nm.

2. *In vitro* photobiological studies

Table S3. Phototoxicity of the compounds towards A2780cis cells under normoxia and hypoxia.[a]

	Normoxia			Hypoxia		
	**Dark**	**520 nm**	**PI[b]**	**Dark**	**520 nm**	**PI[b]**
**1a**	>250	2.1 ± 0.2	>119.1	>250	2.5 ± 0.2	>100.0
**1b**	>250	>250	n.d.	>250	>250	n.d.
**1c**	15 ± 2	0.15 ± 0.04	100.0	20 ± 2	0.6 ± 0.1	36.4
**2a**	>250	3.5 ± 0.4	>71.4	>250	11 ± 2	>22.7
**3a**	>250	0.70 ± 0.06	>357.1	>250	3.8 ± 0.3	>65.8
**3b**	>250	61 ± 8	>4.1	>250	31 ± 7	>8.1
**3c**	>250	1.04 ± 0.02	>240.4	>250	8 ± 1	>31.3
**3d**	>250	1.1 ± 0.2	>227.3	>250	1.9 ± 0.2	>131.6
**3e**	>250	0.93 ± 0.04	>268.8	>250	1.7 ± 0.3	>147.1
**5-ALA**	>250	62 ± 12	>4.0	>250	>250	n.d.

[a]Cells were treated for 2 h (1 h incubation and 1 h irradiation with green light) followed by 48 h of incubation in drug-free medium either under normoxic (21 % O_2) or hypoxic (2 % O_2) conditions. Dark analogues were kept in the dark. Data expressed as mean ± SD from three independent experiments.
[b]PI = phototherapeutic index defined as IC_{50} (dark-non-irradiated cells)/IC_{50} (irradiated cells).

Table S4. Phototoxicity of the **3a** and **3b** towards A2780cis cells using blue light (465 nm).[a]

	**Dark**	**465 nm**	**PI**[b]
**3a**	>100	1.9 ± 0.3	>52.6
**3b**	>100	2.4 ± 0.2	>41.7

[a]Cells were treated for 2 h (1 h incubation and 1 h irradiation with blue light, 5 mW/cm^2) followed by 48 h of incubation in drug-free medium. Dark analogues were kept in the dark. Data expressed as mean ± SD from three independent experiments. [b]PI = phototherapeutic index defined as IC_{50} (dark-non-irradiated cells)/IC_{50} (irradiated cells).

Table S5. Partial inhibition (% of cell growth inhibition with respect to untreated controls) of compounds at 250 µM in A2780cis after 2 h incubation in the dark followed by 48 h drug-free recovery period.

	Normoxia	Hypoxia
**1a**	3.5 ± 0.9	2.1 ± 0.4
**1b**	24 ± 3	4.5 ± 0.6
**1c**	100	100
**2a**	62 ± 4	56 ± 6
**3a**	58 ± 7	49 ± 4
**3b**	63 ± 5	45 ± 6
**3c**	74 ± 10	61 ± 8
**3d**	21 ± 1	27 ± 4
**3e**	18 ± 1	12 ± 3
**5-ALA**	3.5 ± 0.4	1.0 ± 0.2

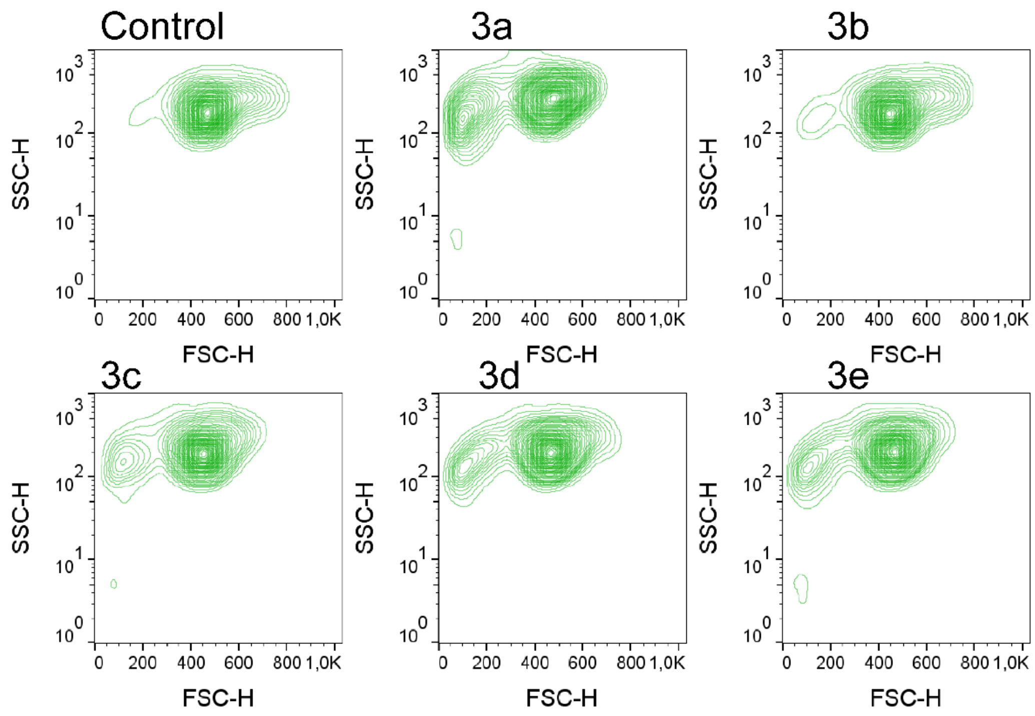

Figure S4. Cell size (FSC) and cell complexity (SSC) contour plots of A2780cis cells after treatment with Ir(III)-COUPY conjugates (10 µM, 1 h) upon 520 nm light irradiation for 1 h.

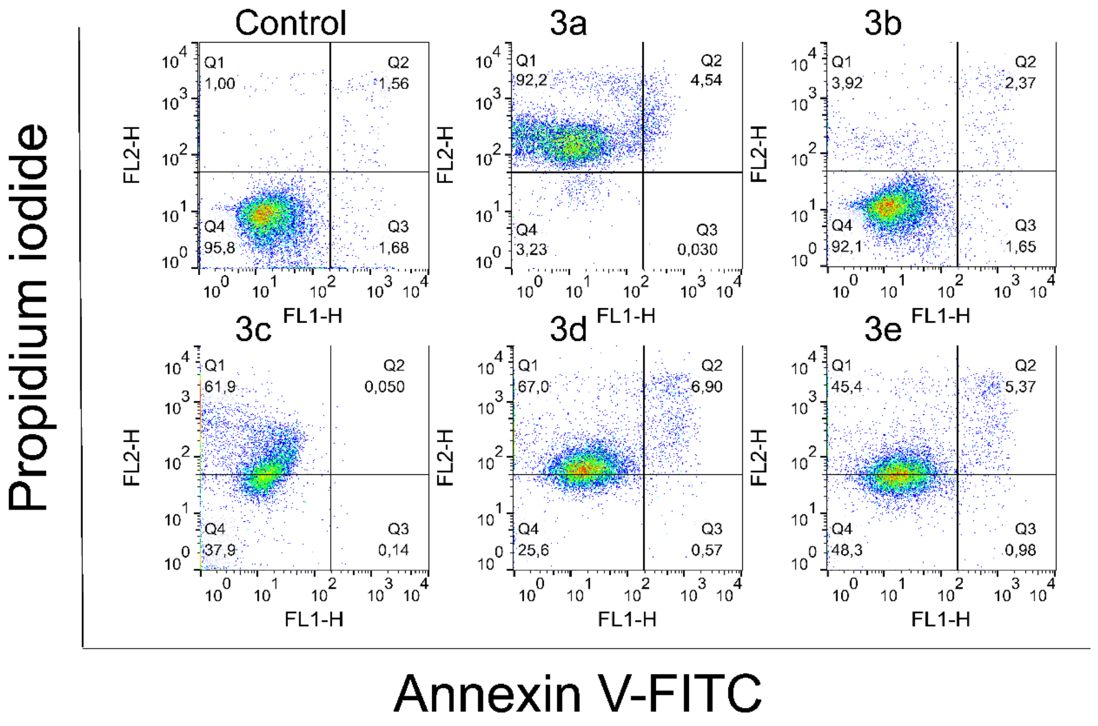

Figure S5. Representative double-stained Annexin V-FITC (FL1)/Propidium iodide (FL2) dot plots of A2780cis cells after treatment with Ir(III)-COUPY conjugates (10 µM, 1 h) upon 520 nm light irradiation for 1 h.

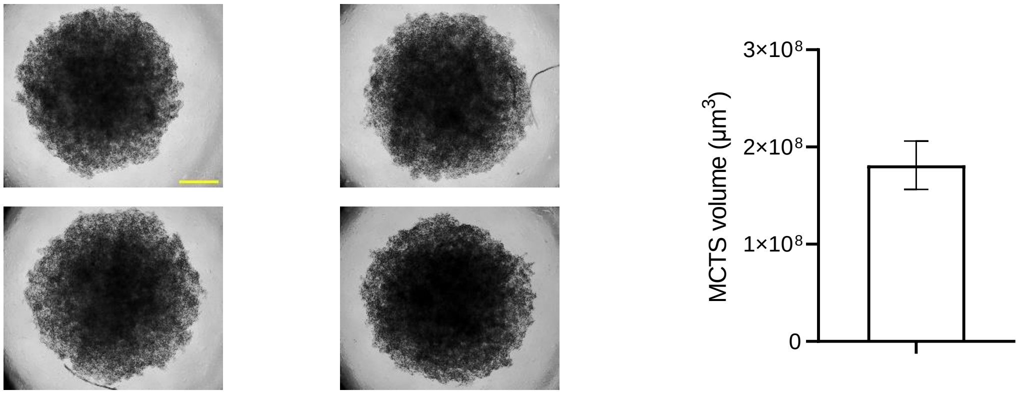

Figure S6. Representative A2780cis MCTSs generated after 3 days post-seeding with uniform average volume (n=24). Scale bar: 200 μm.

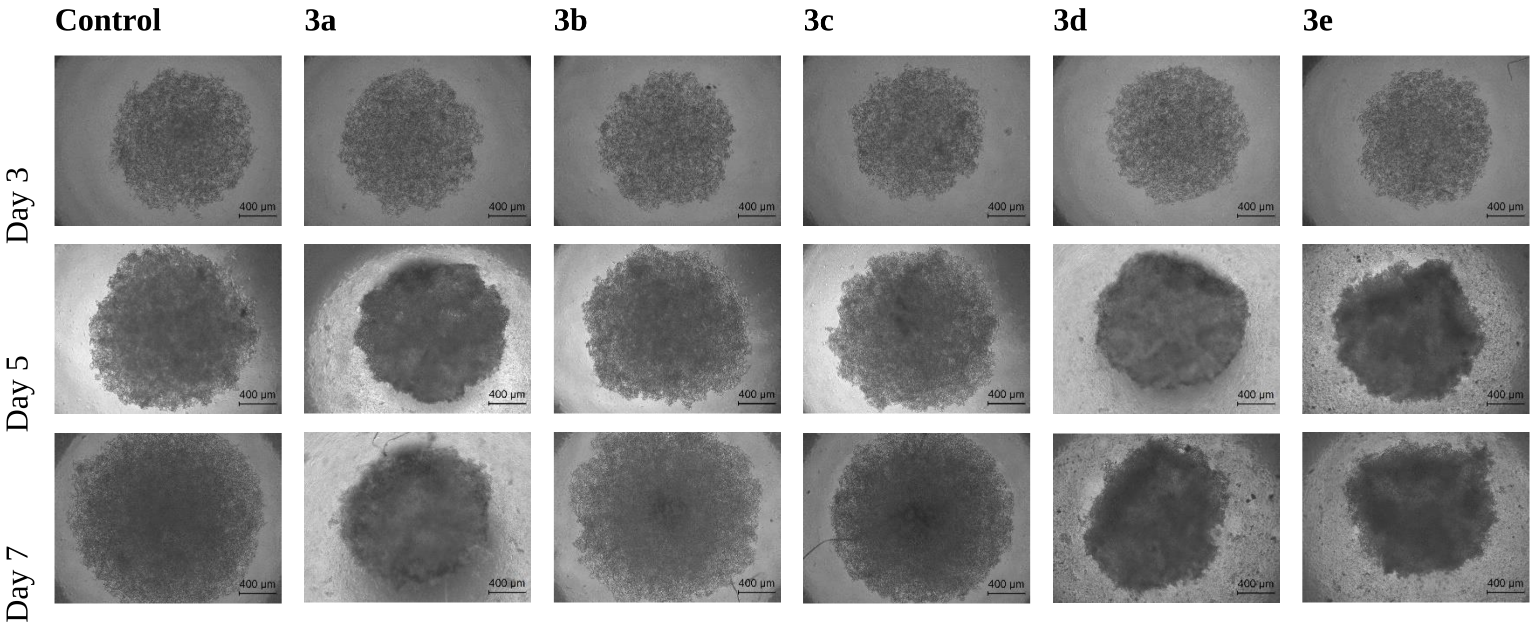

Figure S7. Changes in relative volume in A2780cis MCTS throughout 7 days. PDT treatments were applied on day 3. Scale bar: 400 μm.

3. ^{1}H and ^{13}C NMR spectra and HR ESI-MS of the compounds

Compound 4

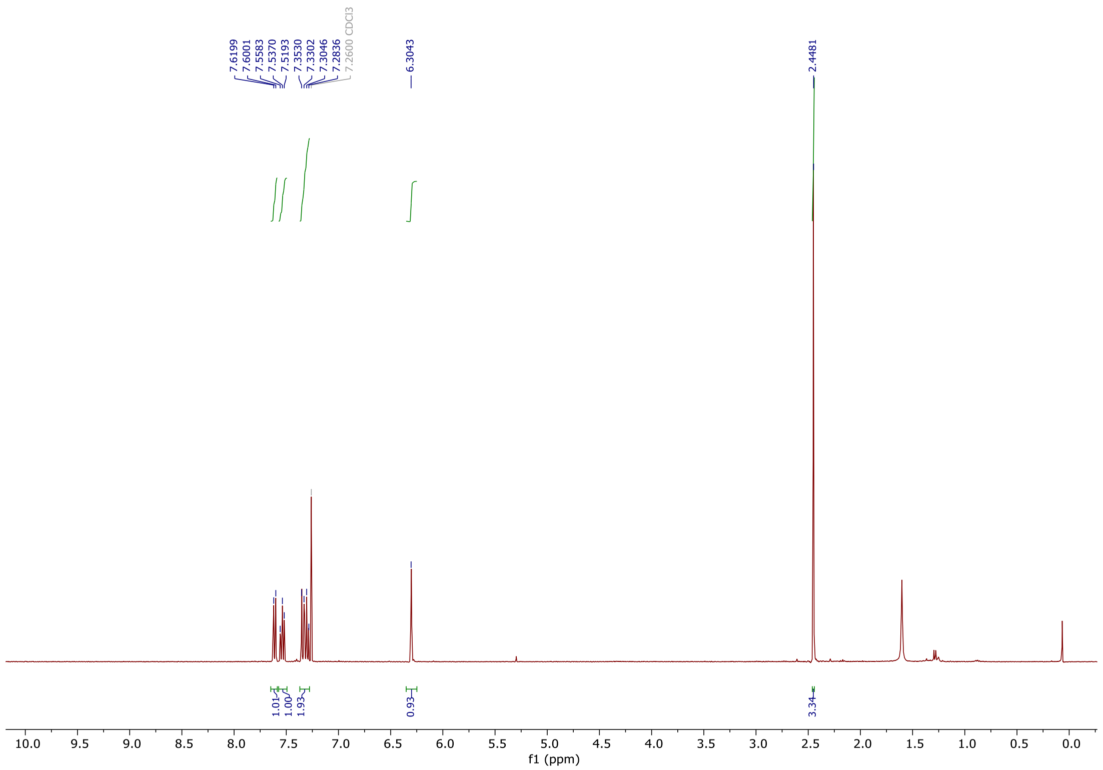

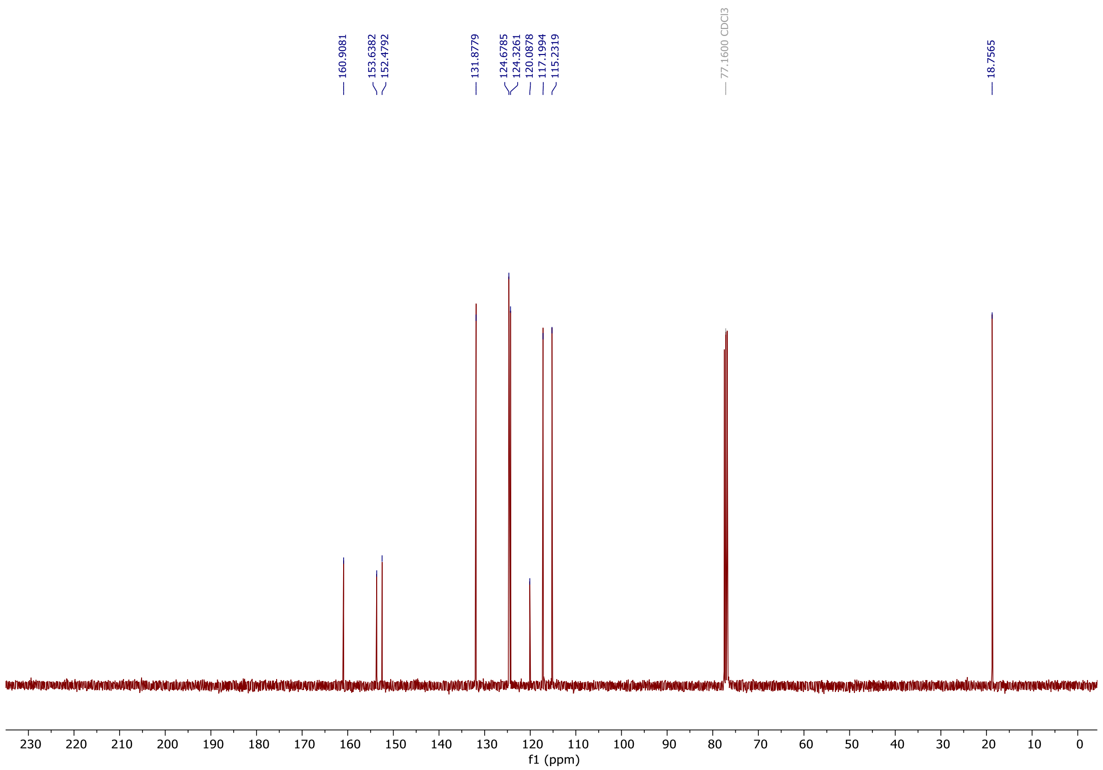

Figure S8. ^{1}H and ^{13}C NMR spectra of compound **4** in $CDCl_3$.

Compound 5

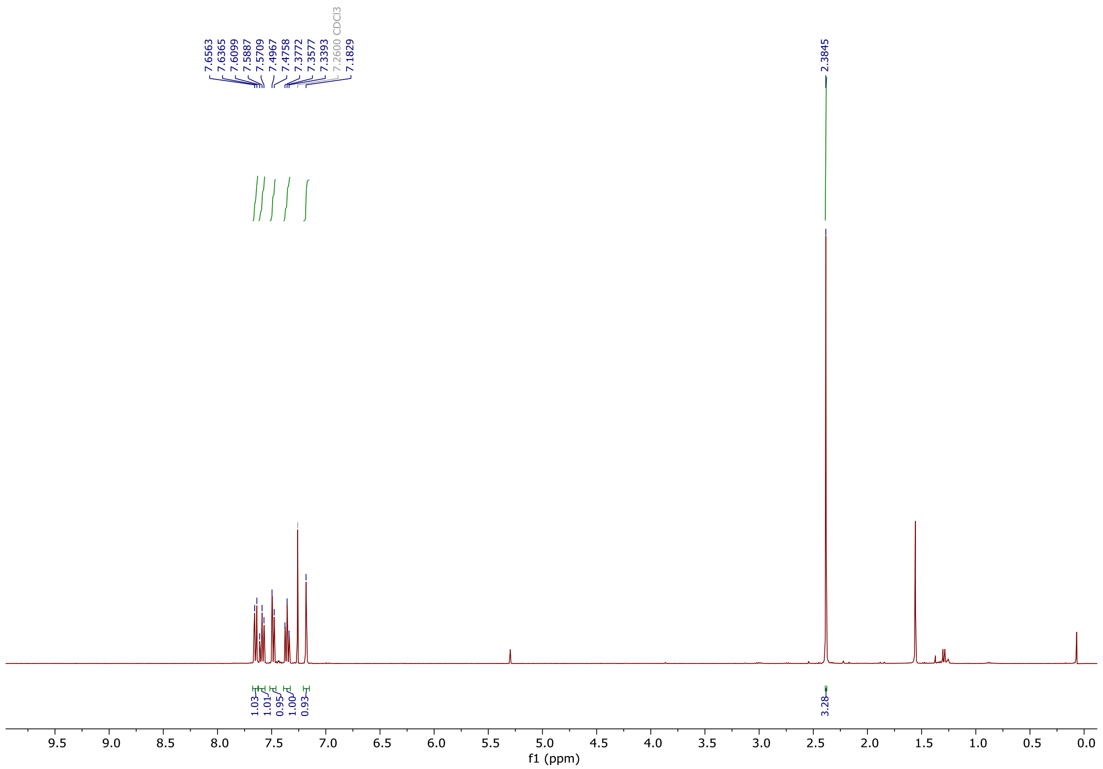

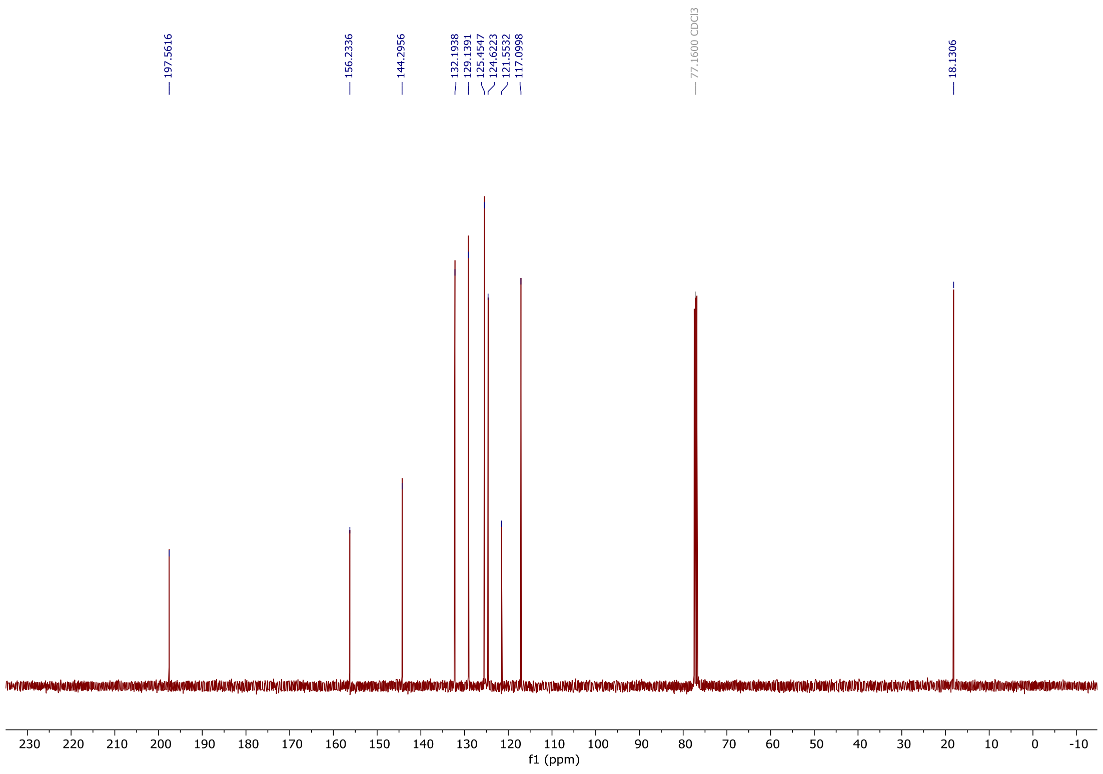

Figure S9. ^{1}H and ^{13}C NMR spectra of compound **5** in $CDCl_3$.

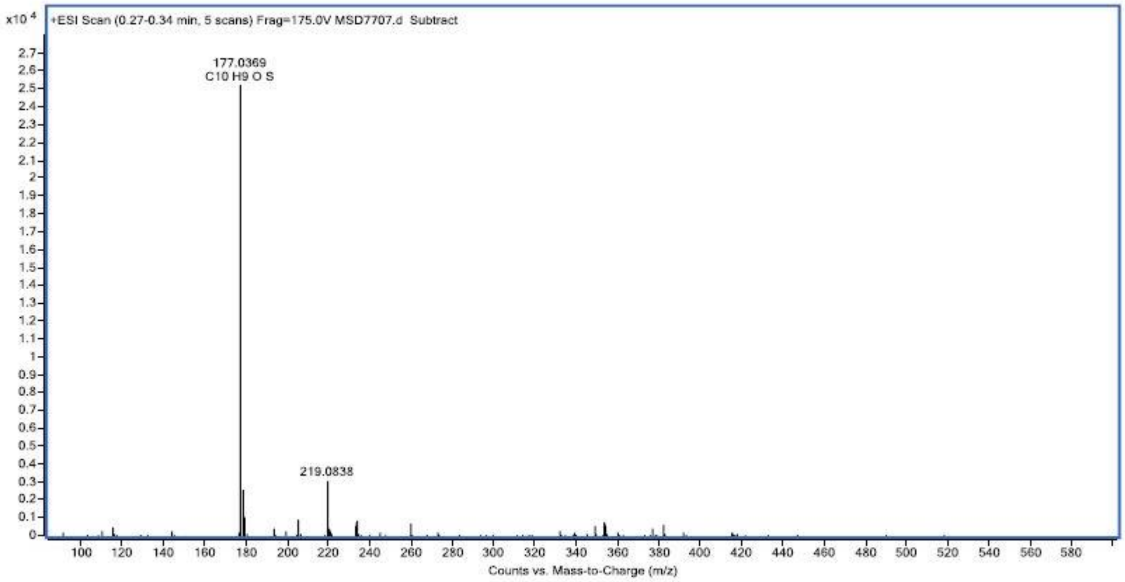

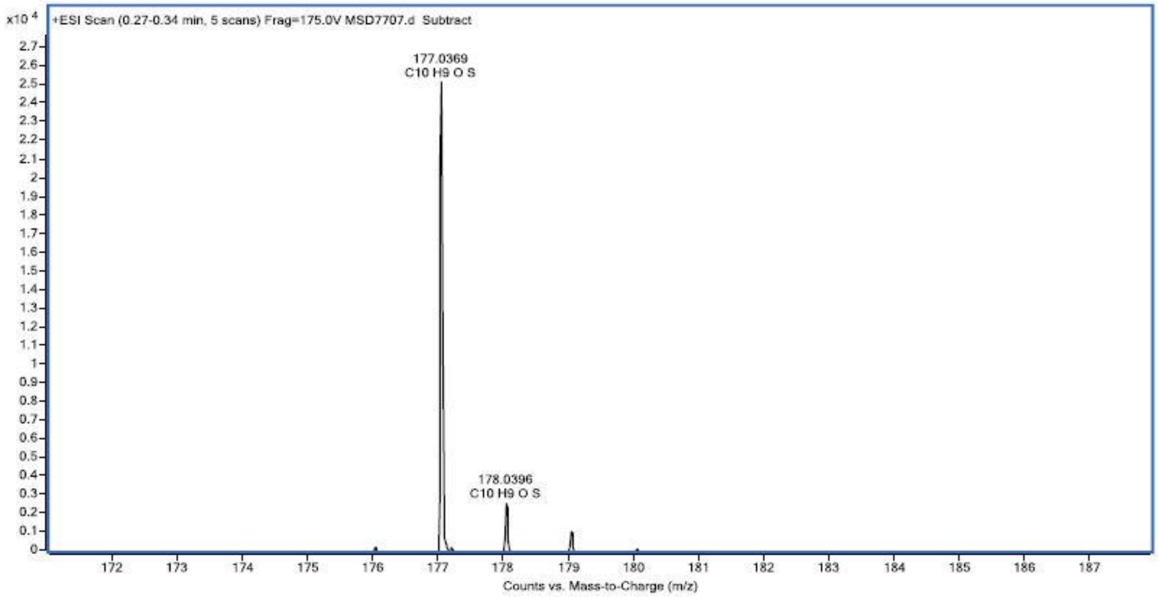

Figure S10. HR ESI-MS spectrum of compound **5**.

Compound 6

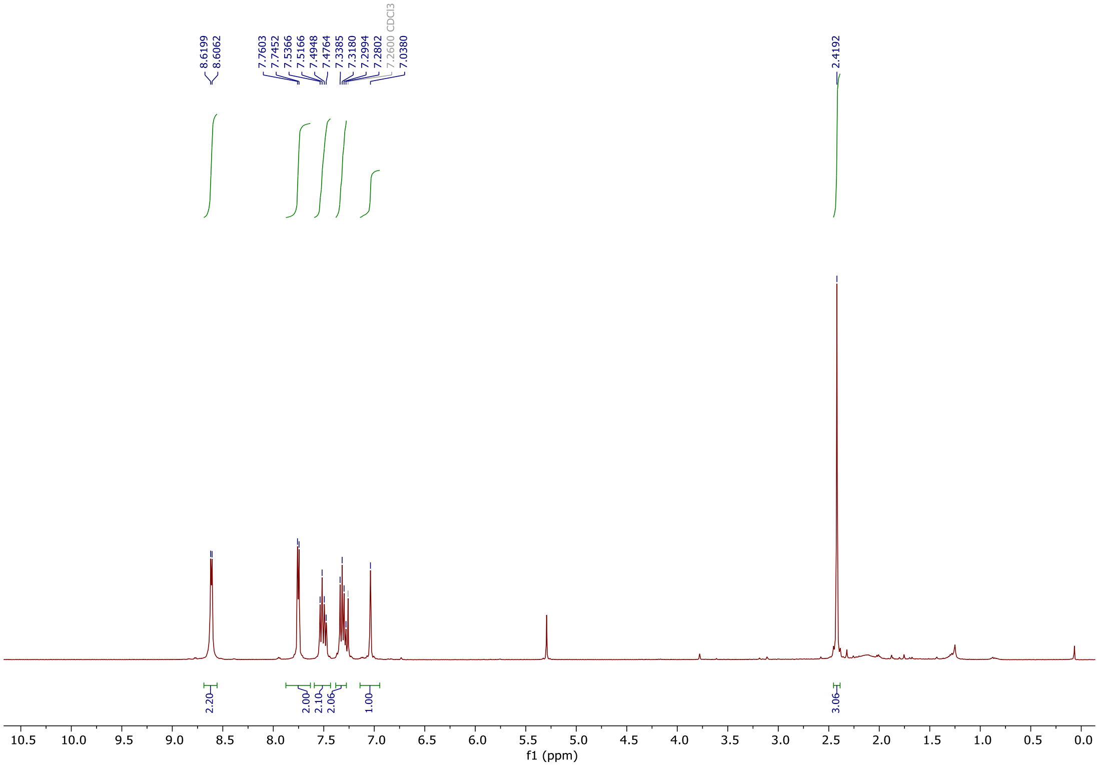

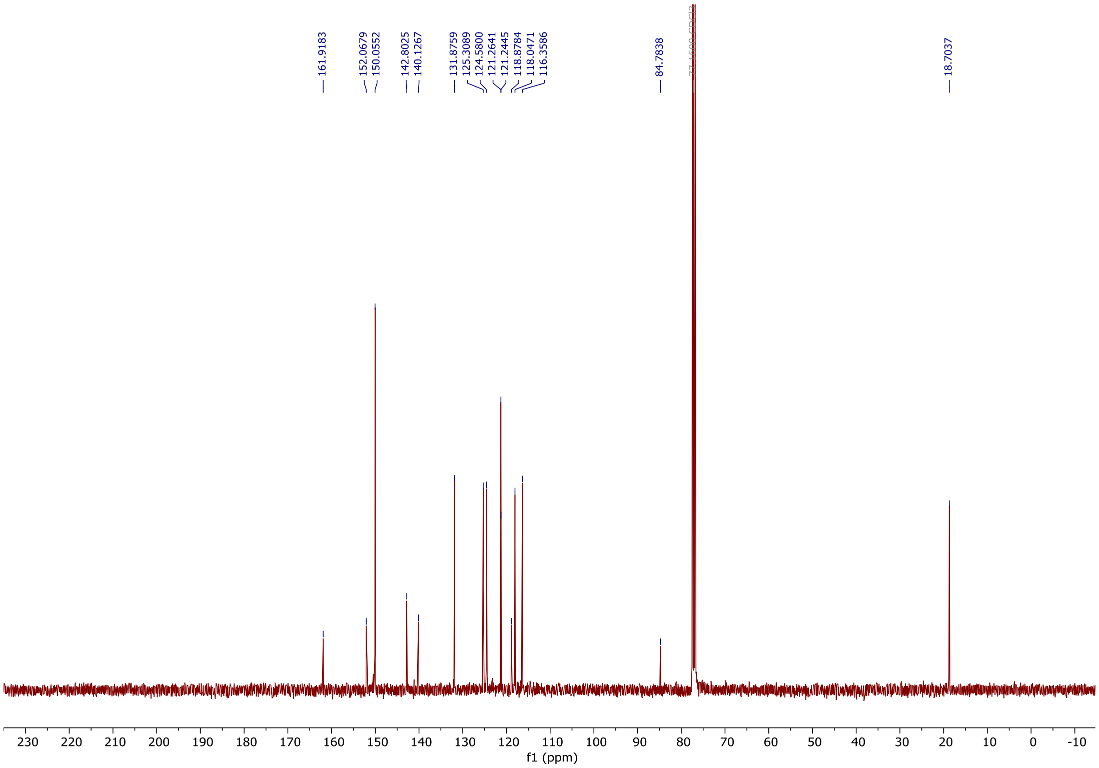

Figure S11. ^{1}H and ^{13}C NMR spectra of compound **6** in $CDCl_3$.

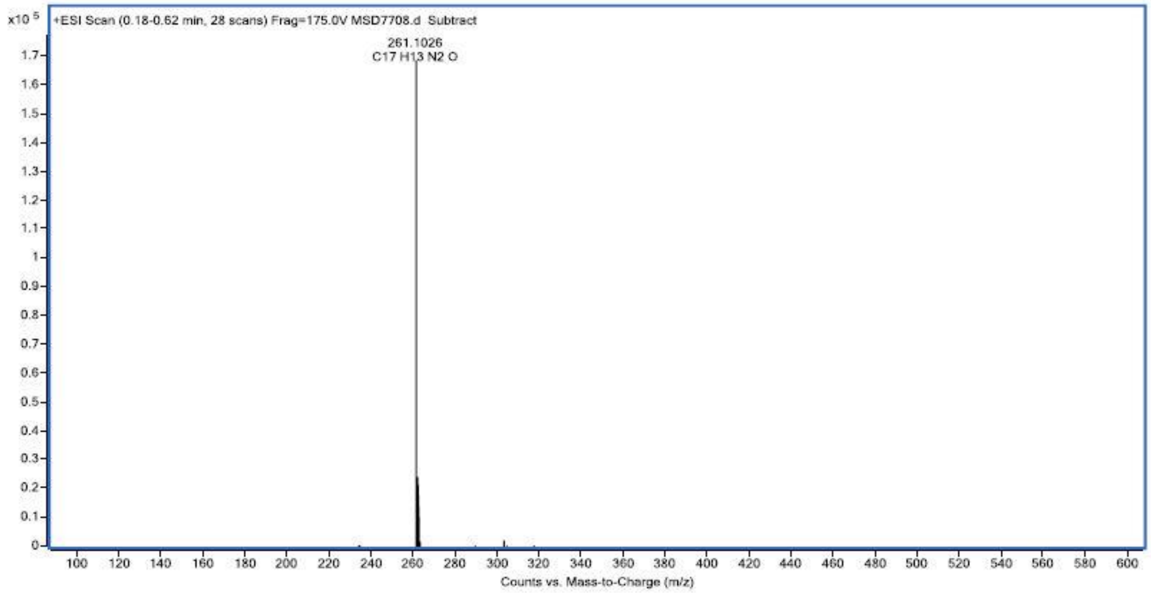

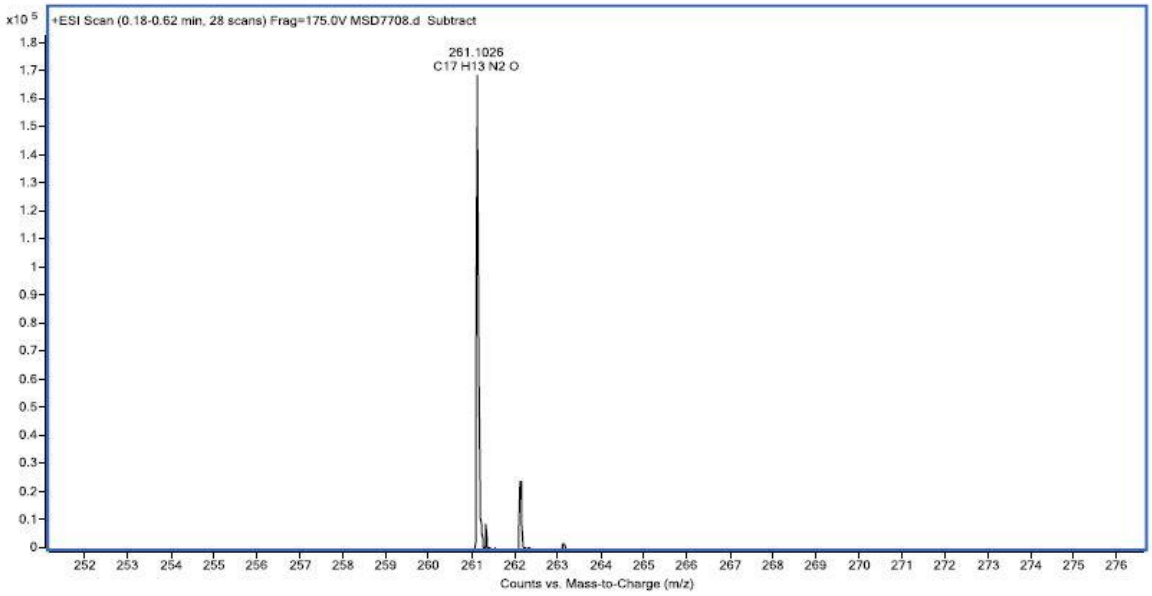

Figure S12. HR ESI-MS spectrum of compound **6**.

Compound 7

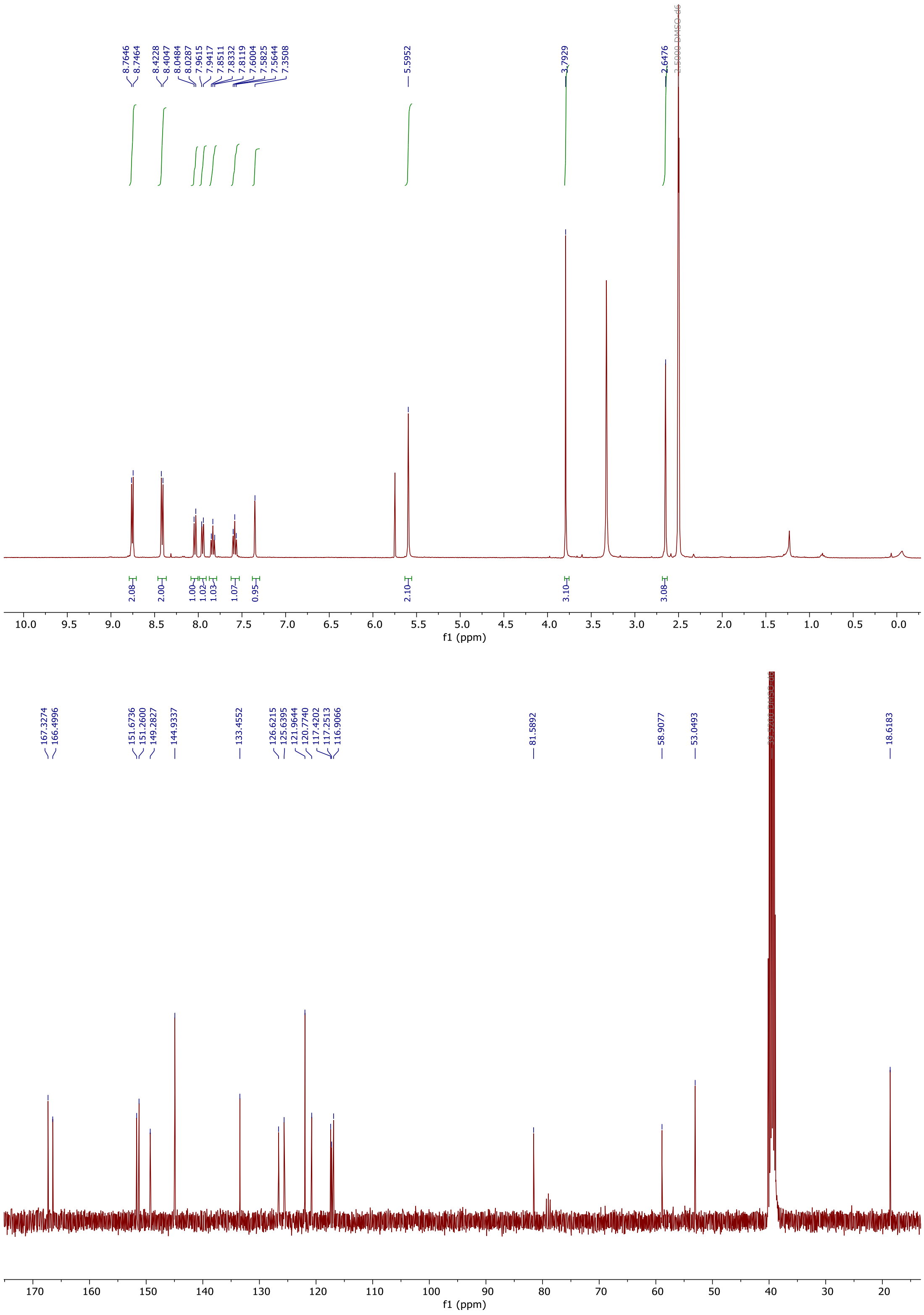

Figure S13. ^{1}H and ^{13}C NMR spectra of compound **7** in DMSO-*d*$_6$.

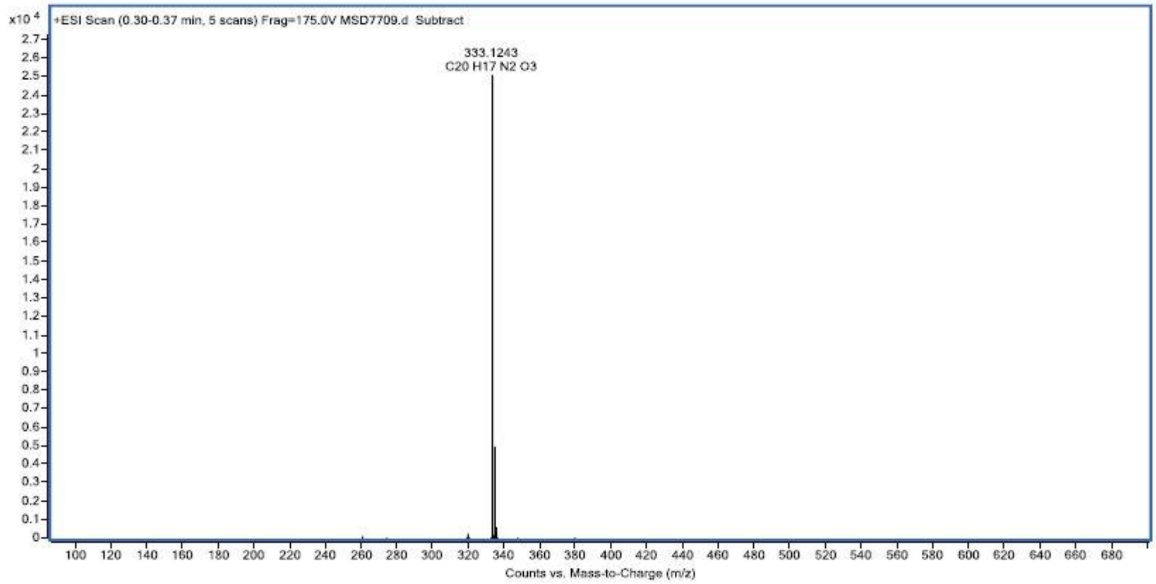

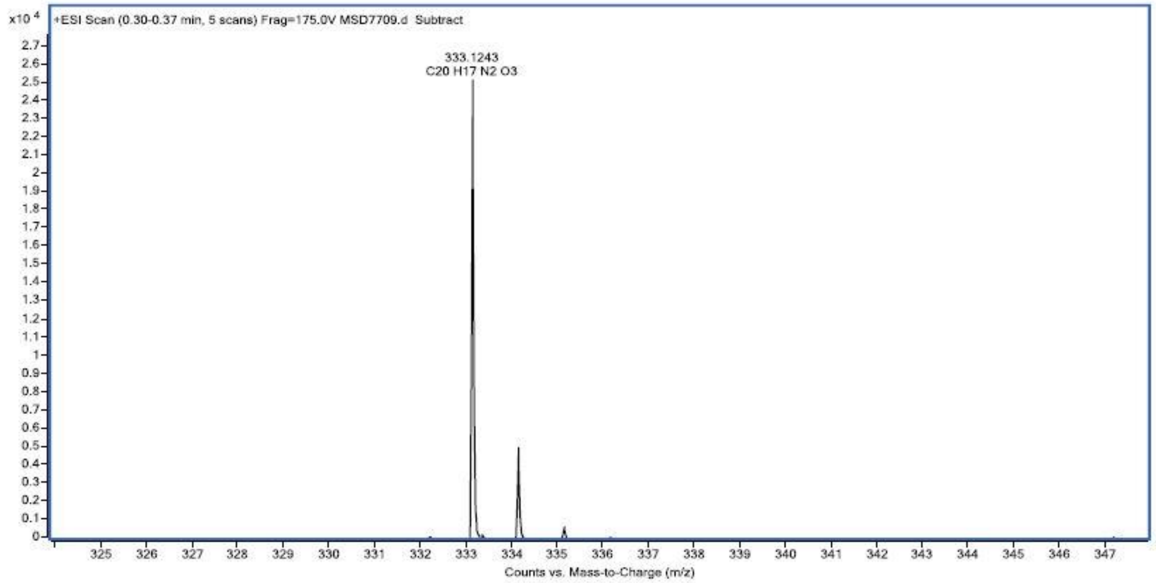

Figure S14. HR ESI-MS spectrum of compound **7**.

Compound 9

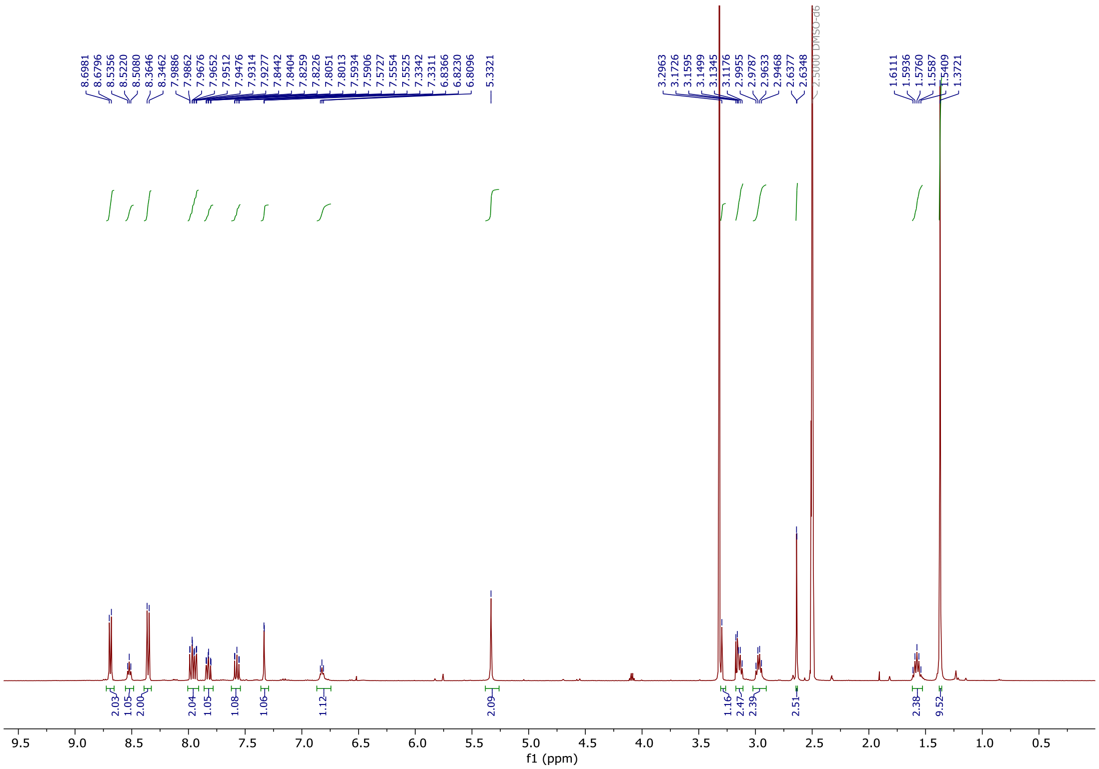

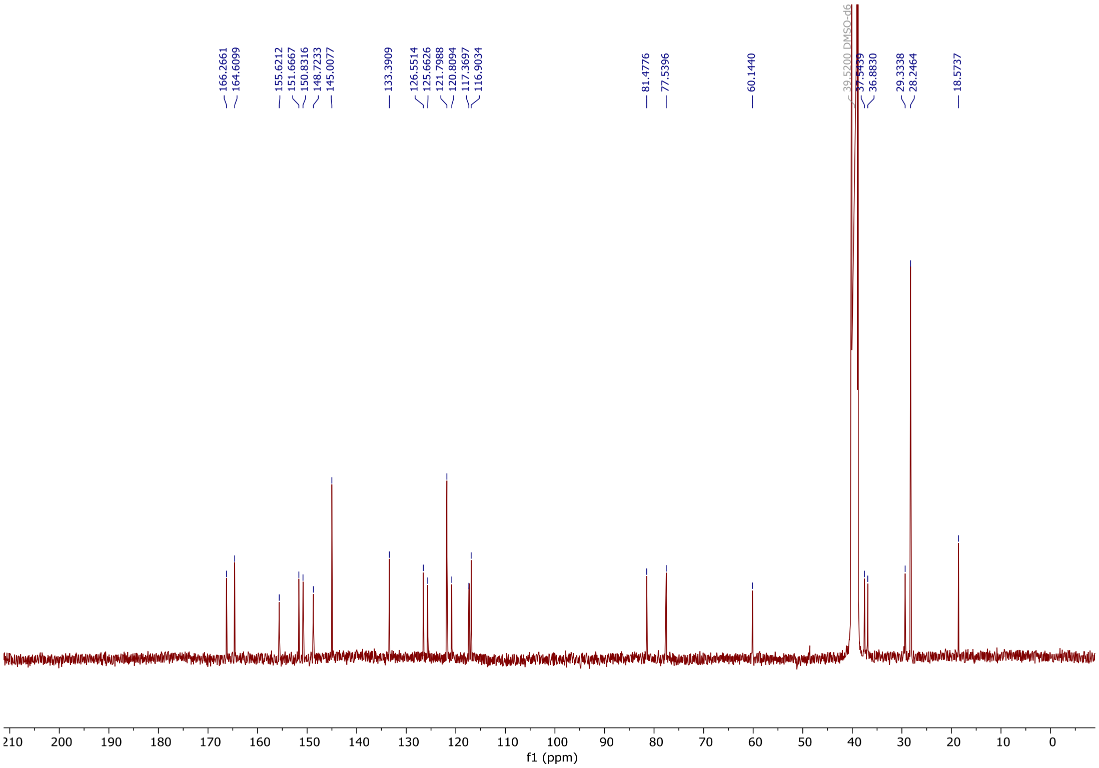

Figure S15. ^{1}H and ^{13}C NMR spectra of compound **9** in DMSO-d_6.

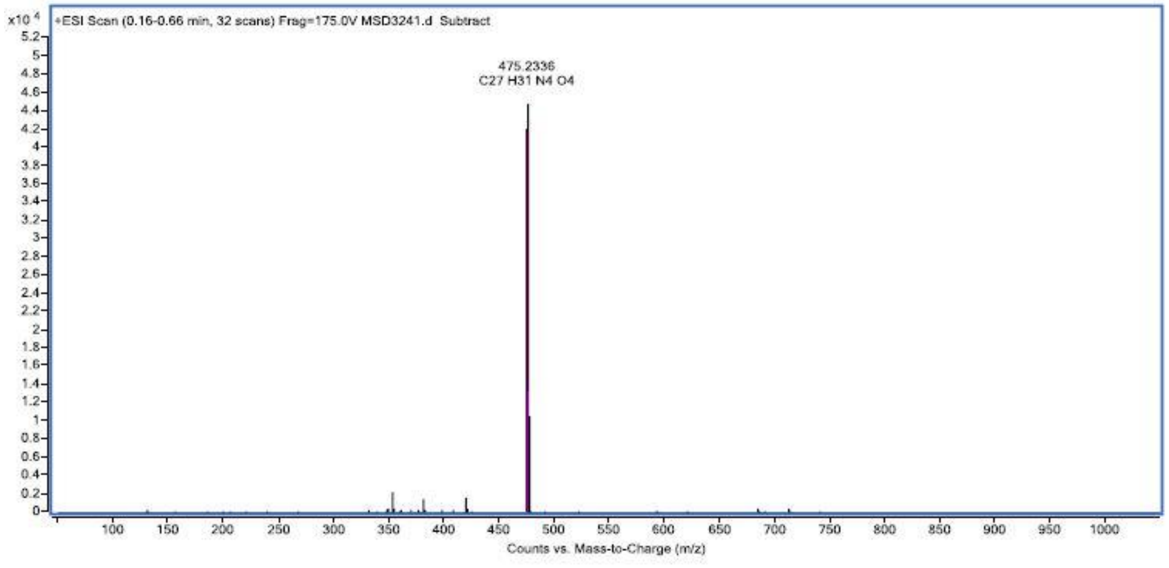

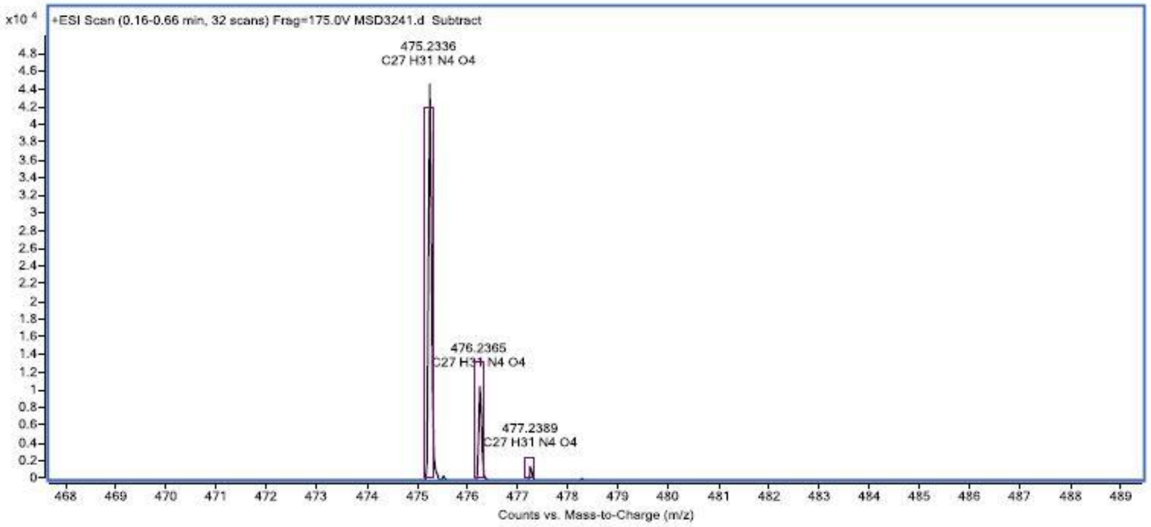

Figure S16. HR ESI-MS spectrum of compound **9**.

Compound 11

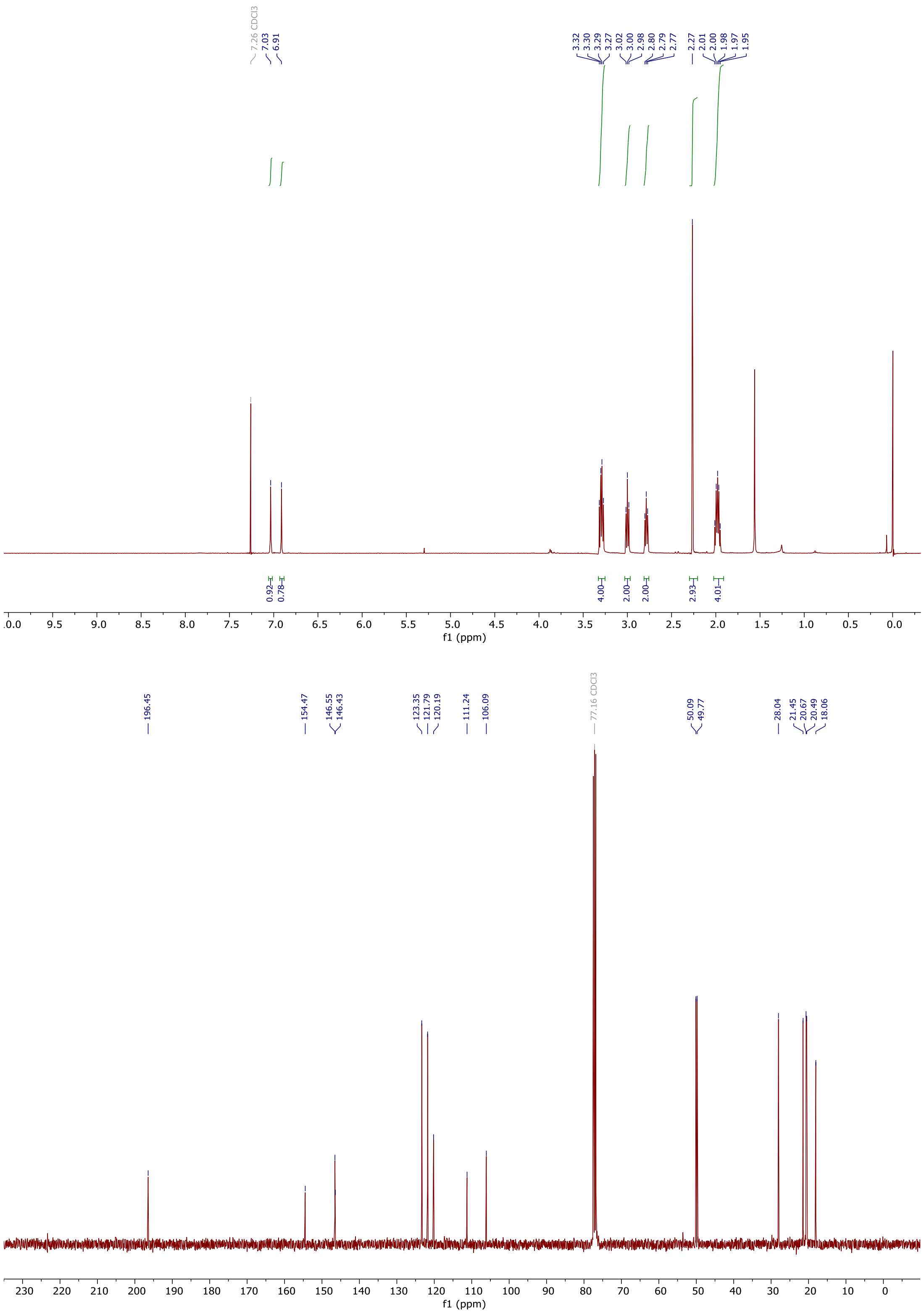

Figure S17. ^{1}H and ^{13}C NMR spectra of compound **11** in $CDCl_3$.

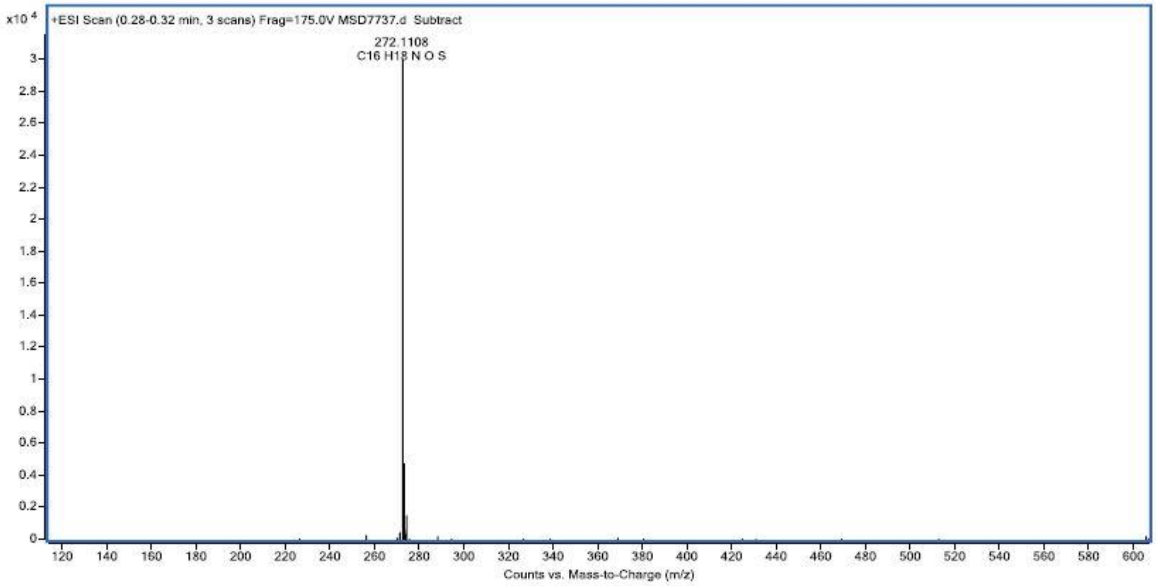

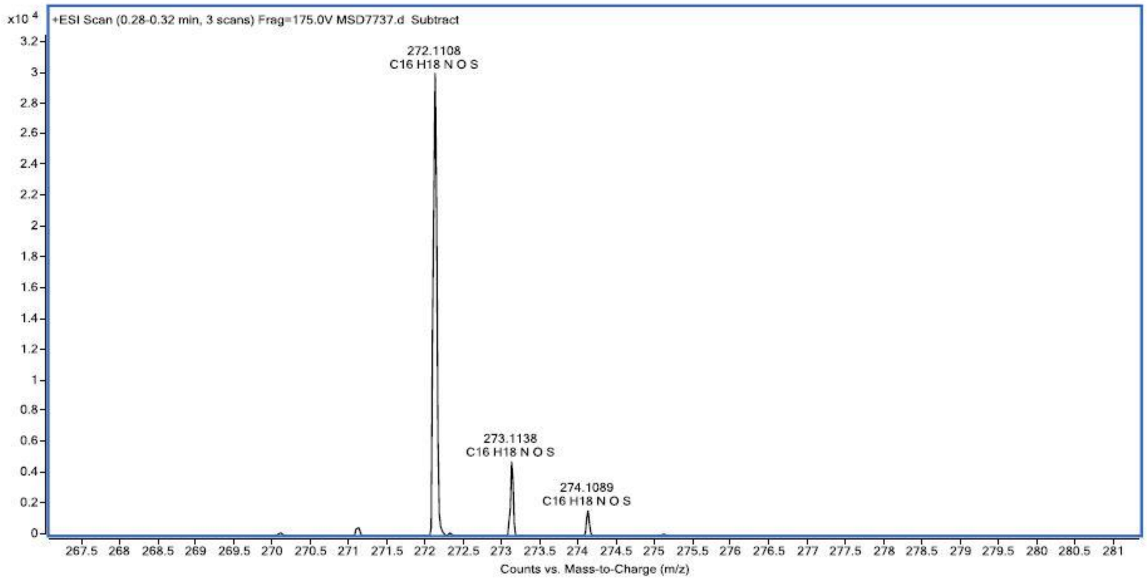

Figure S18. HR ESI-MS spectrum of compound **11**.

Compound 12

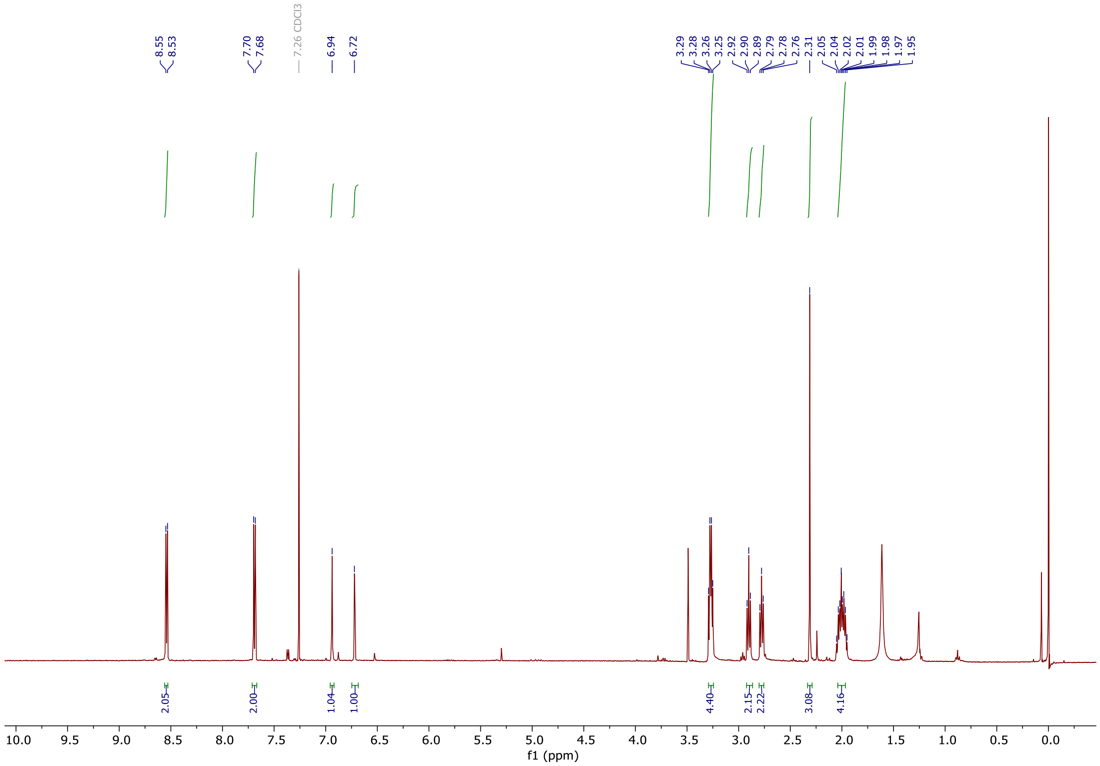

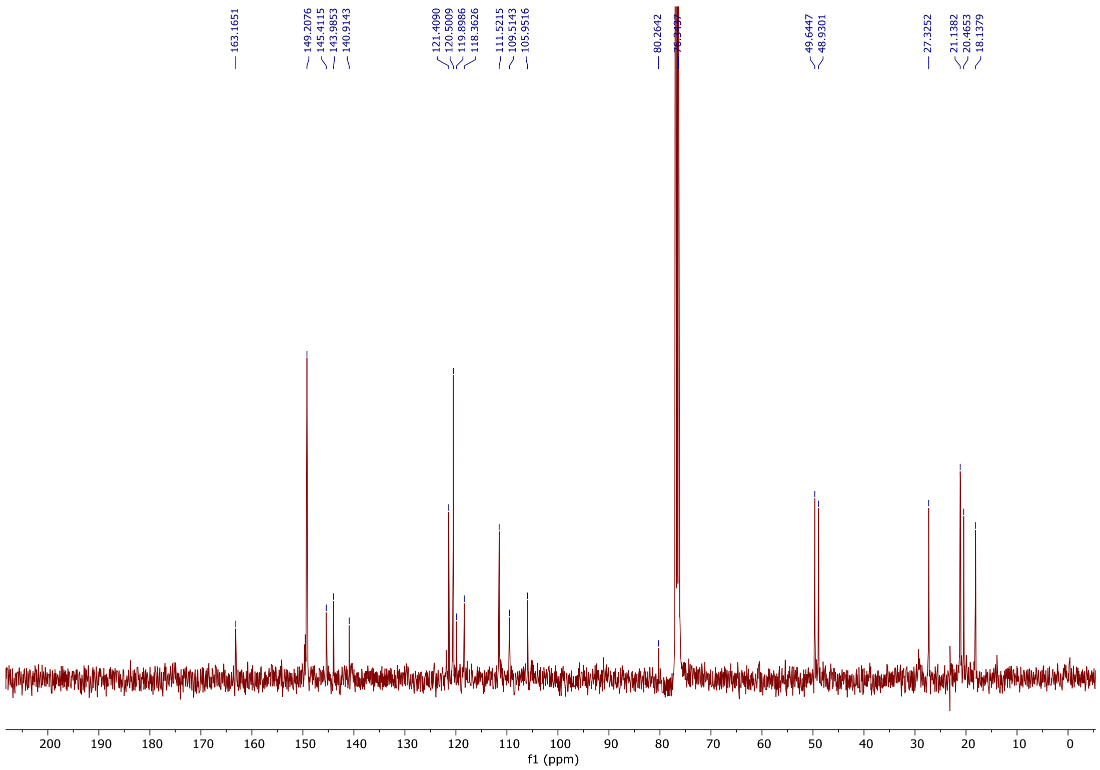

Figure S19. ^{1}H and ^{13}C NMR spectra of compound **12** in $CDCl_3$.

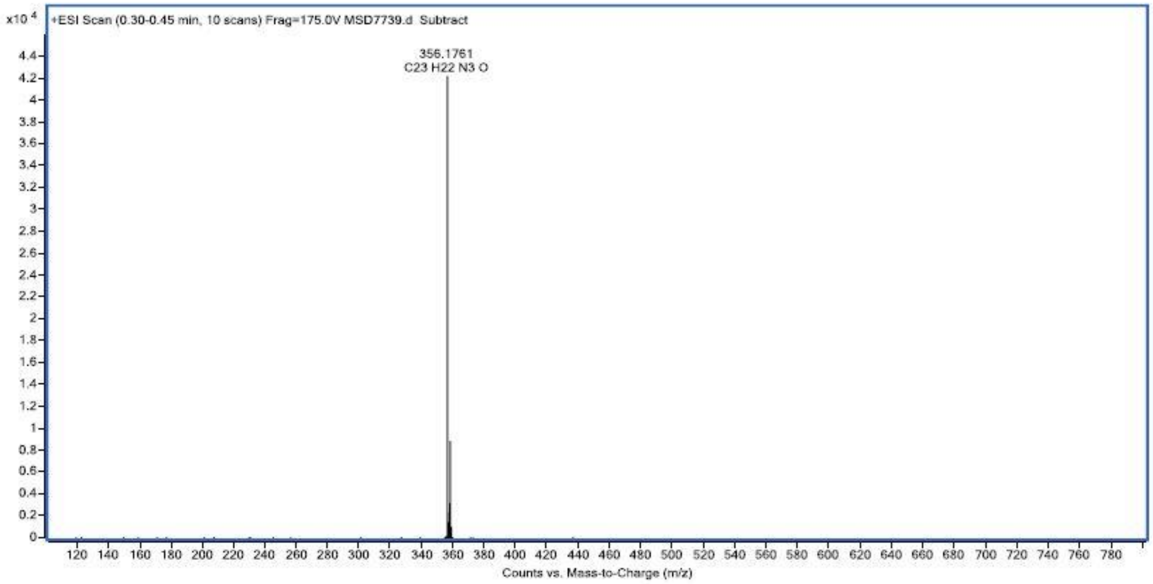

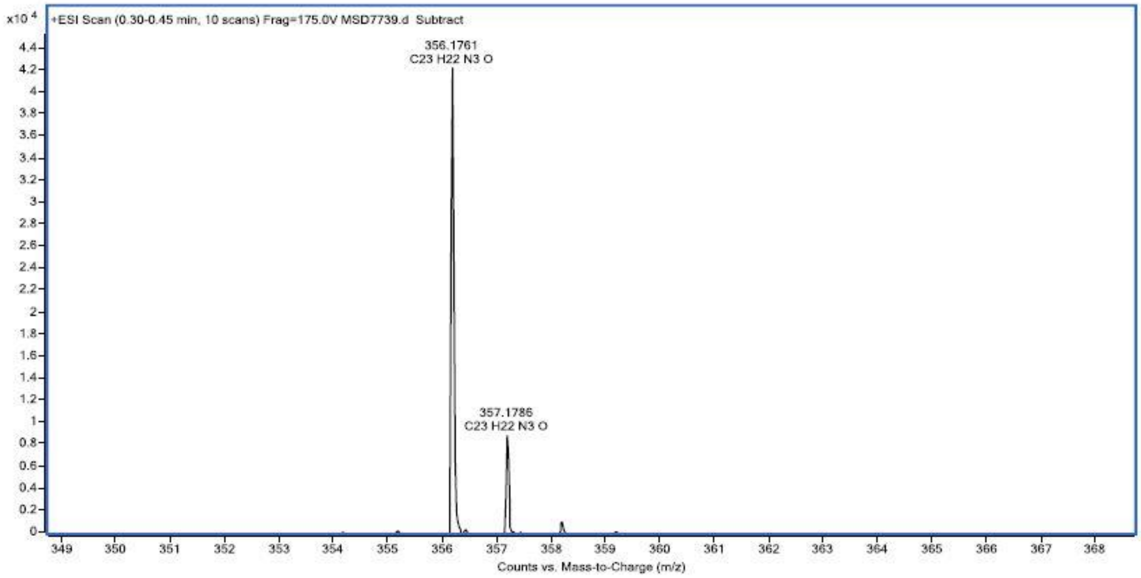

Figure S20. HR ESI-MS spectrum of compound **12**.

Compound 13

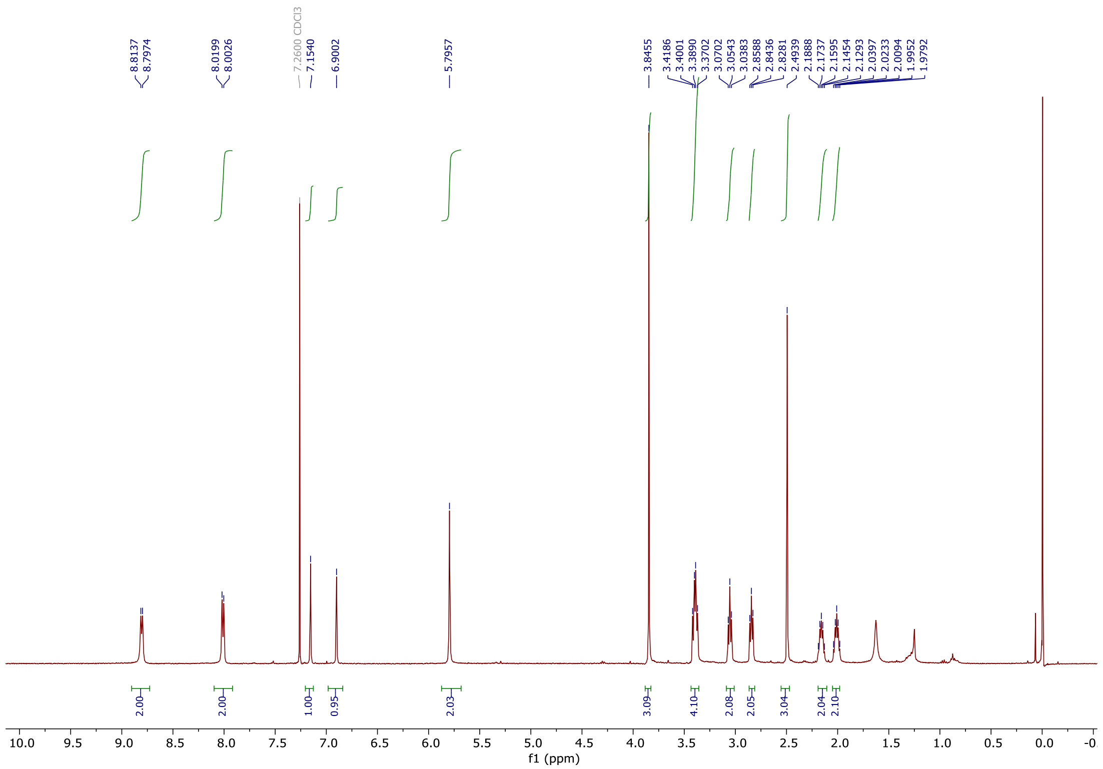

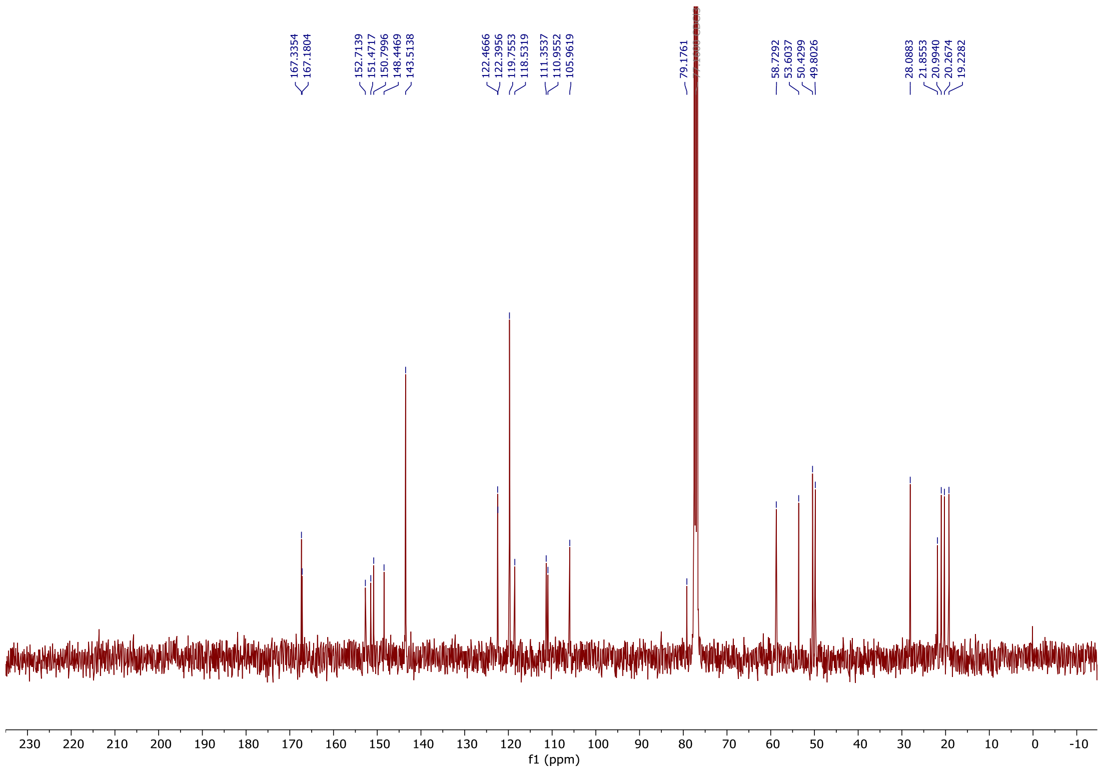

Figure S21. ^{1}H and ^{13}C NMR spectra of compound **13** in $CDCl_3$.

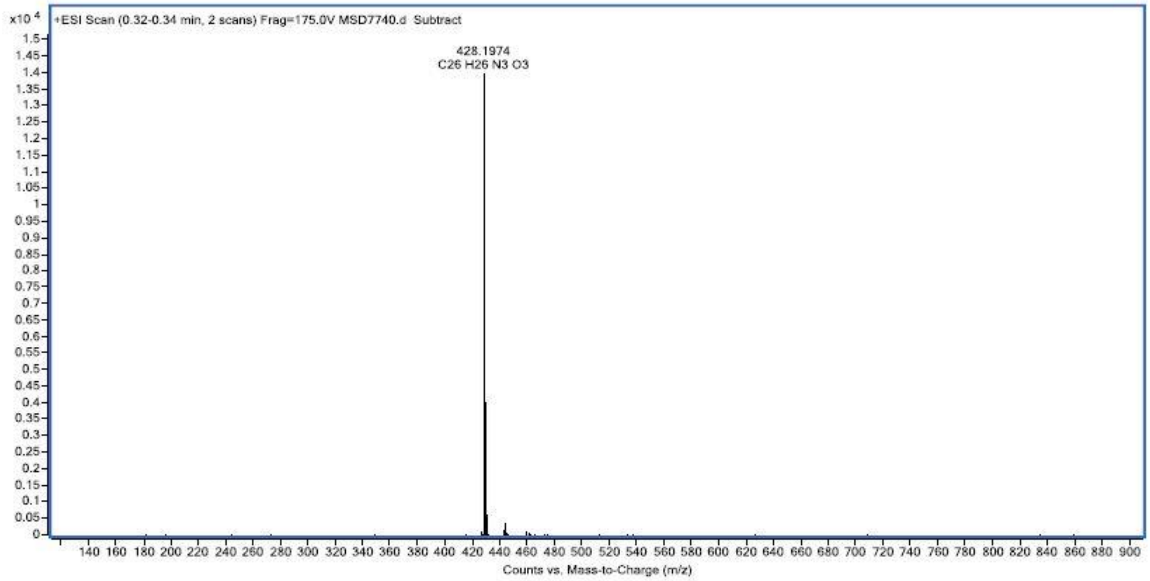

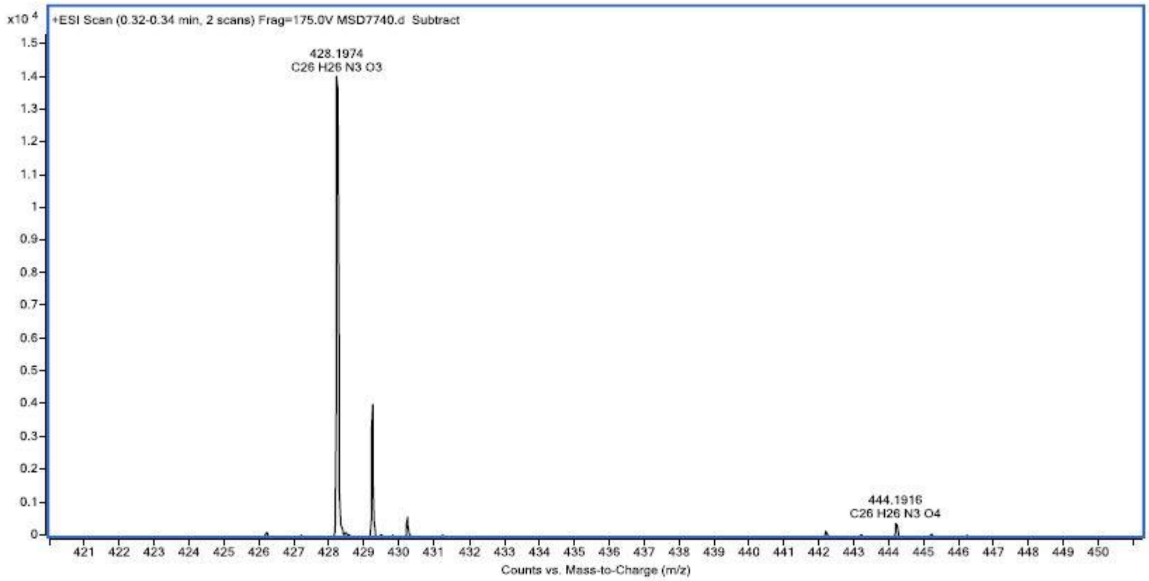

Figure S22. HR ESI-MS spectrum of compound **13**.

Compound 15

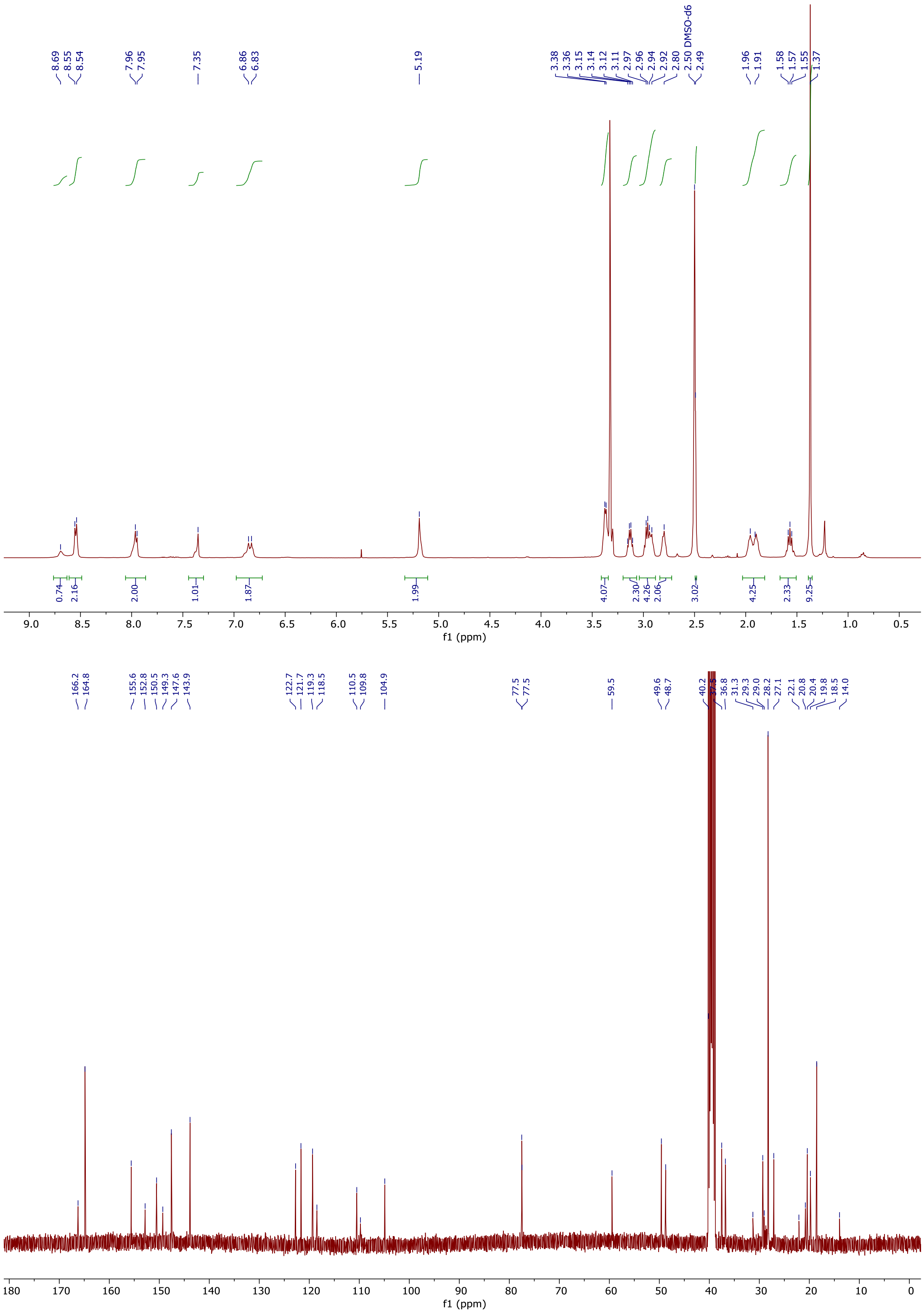

Figure S23. ^{1}H and ^{13}C NMR spectra of compound **15** in $CDCl_3$.

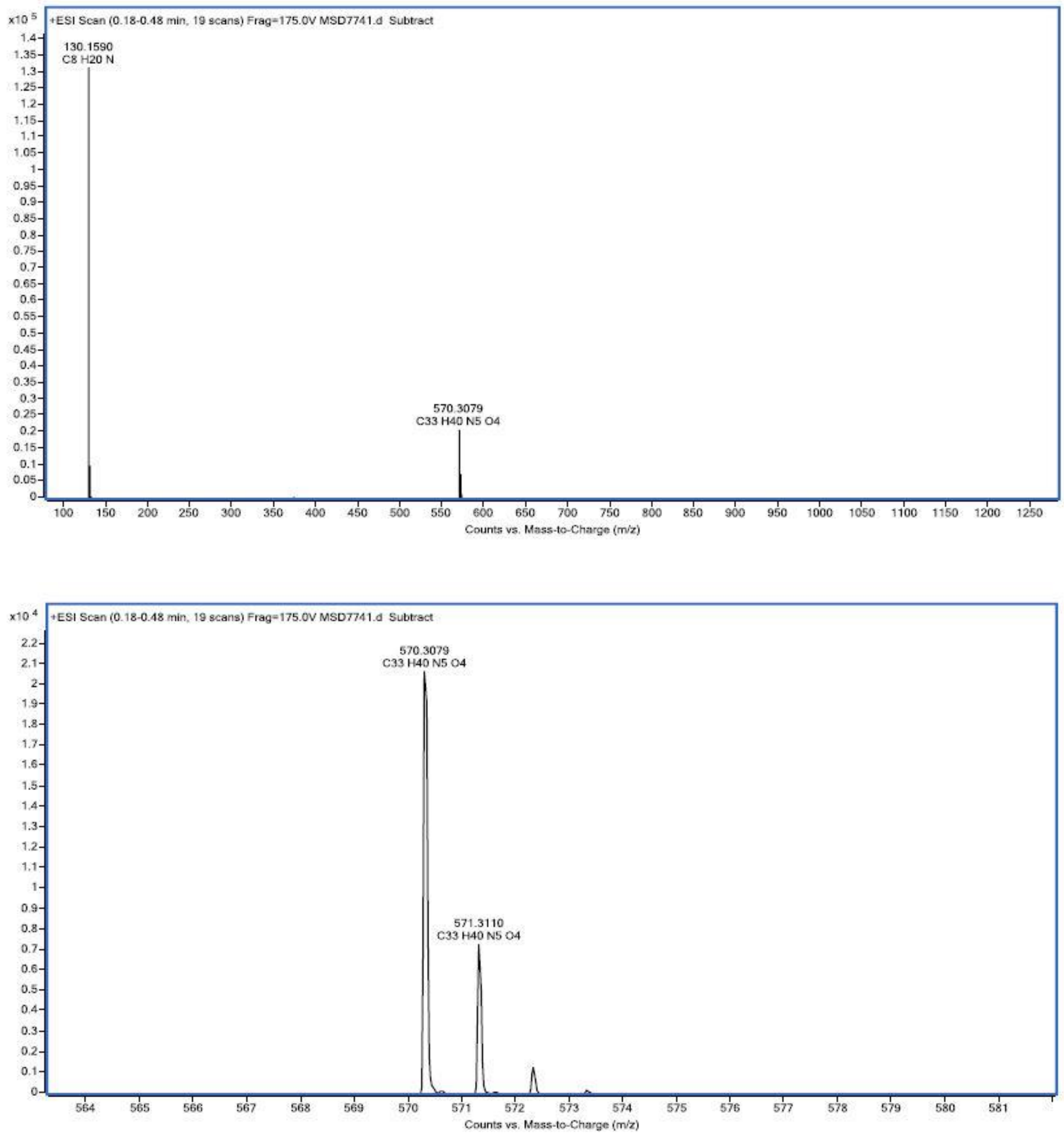

Figure S24. HR ESI-MS spectrum of compound **15**.

Compound 18

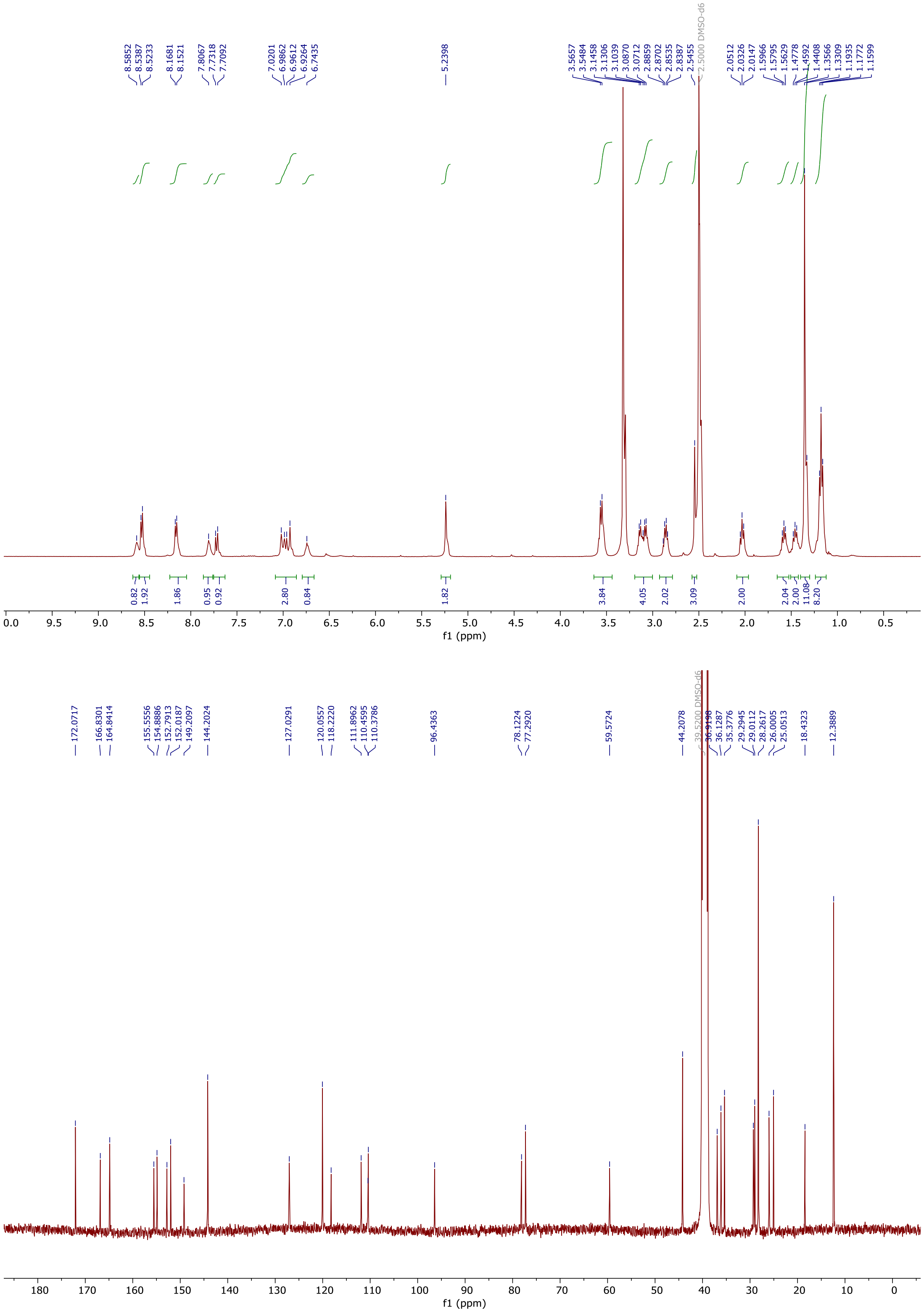

Figure S25. ^{1}H and ^{13}C NMR spectra of compound **18** in DMSO-d_6.

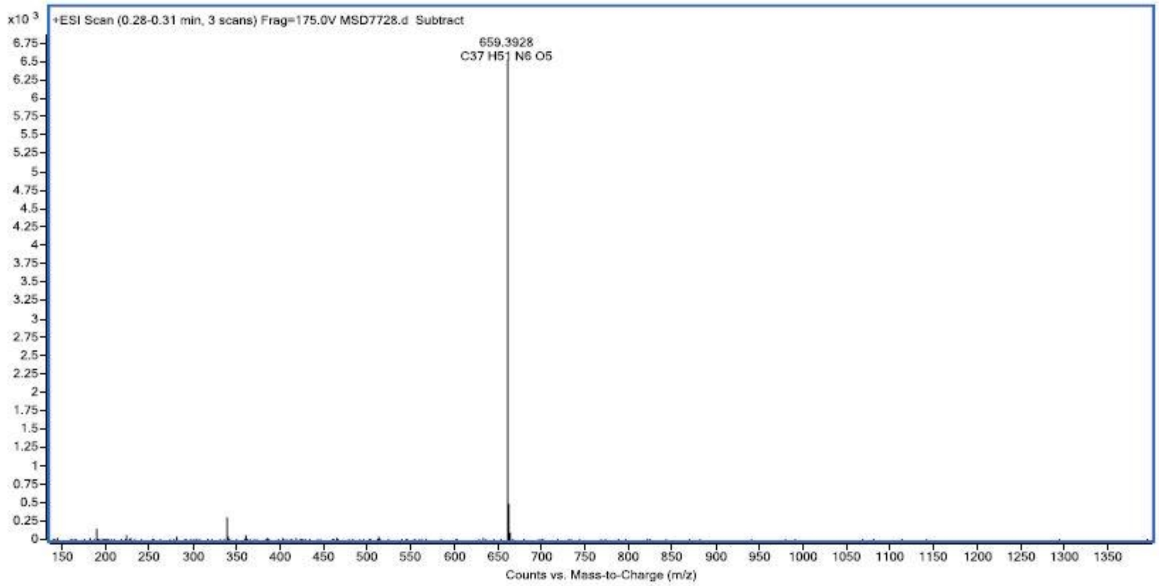

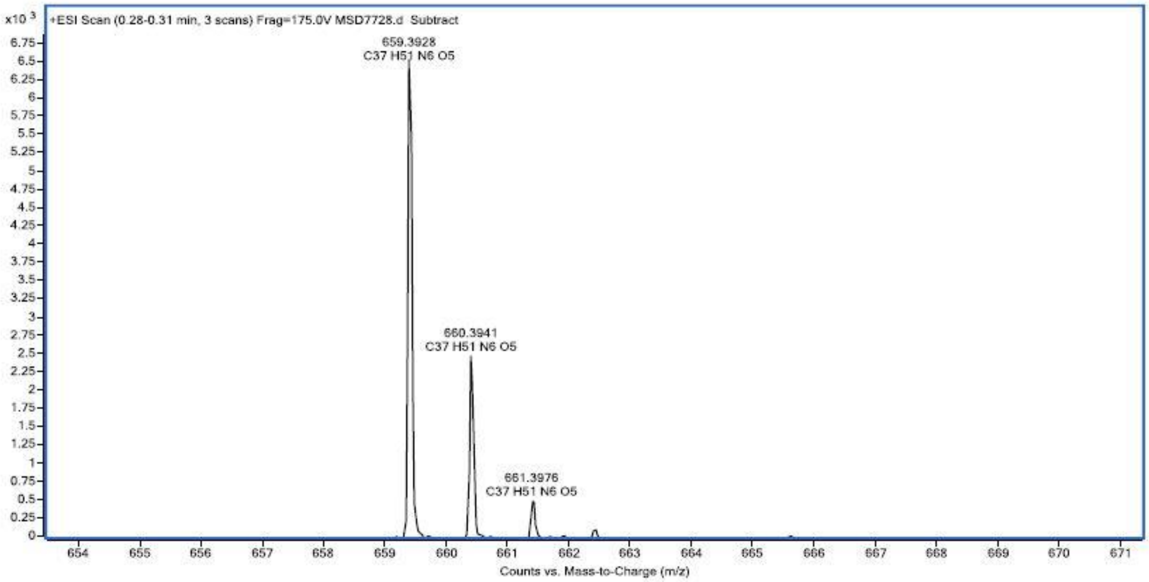

Figure S26. HR ESI-MS spectrum of compound **18**

Compound 19

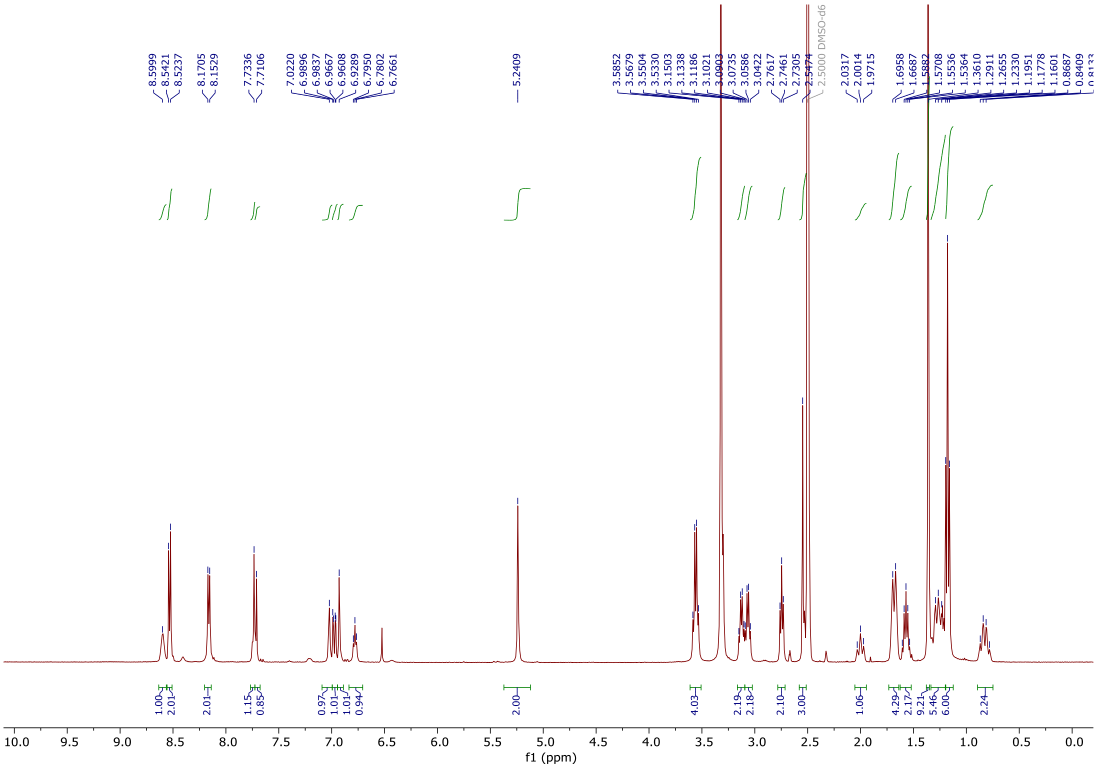

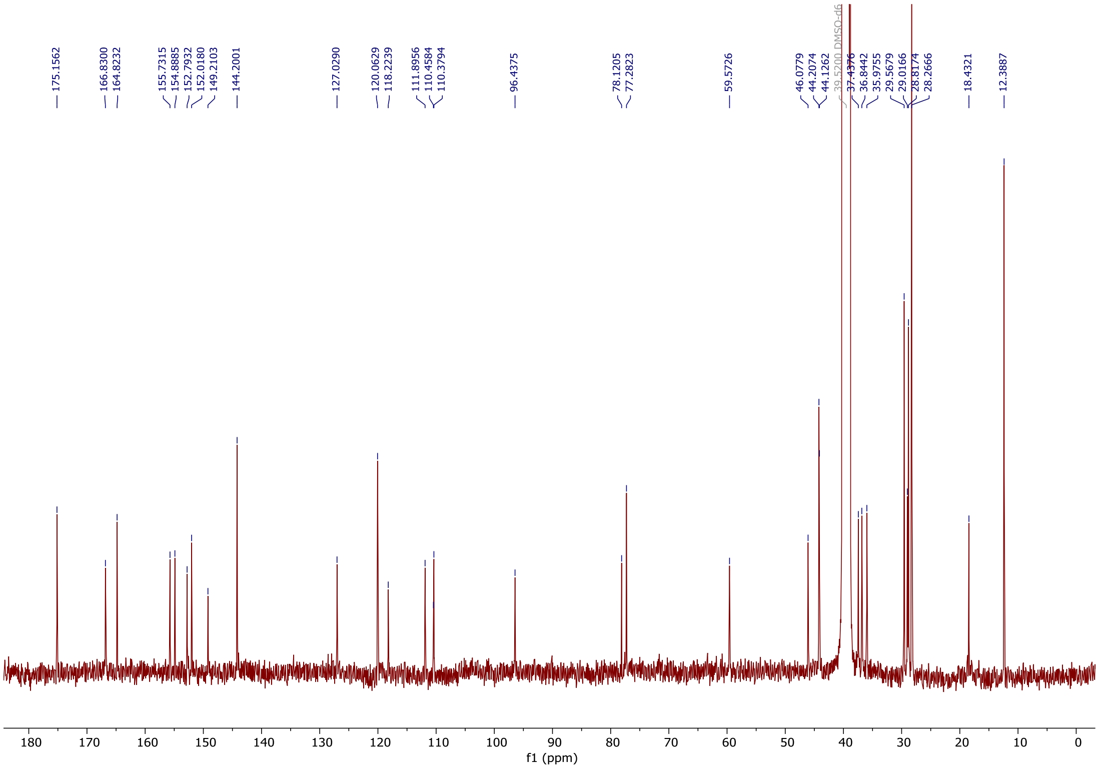

Figure S27. ^{1}H and ^{13}C NMR spectra of compound **19** in DMSO-d_6.

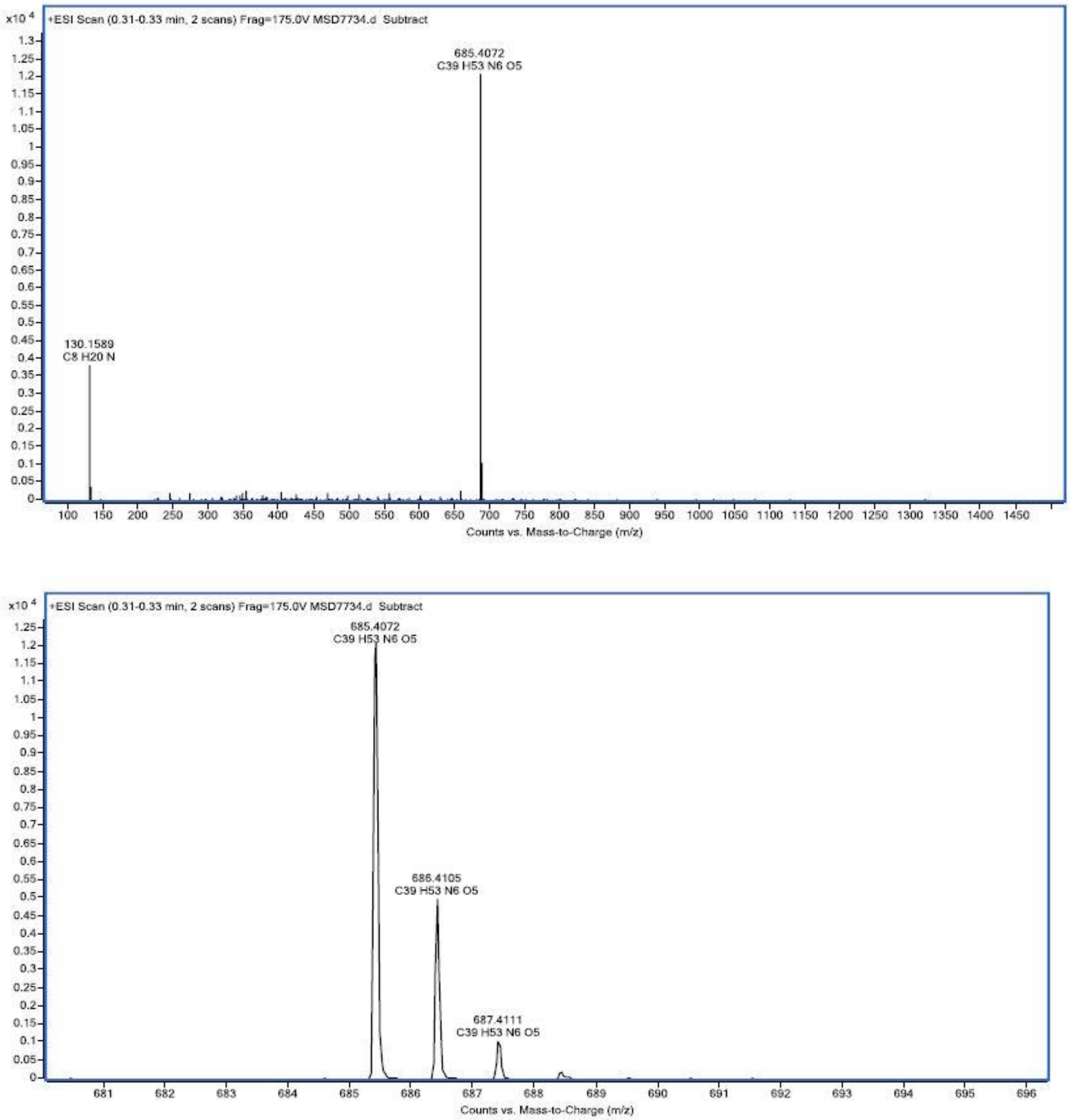

Figure S28. HR ESI-MS spectrum of compound **19**

COUPY 1b

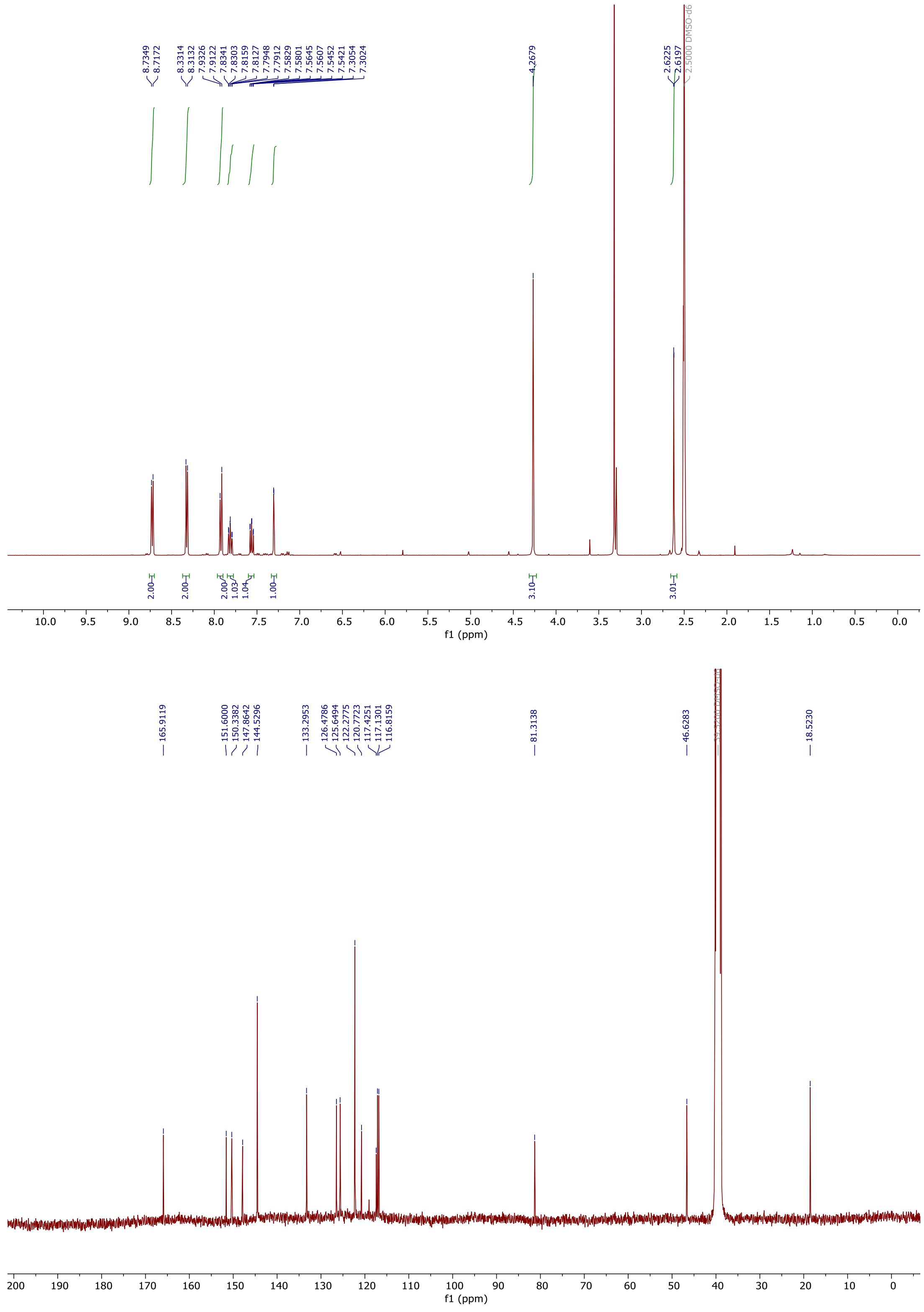

Figure S29. ^{1}H and ^{13}C NMR spectra of compound **1b** in DMSO-d_6.

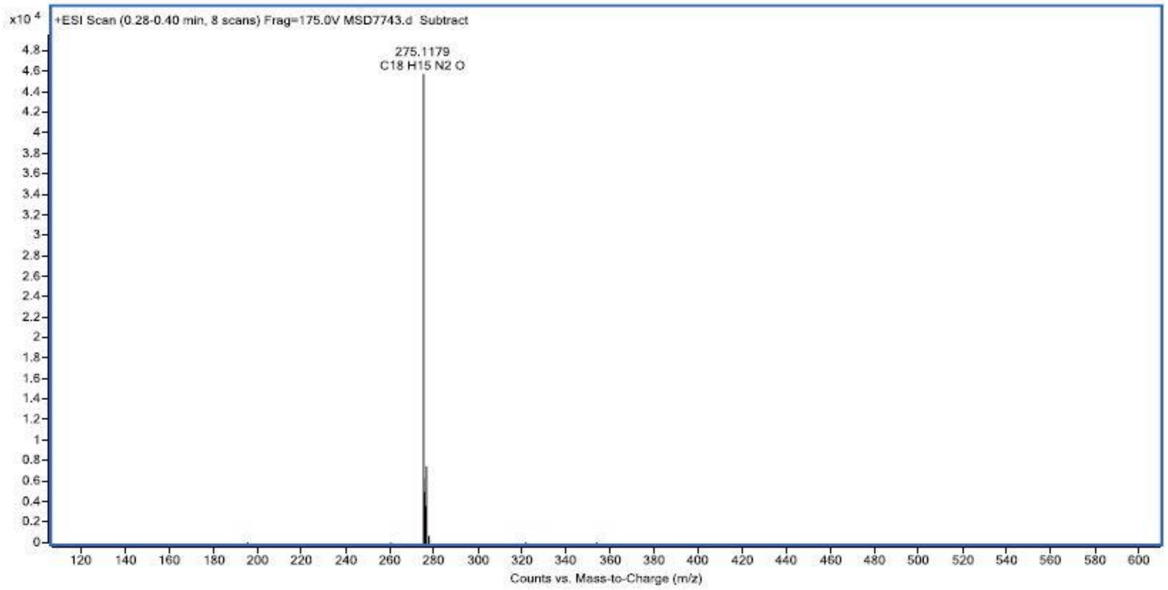

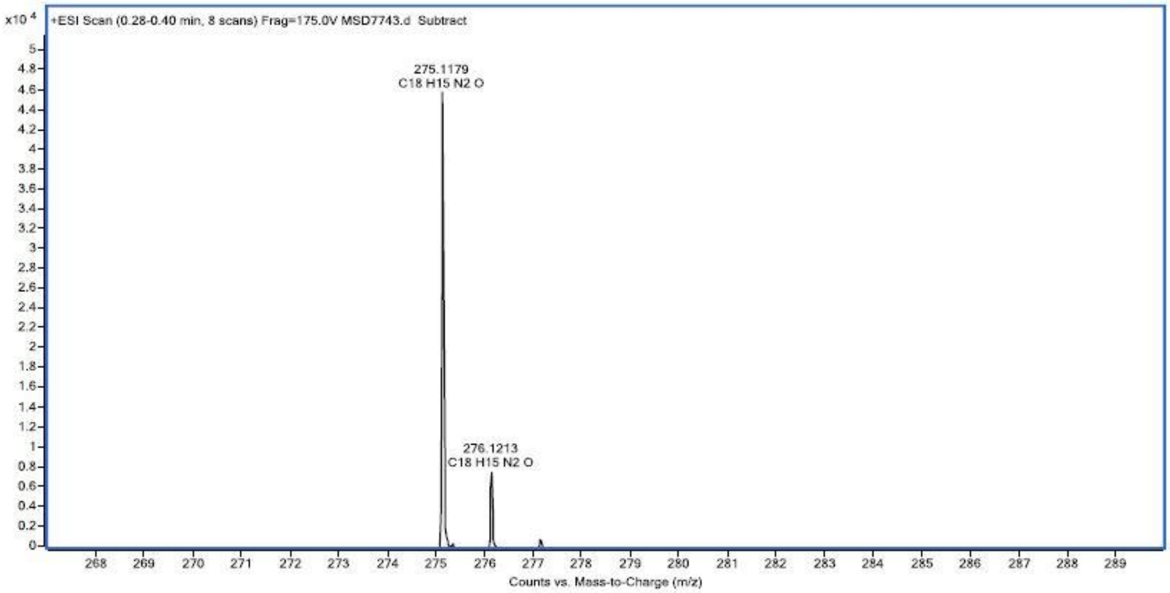

Figure S30. HR ESI-MS spectrum of compound **1b.**

COUPY 1c

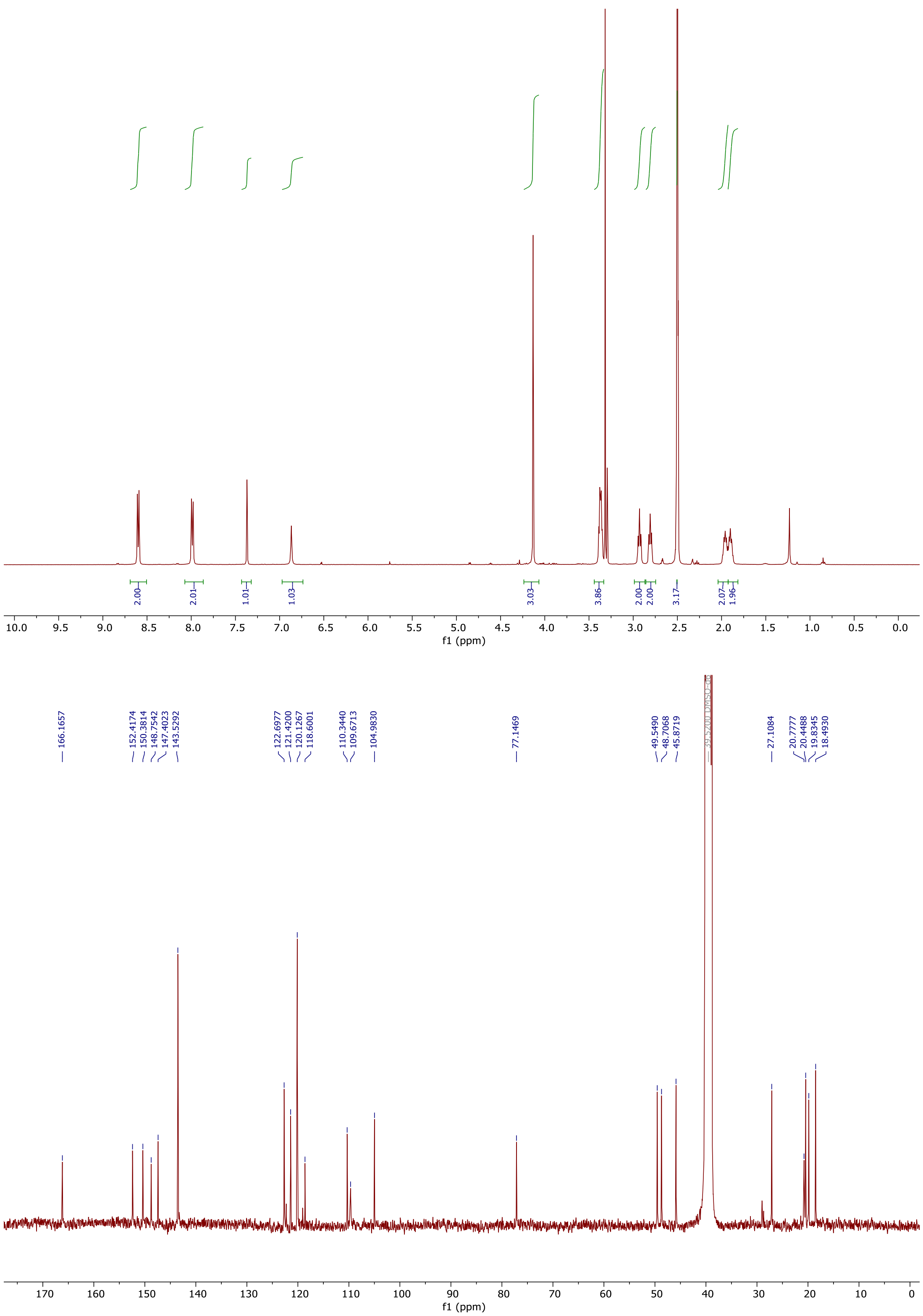

Figure S31. ^{1}H and ^{13}C NMR spectra of compound **1c** in DMSO-d_6.

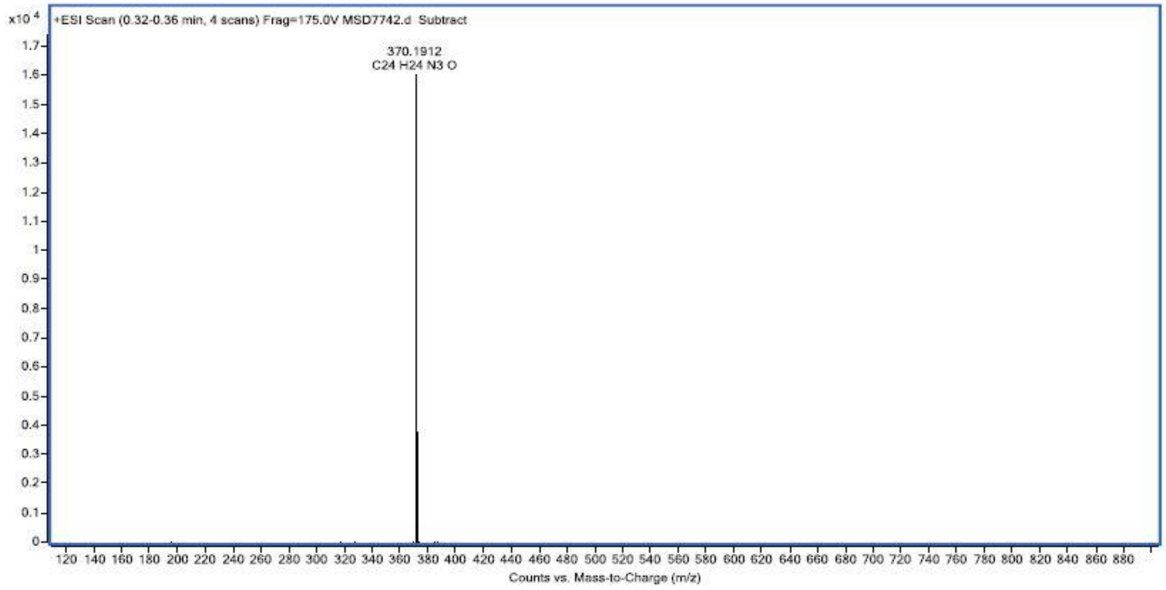

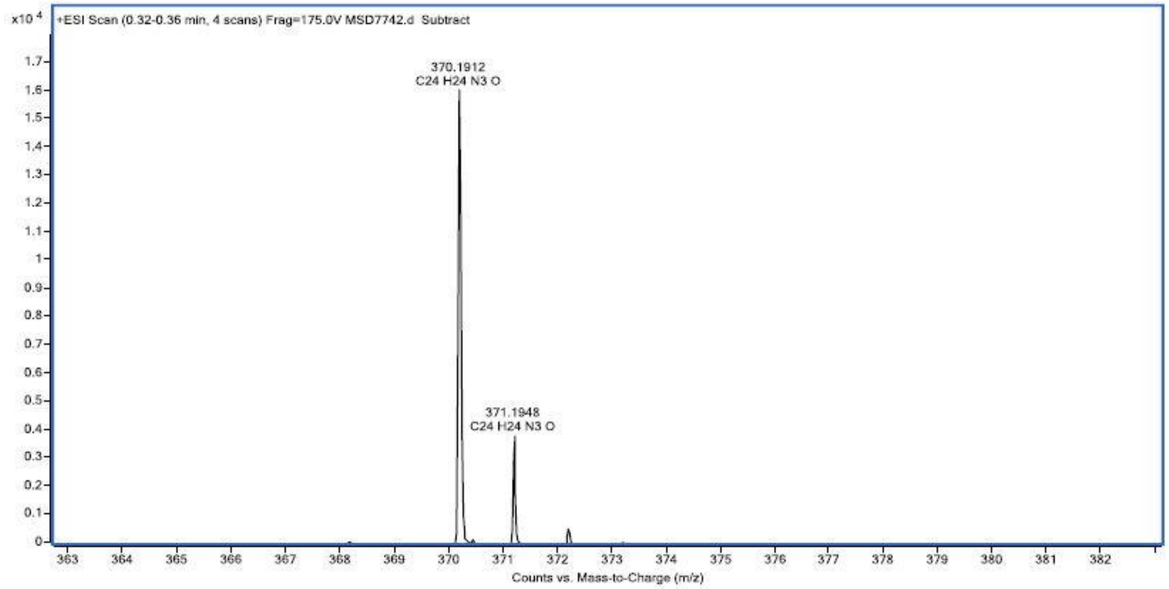

Figure S32. HR ESI-MS spectrum of compound **1c.**

Ir(III)-COUPY conjugate 3b

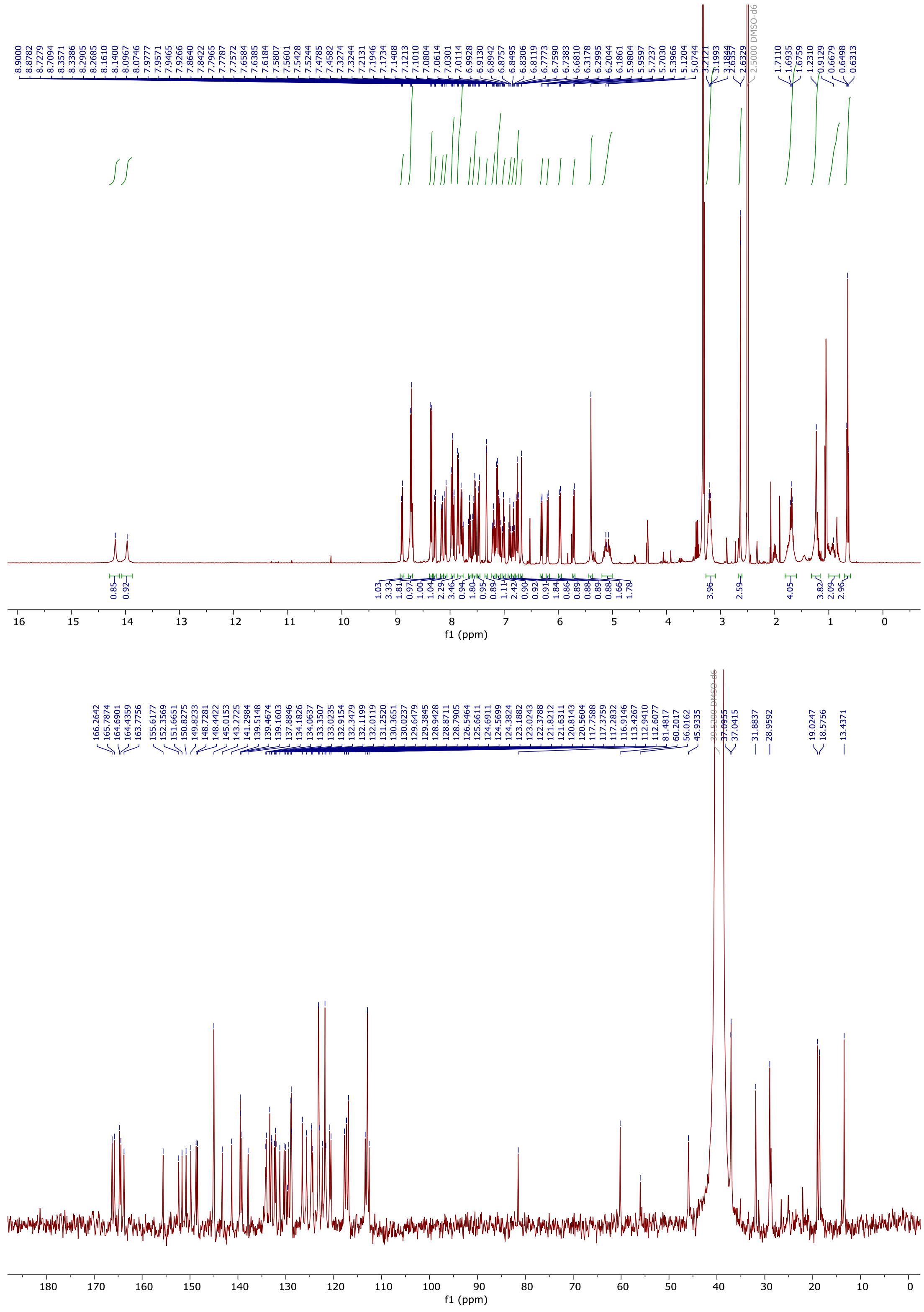

Figure S33. ^{1}H and ^{13}C NMR spectra of compound **3b** in DMSO-d_6.

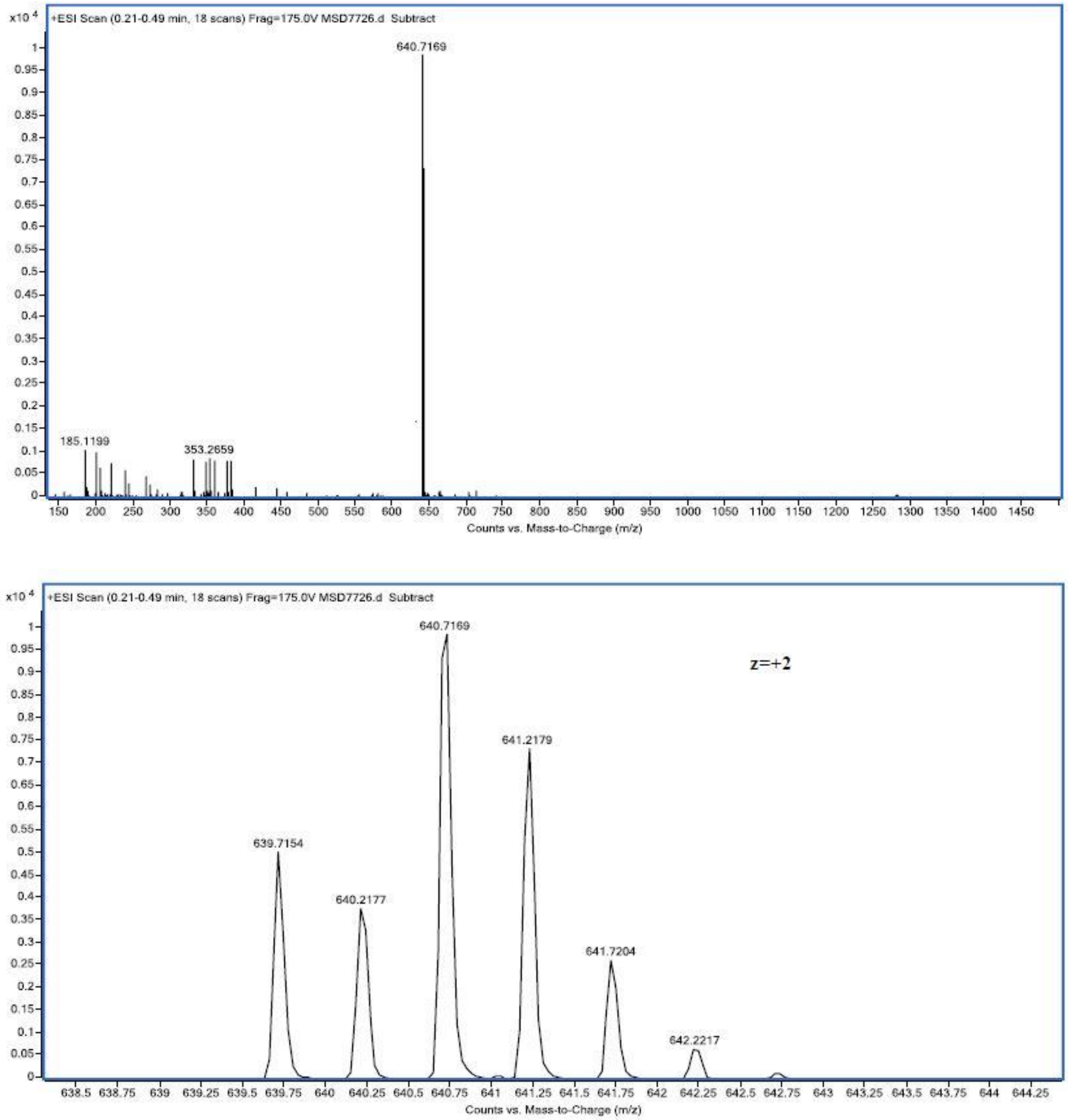

Figure S34. HR ESI-MS spectrum of compound **3b**

Ir(III)-COUPY conjugate 3c

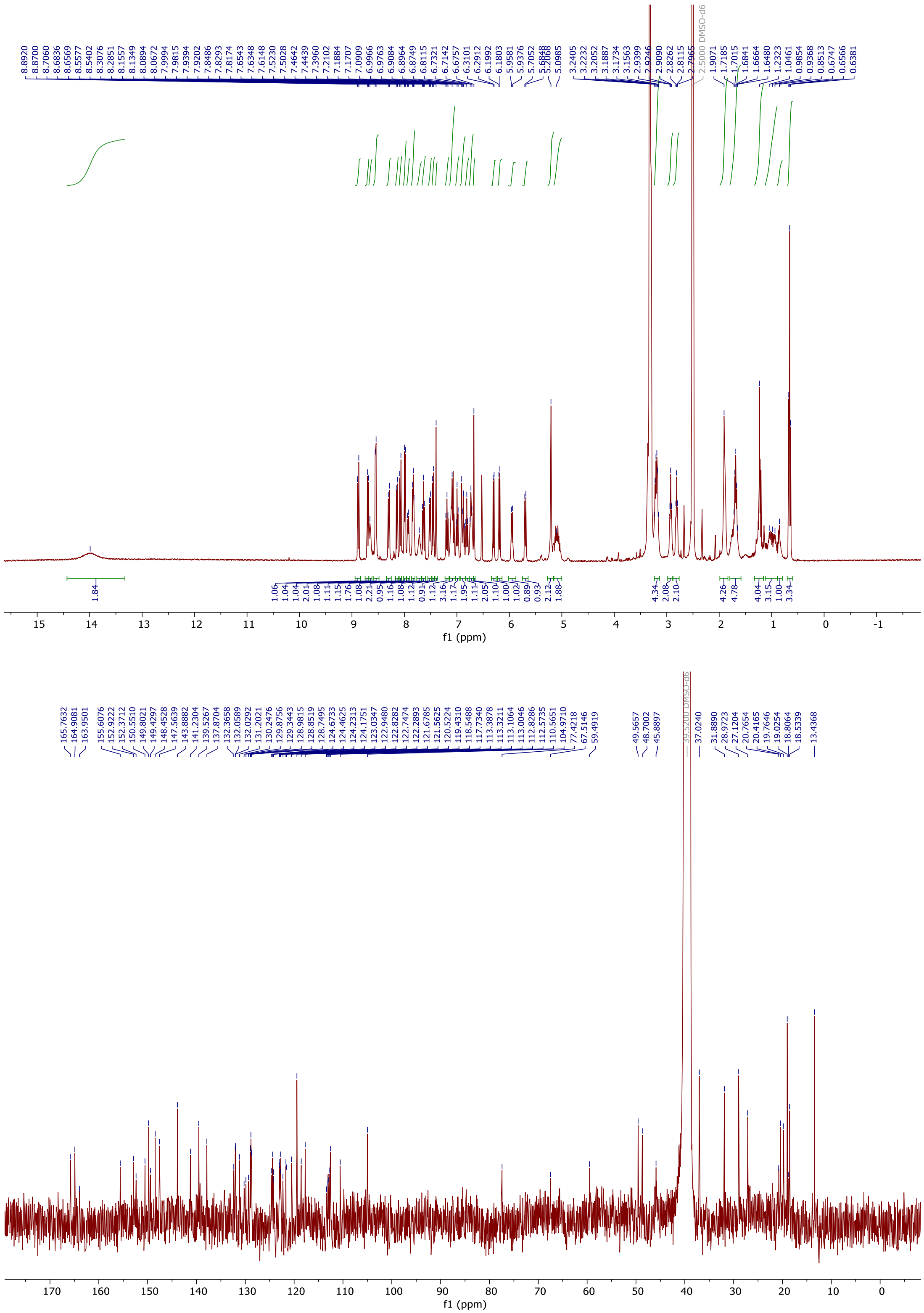

Figure S35. ^{1}H and ^{13}C NMR spectra of compound **3c** in DMSO-d_6.

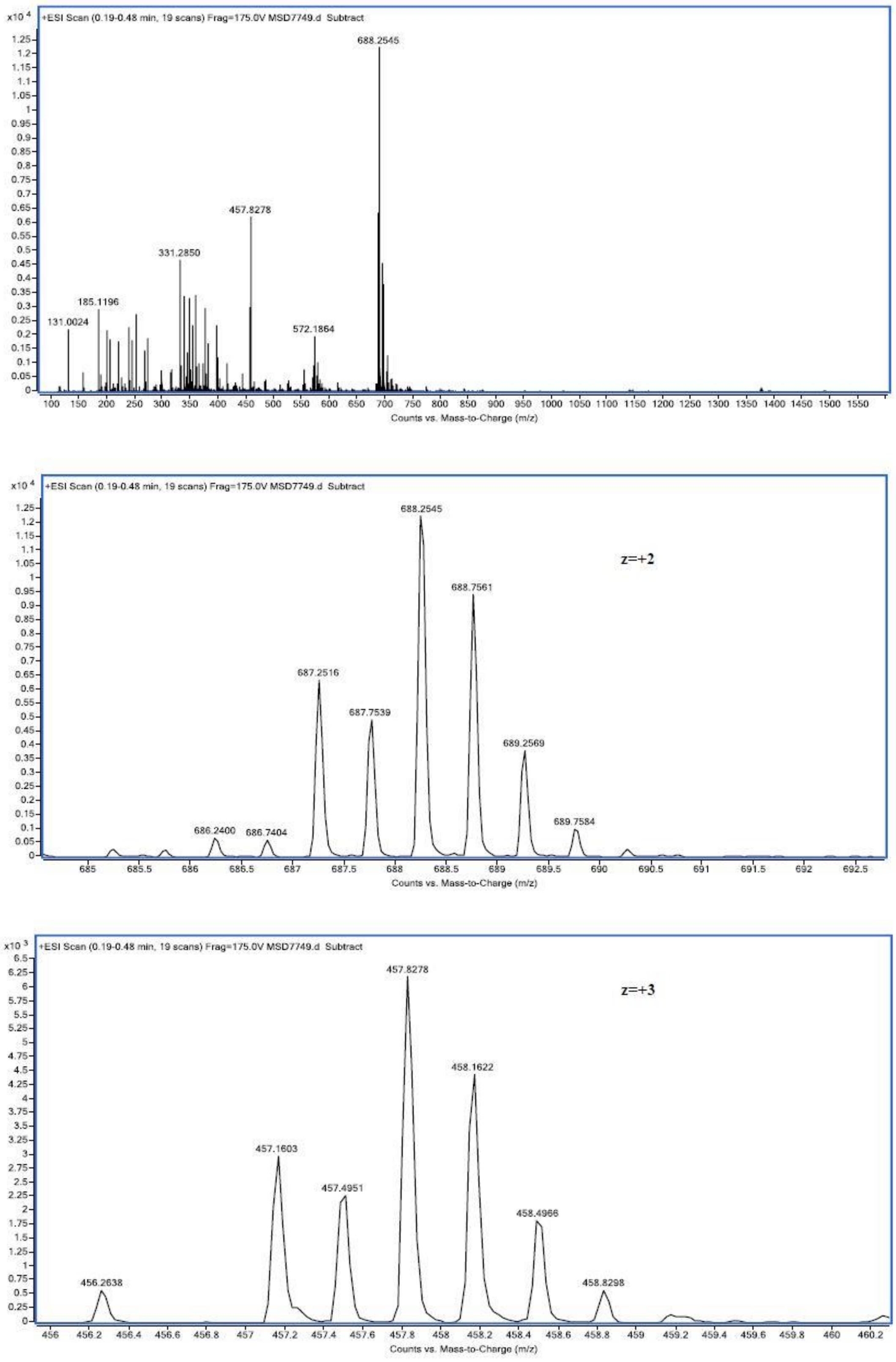

Figure S36. HR ESI-MS spectrum of compound **3c**

Ir(III)-COUPY conjugate 3d

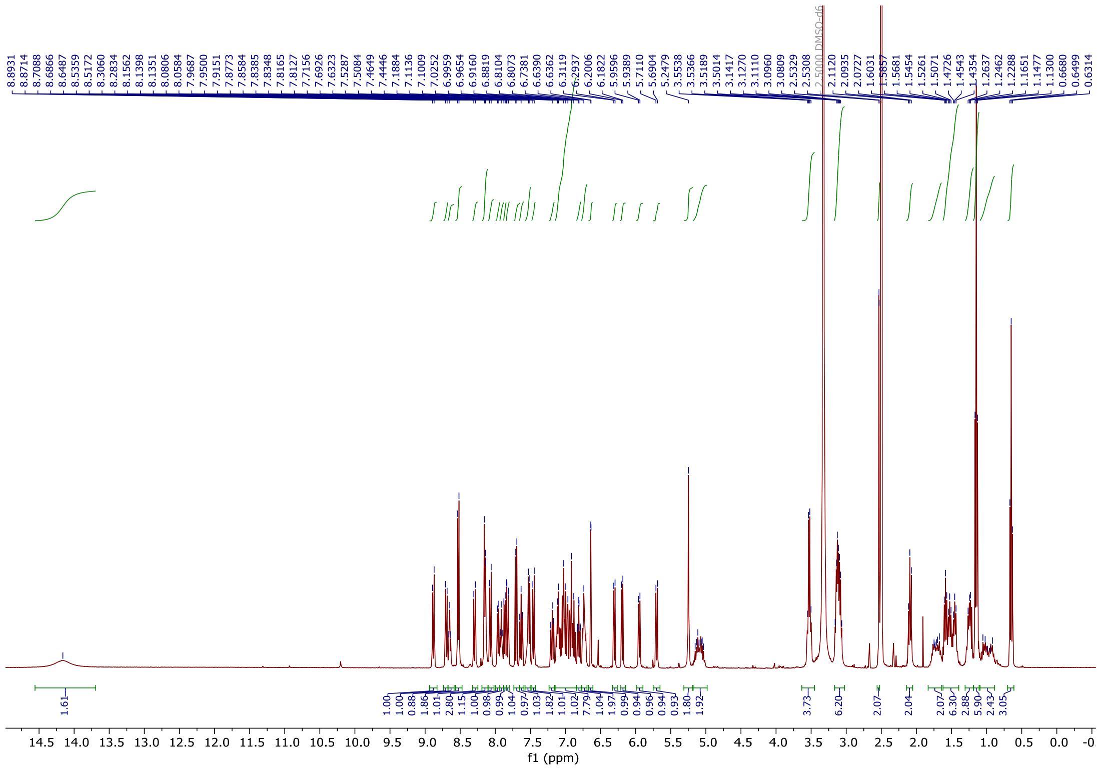

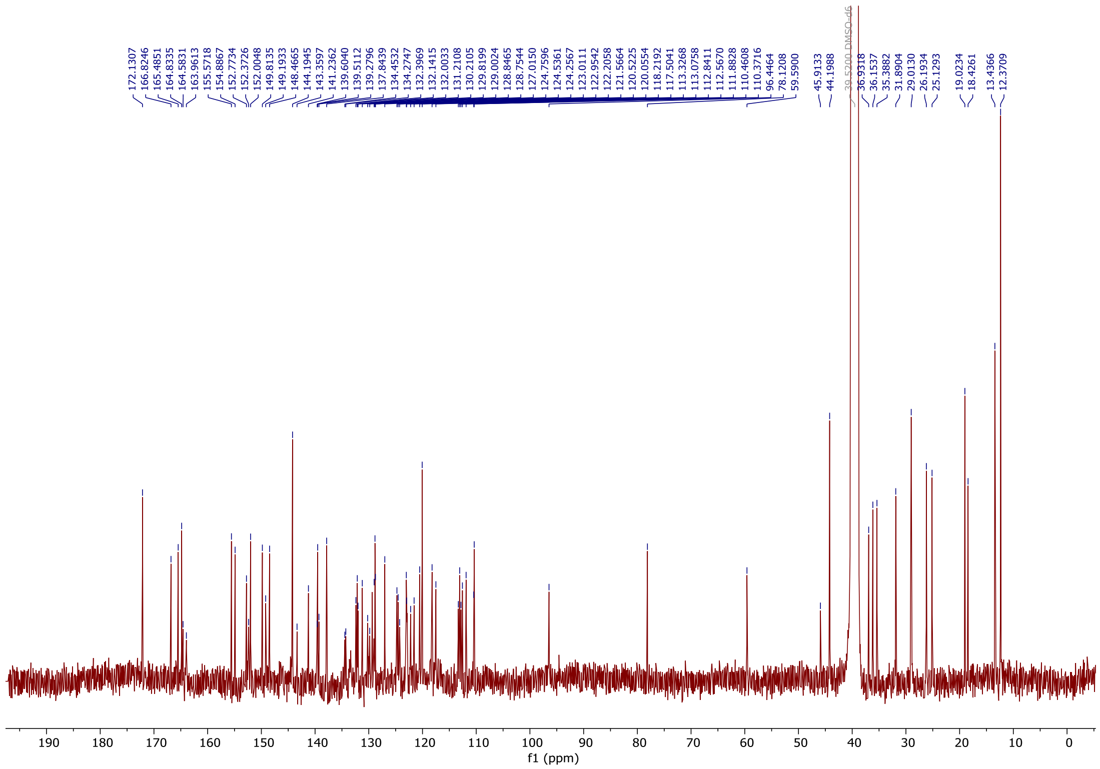

Figure S37. ^{1}H and ^{13}C NMR spectra of compound **3d** in DMSO-d_6.

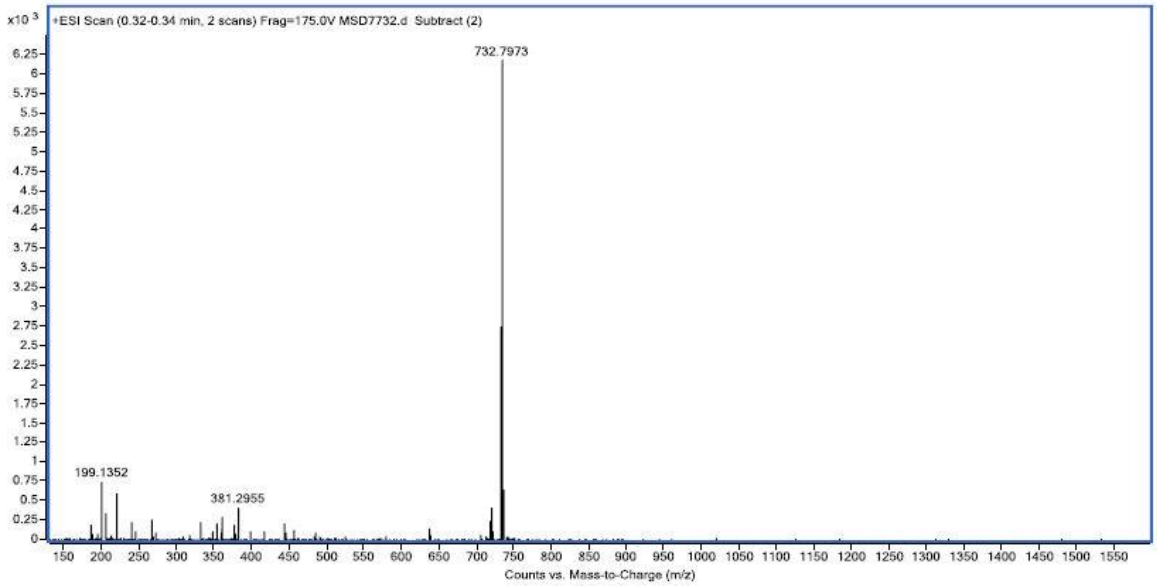

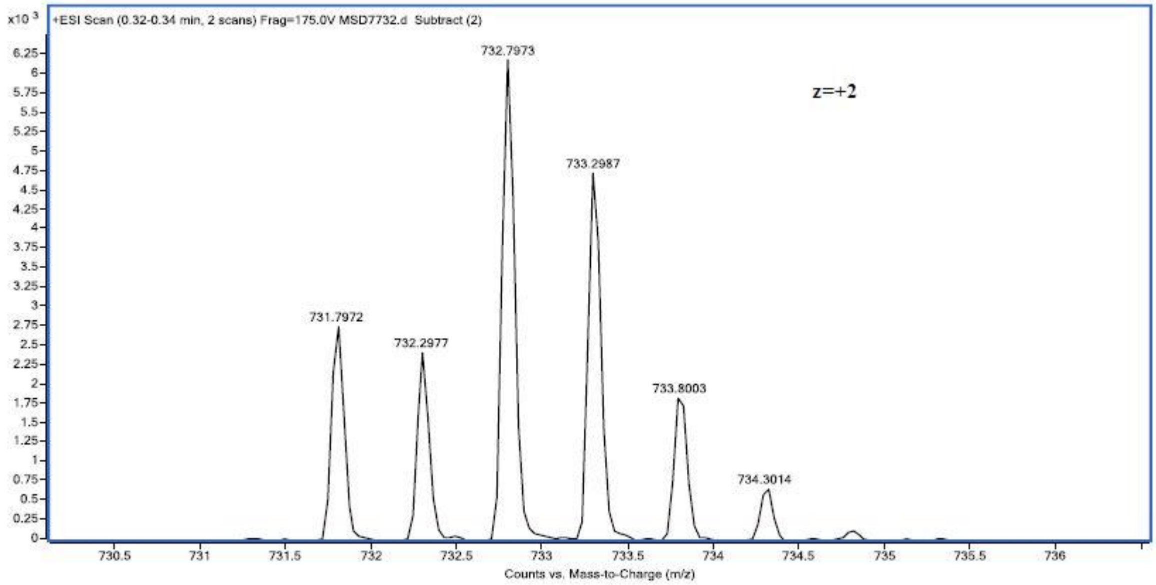

Figure S38. HR ESI-MS spectrum of compound **3d**

Ir(III)-COUPY conjugate 3e

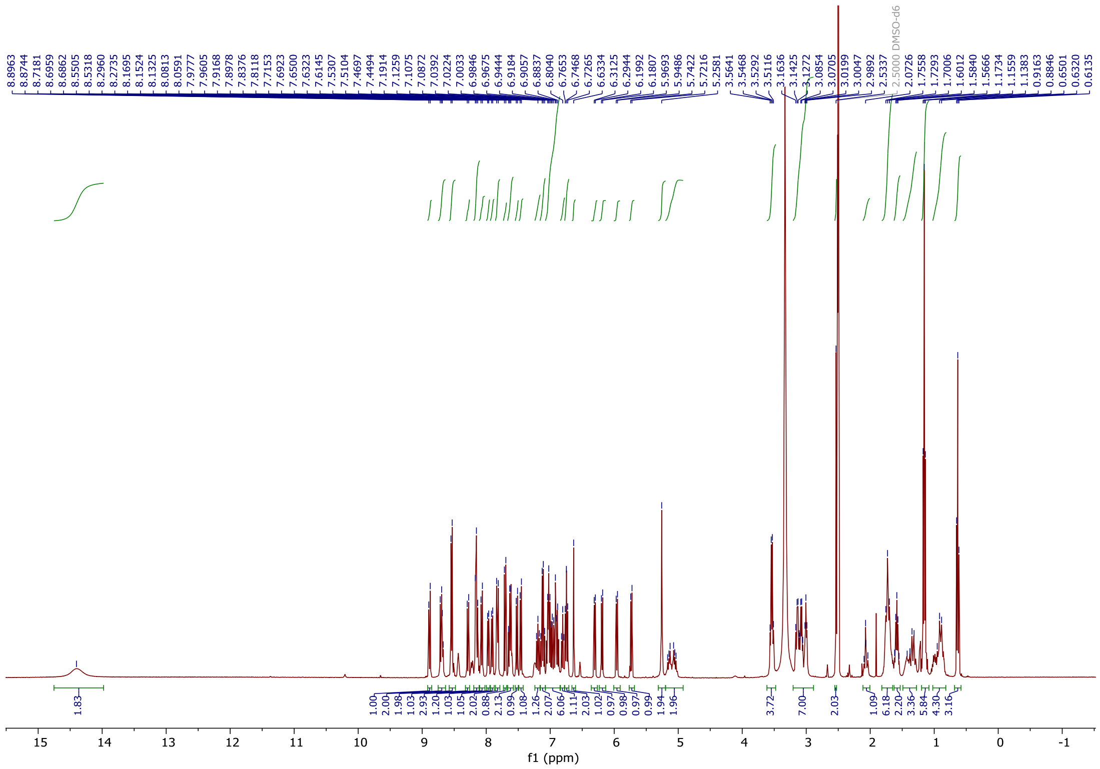

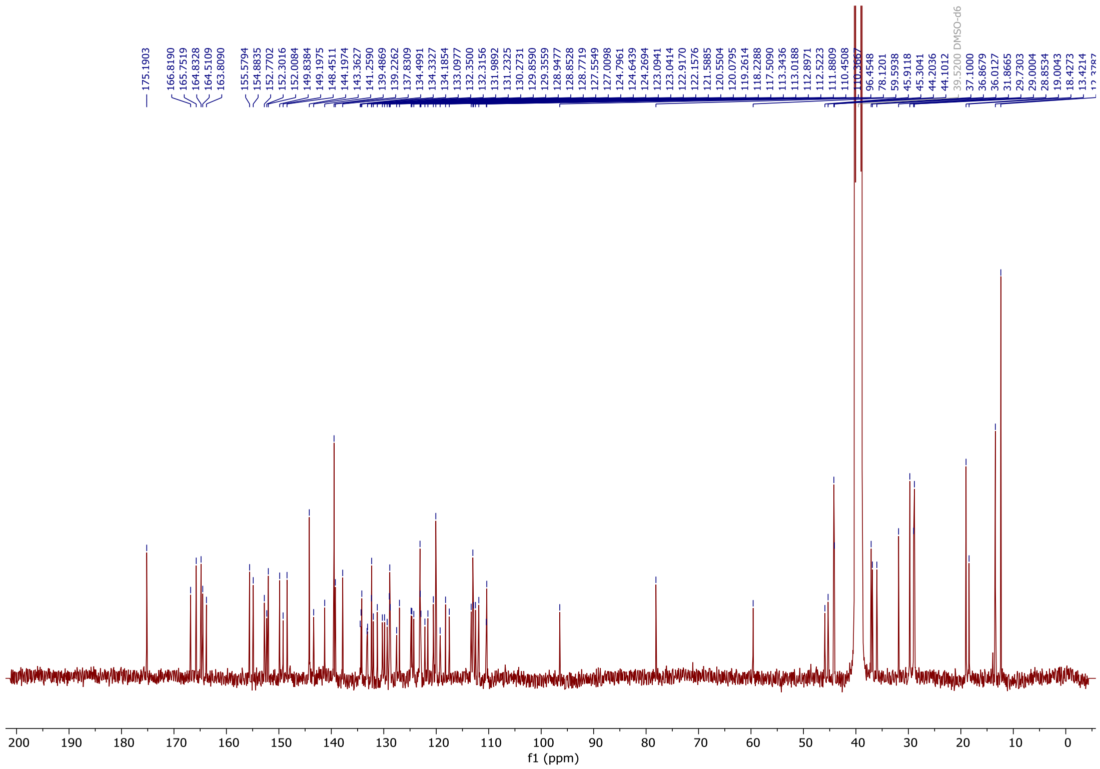

Figure S39. ^{1}H and ^{13}C NMR spectra of compound **3e** in DMSO-d_6.

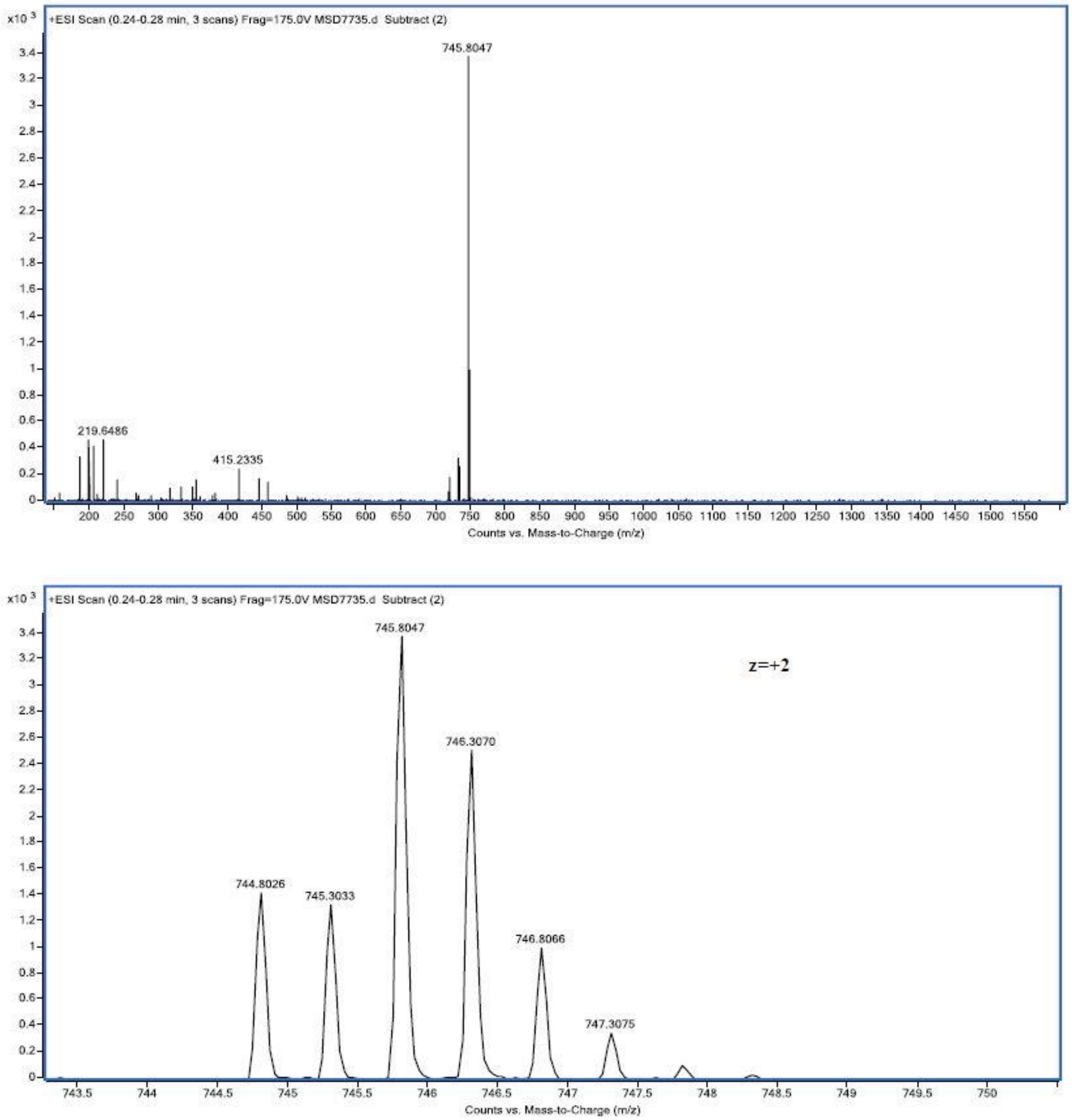

Figure S40. HR ESI-MS spectrum of compound **3e**

4. HPLC analysis of the compounds

COUPY **1a**<br>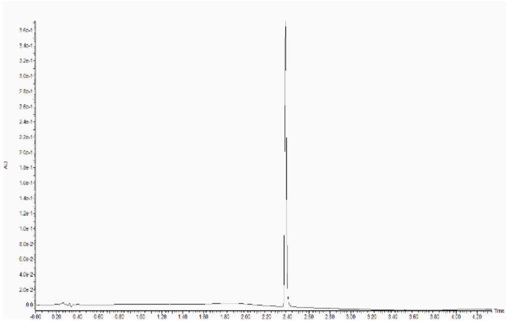	COUPY **1b**<br>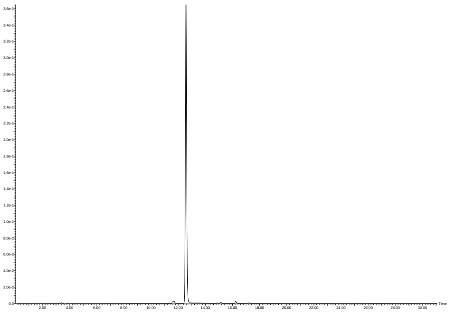
COUPY **1c**<br>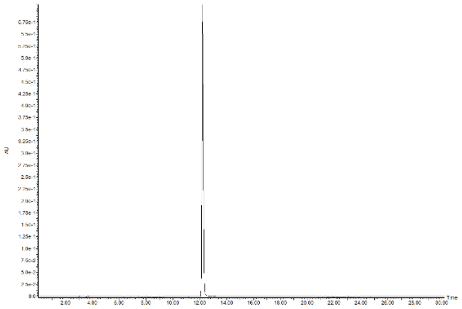	Ir(III) complex **2a**<br>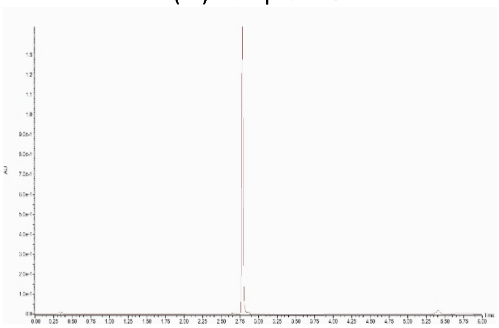
Conjugate **3b**<br>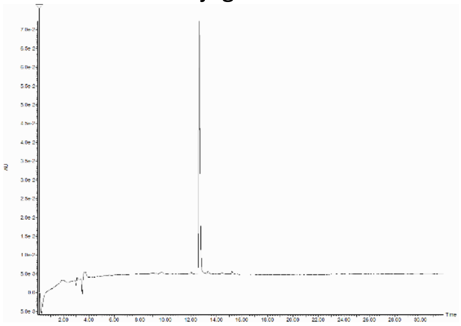	Conjugate **3c**<br>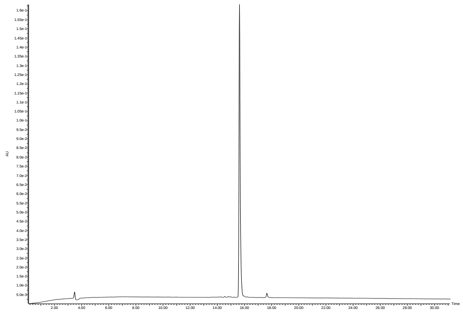
Conjugate **3d**<br>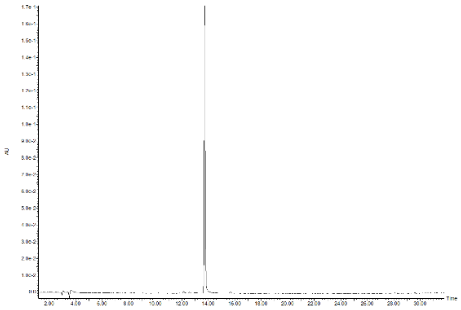	Conjugate **3e**<br>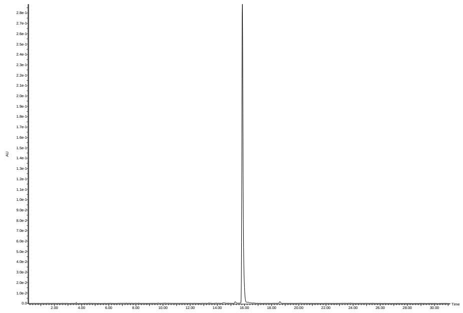

Figure S41. Reversed-phase HPLC analysis of the compounds investigated.
